# Epidemiological features and temporal trends of the co-infection between HIV and tuberculosis, 1990–2021: findings from the Global Burden of Disease Study 2021

**DOI:** 10.1186/s40249-024-01230-3

**Published:** 2024-08-16

**Authors:** Shun-Xian Zhang, Ji-Chun Wang, Jian Yang, Shan Lv, Lei Duan, Yan Lu, Li-Guang Tian, Mu-Xin Chen, Qin Liu, Fan-Na Wei, Xin-Yu Feng, Guo-Bing Yang, Yong-Jun Li, Yu Wang, Xiao-Jie Hu, Ming Yang, Zhen-Hui Lu, Shao-Yan Zhang, Shi-Zhu Li, Jin-Xin Zheng

**Affiliations:** 1 Longhua Hospital, Shanghai University of Traditional Chinese Medicine, Shanghai, 200032 China; 2 National Institute of Parasitic Diseases at Chinese Center for Disease Control and Prevention (Chinese Center for Tropical Diseases Research), NHC Key Laboratory of Parasite and Vector Biology, WHO Collaborating Centre for Tropical Diseases, National Center for International Research on Tropical Diseases; National Key Laboratory of Intelligent Tracking and Forecasting for Infectious Diseases, Shanghai, 200025 China; 3 Department of Science and Technology, Chinese Center for Disease Control and Prevention, National Key Laboratory of Intelligent Tracking and Forecasting for Infectious Diseases, Beijing, 102206 China; 4 School of Global Health, Chinese Center for Tropical Diseases Research-Shanghai Jiao Tong University School of Medicine, Shanghai, 200025 China; 5 Gansu Provincial Center for Disease Control and Prevention, Lanzhou, 730000 China

**Keywords:** Co-infection, Tuberculosis, HIV/AIDS, Epidemiology, Global burden of disease 2021

## Abstract

**Background:**

The co-infection of human immunodeficiency virus (HIV)/acquired immune deficiency syndrome (AIDS) and tuberculosis (TB) poses a significant clinical challenge and is a major global public health issue. This study aims to elucidate the disease burden of HIV-TB co-infection in global, regions and countries, providing critical information for policy decisions to curb the HIV-TB epidemic.

**Methods:**

The ecological time-series study used data from the Global Burden of Disease (GBD) Study 2021. The data encompass the numbers of incidence, prevalence, mortality, and disability-adjusted life year (DALY), as well as age-standardized incidence rate (ASIR), prevalence rate (ASPR), mortality rate (ASMR), and DALY rate for HIV-infected drug-susceptible tuberculosis (HIV-DS-TB), HIV-infected multidrug-resistant tuberculosis (HIV-MDR-TB), and HIV-infected extensively drug-resistant tuberculosis (HIV-XDR-TB) from 1990 to 2021. from 1990 to 2021. The estimated annual percentage change (EAPC) of rates, with 95% confidence intervals (*CI*s), was calculated.

**Results:**

In 2021, the global ASIR for HIV-DS-TB was 11.59 per 100,000 population (95% UI: 0.37–13.05 per 100,000 population), 0.55 per 100,000 population (95% UI: 0.38–0.81 per 100,000 population), for HIV-MDR-TB, and 0.02 per 100,000 population (95% UI: 0.01–0.03 per 100,000 population) for HIV-XDR-TB. The EAPC for the ASIR of HIV-MDR-TB and HIV-XDR-TB from 1990 to 2021 were 4.71 (95% *CI:* 1.92–7.59) and 13.63 (95% *CI:* 9.44–18.01), respectively. The global ASMR for HIV-DS-TB was 2.22 per 100,000 population (95% UI: 1.73–2.74 per 100,000 population), 0.21 per 100,000 population (95% UI: 0.09–0.39 per 100,000 population) for HIV-MDR-TB, and 0.01 per 100,000 population (95% UI: 0.00–0.03 per 100,000 population) for HIV-XDR-TB in 2021. The EAPC for the ASMR of HIV-MDR-TB and HIV-XDR-TB from 1990 to 2021 were 4.78 (95% *CI:* 1.32–8.32) and 10.00 (95% *CI:* 6.09–14.05), respectively.

**Conclusions:**

The findings indicate that enhancing diagnostic and treatment strategies, strengthening healthcare infrastructure, increasing access to quality medical care, and improving public health education are essential to combat HIV-TB co-infection.

**Graphical Abstract:**

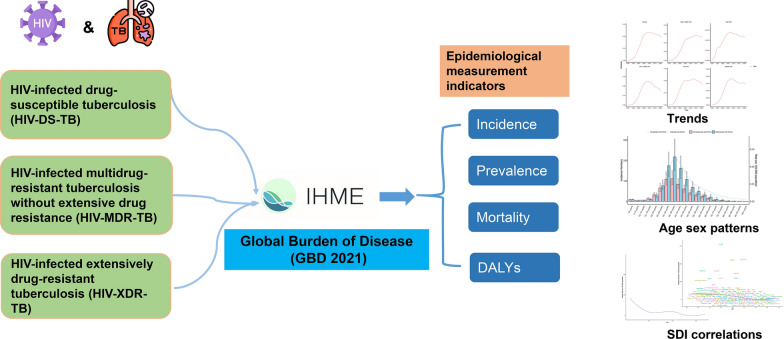

**Supplementary Information:**

The online version contains supplementary material available at 10.1186/s40249-024-01230-3.

## Background

Acquired immune deficiency syndrome (AIDS) is a chronic, systemic, and fatal infectious disease caused by the human immunodeficiency virus (HIV) [1]. HIV disrupts the function of immune T cells and macrophages, particularly reducing the levels and activity of CD4^ + ^T lymphocytes, leading to immunosuppression and opportunistic infections (OPIs) [2, 3]. Tuberculosis (TB), caused by *Mycobacterium tuberculosis* (*Mtb*), is one of the most common OPIs in persons living with HIV (PLWH). Primarily a respiratory disease, TB is chronic and wasting, with a prolonged course often resulting in fatal outcomes [1].

HIV and TB pose significant global public health challenges, contributing substantially to morbidity and mortality worldwide [4–6]. In 2021, there were 7.5 million newly diagnosed and officially notified TB cases globally, with 0.63 million individuals co-infected with HIV [5, 6]. Despite the implementation of standardized TB chemotherapy over 30 years ago, TB remains the leading cause of death from a single infectious agent. In 2021, TB caused 1.3 million deaths, including 1.13 million among HIV-negative individuals and 0.17 million among PLWH [5, 6]. Globally, approximately 39.0 million individuals were PLWH in 2023, and 0.63 million died from AIDS-related illnesses[6].

In nature, host species often harbor multiple pathogens, making co-infection common [7, 8]. The interaction between different microorganisms can alter infection outcomes and significantly impact disease progression. Co-infection with HIV and TB leads to more severe clinical symptoms and faster disease progression compared to single infections [7]. Patients co-infected with HIV and TB have a significantly higher mortality risk than those with either infection alone, earning the designation "deadly human co-infection" [1, 6]. Despite the standardized treatment of PLWH using highly active antiretroviral therapy (HAART), the mortality rate for HIV-positive TB patients was 5.96 times higher than for HIV-negative TB cases [5, 6].

A comprehensive understanding of the burden and epidemiological trends of HIV-TB co-infection is crucial for assessing progress towards ending the epidemic and guiding policy formulation and program implementation [1, 5, 6]. The Global Burden of Disease (GBD) Study 2021 evaluates disease burden by examining rates and numbers of incidence, prevalence, deaths, and disability-adjusted life years (DALYs) worldwide [9, 10]. It provides essential foundational data, enabling the exploration of the epidemiological characteristics of HIV-TB co-infection. The study aims to describe the epidemiological features of HIV-associated drug-susceptible tuberculosis (HIV-DS-TB), HIV-associated multidrug-resistant tuberculosis (HIV-MDR-TB), and extensively drug-resistant tuberculosis in HIV-positive individuals (HIV-XDR-TB) across regions, countries and territories from 1990 to 2021 [1, 9, 10]. These conclusions underscore the urgency of HIV-TB control within the global health framework and provide a scientific basis for developing more effective public health strategies and programs to curb HIV-TB transmission.

## Methods

### Date source

The GBD Study 2021 scientifically and comprehensively evaluated the burden of diseases, injuries, and risk factors across different age and gender groups globally, providing data on 371 diseases or injuries and 88 risk factors from 204 countries and territories spanning 1990 to 2021 [1, 9, 10]. The study utilized the Disease Modeling-Bayesian meta-regression (DisMod-MR) tool (version 2.1), employing Bayesian priors, regularization, and trimming (MR-BRT) modeling. This tool integrated all available morbidity and mortality data, along with epidemiological and spatial relationships, to produce internally consistent disease burden estimates. Detailed information on the design, data collection, and estimation methods is available elsewhere [1, 9, 10].

For HIV-DS-TB, HIV-MDR-TB, and HIV-XDR-TB, data on incident cases, incidence rates, prevalence numbers, prevalence rates, death numbers, mortality rates, DALYs numbers, and DALY rates were obtained from the Institute for Health Metrics and Evaluation (IHME) website (https://vizhub.healthdata.org. Additional file 1). These indices were categorized by year, age group, gender, region, and country or territory.

The GBD 2021 estimated various risk factors for mortality and DALYs [9, 10]. Data on age-standardized mortality rates (ASMRs) and age-standardized DALY rates due to risk factors (level 2) were utilized in this study (Additional file 1). The Socio-demographic Index (SDI) was calculated in GBD 2021 to represent the combined level of health-related social and economic conditions in each region. The SDI values were scaled from 0.00 to 1.00 and multiplied by 100, categorize countries and territories into five development levels: low (< 0.46), low-middle (0.46–0.60), middle (0.61–0.69), high-middle (0.70–0.81), and high (> 0.81) [9, 10].

### Case definition

The International Classification of Diseases (ICD)-10 code for HIV-TB co-infection is B20.0 [9, 10]. HIV-DS-TB is defined as tuberculosis in HIV-positive individuals that is susceptible to both isoniazid and rifampicin. HIV-MDR-TB refers to tuberculosis in HIV-positive individuals that is resistant to the two most effective first-line anti-tuberculosis drugs, isoniazid and rifampicin, but not resistant to any fluoroquinolones or second-line injectable drugs (amikacin, kanamycin, or capreomycin). HIV-XDR-TB is tuberculosis in HIV-positive individuals that is resistant to isoniazid, rifampicin, any fluoroquinolone, and at least one second-line injectable drug (Additional file 1) [1, 9, 10].

### Statistical analysis

The disease burden of HIV-DS-TB, HIV-MDR-TB, and HIV-XDR-TB was quantified using the age-standardized incidence rates (ASIRs), age-standardized prevalence rates (ASPRs), ASMRs, and age-standardized DALY rates, and the numbers for incidence, prevalence, death, and DALYs were also recorded. Age-standardized rates (ASRs, per 100,000 people) from all age groups, specific rates from specific age groups, and numbers were extracted from the GBD 2021 database. Corresponding data are presented as estimates with 95% uncertainty intervals *(*UIs) [9, 10]. The formula for calculating ASR is:

$$ASR\, = \,\frac{{\sum\nolimits_{i\, = \,1}^{N} {a_{i} w_{i} } }}{{\sum\nolimits_{i\, = \,1}^{N} {w_{i} } }}$$

where $a_{i}$ the age-specific rate in the $i$^th^ age group and $w_{{\text{i}}}$ is the number of people in the standard population within each age group. $N$ represents the number of age groups. The 95% UIs were defined as the 2.5th and 97.5th values of the ordered 1000 draws.

The percentage changes in numbers and rates (incidence, prevalence, death, and DALYs) from 1990 to 2021 were calculated using the formula [1, 9, 10]:

Percentage changes = (value _behind_–value _before_)/value _before_ × 100%. The GBD database used UIs instead of precise statistical values. Consequently, when comparing two numerical values (numbers, rates, or percentages), Statistical significance could not be directly calculated; if the UIs overlapped, it indicated no significant difference (*P* > 0.05). Conversely, if the UIs did not overlap, a statistical difference existed (*P* < 0.05).

Smoothing spline models were used to evaluate the association between ASRs (ASIRs, ASMRs, ASPRs, age-standardized DALY rates for HIV-DS-TB, HIV-MDR-TB, and HIV-XDR-TB) and the SDI across global, five SDI regions, 21 geographical regions, 204 countries and territories. Smooth splines using the Locally Weighted Scatterplot Smoothing method were fitted, which automatically determines the degree, number, and location of knots based on the data and the span parameter [1, 9–11]. Spearman's rank correlation coefficient was used to verify the correlations between ASRs and SDI. A *P*-value of less than 0.05 was considered statistically significant.

The estimated annual percentage change (EAPC) of ASRs was calculated to describe the trend fluctuation of HIV-DS-TB, HIV-MDR-TB, and HIV-XDR-TB from 1990 to 2021. It involved a linear regression model $y = \alpha + \beta x + \varepsilon$, where $y$ is equal to natural logarithm of (ASR), $x$ signifies the calendar year, and $\varepsilon$ denotes an independent, normally distributed error term [4]. The EAPC is then calculated as 100 × (e^β^ – 1), the EAPC and their 95% confidence intervals (*CI*s) are utilized to describe trends over specified time intervals [τ_*j—1*_, τ_*j*_]. If the upper limit of the EAPC (95% *CIs*) is less than zero, the rate exhibits a statistically significant declining trend over the observed period. Conversely, if the lower limit of the EAPC (95% *CI*s) is greater than zero, the rate shows a statistically significant increasing trend. When the EAPC (95% *CI*) includes 0, the change in ASR is considered statistically non-significant, indicating that the observed trend is not statistically different from no change [4].

The Bayesian age-period-cohort (BAPC) model, using default parameters, was employed to examine the multiplicative effects of age, period, and cohort [11, 12]:

$\eta_{ij} \, = \,\mu \, + \,\alpha_{i} + \,\beta_{j} \, + \,\gamma_{k}$. In this model, $\eta_{ij}$ stand for the ASR, $\mu$ denotes the intercept, and $\alpha_{i}$ and $\gamma_{k}$ were age, period, and cohort effects, respectively.

All statistical analyses were conducted using R software (version 4.4.1. R Foundation for Statistical Computing, Vienna, Austria. https://cran.r-project.org).

## Results

### Incidence and temporal trend

In 2021, the global ASIR for HIV-DS-TB was 11.59 per 100,000 population (95% UI: 10.37–13.05 per 100,000 population). For HIV-MDR-TB, the ASIR was 0.55 per 100,000 population (95% UI: 0.38–0.81 per 100,000 population), and for HIV-XDR-TB, it was 0.02 per 100,000 population (95% UI: 0.01–0.03 per 100,000 population. Table 1). Additionally, the EAPC for the ASIR of HIV-DS-TB and HIV-MDR-TB from 1990 to 2021 were −0.67 (95% *CI:* −1.81, 0.05) and 4.71 (95% *CI:* 1.92–7.59), respectively. The EAPC for the ASIR of HIV-XDR-TB from 1991 to 2021 was 13.63 (95% *CI:* 9.44–18.01).

**Table 1:** ASIR (per 100,000 population) of HIV-DS-TB, HIV-MDR-TB, and HIV-XDR-TB in 2021, and percentage change of ASIR were analyzed across GBD regions

Region	HIV-DS-TB		HIV-MDR-TB		HIV-XDR-TB	
	ASIR (95% UI) 2021	Percentage change of ASIR (95% UI) 1990–2021	ASIR (95% UI) 2021	Percentage change of ASIR (95% UI) 1990–2021	ASIR (95% UI) 2021	Percentage change of ASIR (95% UI) 2010–2021
Global	11.59 (10.37–13.05)	0.18 (0.12–0.25)	0.55 (0.38–0.81)	14.44 (7.52–25.68)	0.02 (0.01–0.03)	−0.16 (−0.36, 0.10)
Male	10.03 (9.00–11.28)	0.15 (0.10–0.22)	0.52 (0.36–0.76)	12.29 (6.57–21.56)	0.02 (0.02–0.03)	−0.17 (−0.38, 0.11)
Female	13.19 (11.79–14.85)	0.20 (0.12–0.28)	0.59 (0.41–0.87)	16.91 (8.35–31.44)	0.02 (0.01–0.02)	−0.13 (−0.35, 0.12)
East Asia	1.21 (1.03–1.38)	0.94 (0.62–1.59)	0.06 (0.01–0.19)	1.36 (−0.64, 11.37)	0.01 (0.00–0.02)	0.14 (−0.70, 2.05)
Southeast Asia	6.60 (5.93–7.27)	1.80 (1.65–1.95)	0.23 (0.12–0.40)	17.68 (5.59–57.02)	0.02 (0.01–0.04)	−0.40 (−0.71, 0.09)
Oceania	9.66 (8.57–10.78)	14.49 (12.14–21.37)	0.44 (0.13–1.07)	953.96 (195.84–3300.29)	0.06 (0.02–0.15)	2.27 (−0.30, 10.19)
Central Asia	0.62 (0.48–0.75)	−0.34 (−0.47, −0.21)	0.24 (0.15–0.36)	124.84 (39.91–356.2)	0.05 (0.03–0.08)	−0.21 (−0.49, 0.15)
Central Europe	0.30 (0.26–0.35)	−0.40 (−0.48, −0.22)	0.01 (0.00–0.02)	0.60 (−0.49, 4.04)	0.00 (0.00–0.00)	−0.20 (−0.75, 1.28)
Eastern Europe	2.47 (1.77–3.30)	−0.02 (−0.27, 0.21)	1.48 (0.90–2.25)	38.14 (14.13–94.89)	0.31 (0.19–0.48)	0.06 (−0.37, 0.71)
High-income Asia Pacific	0.28 (0.24–0.33)	−0.25 (−0.41, 1.93)	0.00 (0.00–0.01)	0.43 (−0.75, 5.44)	0.00 (0.00–0.00)	−0.03 (−0.68, 1.89)
Australasia	0.11 (0.10–0.13)	−0.84 (−0.85, −0.83)	0.00 (0.00–0.01)	−0.02 (−0.76, 3.67)	0.00 (0.00–0.00)	0.93 (−0.46, 5.73)
Western Europe	0.26 (0.22–0.29)	−0.85 (−0.85, −0.84)	0.01 (0.00–0.01)	−0.61 (−0.81, −0.21)	0.00 (0.00–0.00)	−0.10 (−0.45, 0.47)
Southern Latin America	3.74 (3.23–4.36)	−0.26 (−0.32, −0.20)	0.06 (0.02–0.19)	2.04 (−0.47, 16.05)	0.01 (0.00–0.03)	0.01 (−0.71, 1.62)
High-income North America	0.16 (0.14–0.19)	−0.82 (−0.83, −0.80)	0.00 (0.00–0.01)	−0.93 (−0.97, −0.77)	0.00 (0.00–0.00)	0.29 (−0.47, 2.24)
Caribbean	5.68 (5.01–6.41)	−0.48 (−0.53, −0.44)	0.03 (0.01–0.08)	−0.41 (−0.86, 1.43)	0.00 (0.00–0.01)	0.11 (−0.64, 2.20)
Andean Latin America	4.02 (3.41–4.69)	−0.31 (−0.40, −0.19)	0.35 (0.16–0.72)	5.67 (1.00–24.67)	0.03 (0.01–0.06)	−0.14 (−0.56, 0.70)
Central Latin America	2.21 (1.95–2.53)	−0.36 (−0.41, −0.30)	0.08 (0.04–0.17)	12.91 (3.68–39.53)	0.01 (0.00–0.02)	0.22 (−0.42, 1.37)
Tropical Latin America	5.40 (4.56–6.34)	−0.34 (−0.41, −0.28)	0.22 (0.05–0.60)	45.28 (4.77–430.61)	0.02 (0.00–0.05)	0.57 (−0.60, 3.46)
North Africa and Middle East	0.78 (0.69–0.90)	0.33 (0.16–0.57)	0.02 (0.01–0.04)	12.28 (3.97–34.63)	0.00 (0.00–0.00)	0.15 (−0.44, 1.08)
South Asia	3.68 (3.06–4.32)	4.90 (4.03–5.84)	0.34 (0.09–0.81)	483.32 (54.9–5132.99)	0.01 (0.00–0.02)	−0.41 (−0.82, 0.51)
Central sub-Saharan Africa	42.35 (37.64–47.42)	−0.47 (−0.51, −0.44)	1.11 (0.41–2.42)	3.21 (0.00–20.43)	0.01 (0.00–0.02)	−0.45 (−0.81, 0.61)
Eastern sub-Saharan Africa	88.11 (77.54–101.08)	−0.45 (−0.49, −0.40)	3.82 (2.16–6.26)	26.38 (9.10–69.85)	0.03 (0.02–0.05)	−0.16 (−0.52, 0.45)
Southern sub-Saharan Africa	415.95 (369.19–468.18)	0.93 (0.74–1.13)	16.40 (7.79–35.65)	18.05 (4.37–79.09)	0.12 (0.06–0.26)	−0.34 (−0.76, 0.61)
Western sub-Saharan Africa	33.03 (28.92–37.33)	−0.25 (−0.32, −0.16)	1.34 (0.61–2.78)	4.86 (1.02–16.60)	0.01 (0.01–0.02)	−0.37 (−0.68, 0.23)
High SDI	0.25 (0.22–0.29)	−0.73 (−0.74, −0.71)	0.01 (0.01–0.01)	−0.63 (-0.80, −0.32)	0.00 (0.00–0.00)	−0.12 (−0.36, 0.26)
High-middle SDI	1.17 (0.99–1.38)	−0.09 (−0.18, 0.01)	0.26 (0.16–0.40)	14.91 (7.27–32.89)	0.05 (0.03–0.08)	0.01 (−0.38, 0.56)
Middle SDI	13.82 (12.42–15.51)	2.50 (2.21–2.82)	0.55 (0.28–1.12)	15.54 (4.65–49.34)	0.01 (0.01–0.02)	−0.16 (−0.50, 0.38)
Low-middle SDI	13.69 (12.08–15.72)	−0.09 (−0.15, −0.01)	0.71 (0.41–1.10)	19.61 (5.22–65.62)	0.01 (0.01–0.02)	−0.39 (−0.62, −0.04)
Low SDI	34.95 (30.88–39.44)	−0.47 (−0.51, −0.43)	1.52 (0.90–2.41)	9.97 (3.51–23.21)	0.02 (0.01–0.03)	−0.20 (−0.50, 0.26)

Globally, the World Health Organization began to recommend the XDR-TB surveillance in 1991. Consequently, the ASIR of HIV-XDR-TB has been tracked and reported since 1991. However, the GBD 2021 database provides total percentage change data for the periods 1990–2000, 2000–2021, 1990–2021, 2010–2021, and 2019–2021. Therefore, percentage change of ASIRs for HIV-XDR-TB spanning 2010–2021 was used in the study

*ASIR* Age-standardized incidence rate, *GBD* Global Burden of Disease, *HIV-DS-TB* HIV-infected drug-susceptible tuberculosis, *HIV-MDR-TB* HIV-infected. multidrug-resistant tuberculosis without extensive drug resistance, *HIV-XDR-TB* HIV-infected extensively drug-resistant tuberculosis, *SDI* Sociodemographic Index, *UI* uncertainty intervals

In 2021, the global incidence of HIV-DS-TB was 955,221 individuals (95% UI: 854,661–1,075,240 individuals), HIV-MDR-TB was 45,589 individuals (95% UI: 31,326–66,723 individuals), and HIV-XDR-TB was 1606 individuals (95% UI: 1164–2183 individuals) (Additional file 1: Table S1).

In 2021, the ASIR for HIV-DS-TB was 13.19 per 100,000 population (95% UI: 11.79–14.85 per 100,000 population) among females, compared to 10.03 per 100,000 population (95% UI: 9.00–11.28 per 100,000 population) among males. The ASIR for HIV-DS-TB was higher in females than in males (*P* < 0.05. Table 1). However, there were no significant differences in the ASIR for HIV-MDR-TB and HIV-XDR-TB between males and females (all *P* > 0.05. Table 1). Compared to 1990, the ASIR for both HIV-DS-TB and HIV-MDR-TB increased in both genders, but the increase in ASIR for HIV-MDR-TB was significantly higher than that for HIV-DS-TB in both males and females (both *P* < 0.05. Table 1).

In 2021, the highest ASIR for HIV-DS-TB and HIV-MDR-TB was observed in the low SDI region, while the lowest ASIR was in the high SDI region. For HIV-XDR-TB, the highest ASIR was recorded in the high-middle SDI region, with the lowest in the high SDI region (Table 1). From 1990 to 2021, the ASIR for HIV-DS-TB began to decline across all five SDI regions after 2005. For HIV-MDR-TB, the ASIR remained stable only in the middle SDI region, while it decreased in the other four SDI regions. In recent years, the ASIR of HIV-XDR-TB has remained relatively stable in the high, high-middle, and middle SDI regions (Additional file 1: Fig. S1 A–C).

In 2021, the highest ASIR for HIV-DS-TB and HIV-MDR-TB were recorded in sub-Saharan Africa, while the highest ASIR for HIV-XDR-TB was observed in Eastern Europe. Compared to 1990, regions with increasing ASIR for HIV-DS-TB included Oceania, South Asia, Southeast Asia, and North Africa and the Middle East (all *P* < 0.05. Table 1). Regions where the ASIR for HIV-DS-TB decreased included Central Asia, Central Europe, Australasia, Western Europe, Southern Latin America, high-income North America, the Caribbean, Andean Latin America, Central Latin America, and Tropical Latin America (all *P* < 0.05. Table 1). For HIV-MDR-TB, the largest increase in ASIR was observed in Oceania, followed by South Asia and Central Asia (all *P* < 0.05. Table 1). Conversely, the ASIR for HIV-MDR-TB decreased in Western Europe and high-income North America (both *P* < 0.05. Table 1).

In 2021, the country with the highest ASIR for HIV-DS-TB was Lesotho. For HIV-MDR-TB and HIV-XDR-TB, the highest ASIR was observed in Eswatini. Compared to 1990, the country with the greatest increase in ASIR for both HIV-DS-TB and HIV-MDR-TB in 2021 was Pakistan (both *P* < 0.05. Additional file 1: Table S2).

### Prevalence and temporal trend

In 2021, the global ASPR for HIV-DS-TB was 20.41 per 100,000 population (95% UI: 18.14–22.82 per 100,000 population). For HIV-MDR-TB, the ASPR was 0.87 per 100,000 population (95% UI: 0.59–1.29 per 100,000 population), and for HIV-XDR-TB, it was 0.02 per 100,000 population (95% UI: 0.02–0.03 per 100,000 population. Table 2). The EAPC for the ASPR of HIV-DS-TB and HIV-MDR-TB from 1990 to 2021 were −0.71 (95% *CI:* −1.77, 0.37) and 4.97 (95% *CI:* 2.32–7.70), respectively, while the EAPC for the ASPR of HIV-XDR-TB from 1991 to 2021 was 13.79 (95% *CI:* 10.03–17.67).

**Table 2:** ASPR (per 100,000 population) of HIV-DS-TB, HIV-MDR-TB, and HIV-XDR-TB in 2021, and percentage change of ASPR were analyzed across GBD regions

Region	HIV-DS-TB		HIV-MDR-TB		HIV-XDR-TB	
	ASPR (95% UI) 2021	Percentage change of ASPR (95% UI) 1990–2021	ASPR (95% UI) 2021	Percentage change of ASPR (95% UI) 1990–2021	ASPR (95% UI) 2021	Percentage change of ASPR (95% UI) 2010–2021
Global	20.41 (18.14–22.82)	0.09 (0.03–0.17)	0.87 (0.59–1.29)	15.93 (8.37–28.21)	0.02 (0.02–0.03)	−0.07 (−0.31, 0.27)
Male	17.33 (15.48–19.25)	0.10 (0.04–0.17)	0.79 (0.53–1.15)	14.33 (7.51–24.69)	0.02 (0.02–0.04)	−0.10 (−0.34, 0.28)
Female	23.56 (20.85–26.41)	0.08 (0.01–0.17)	0.95 (0.65–1.40)	17.45 (8.78–32.23)	0.02 (0.01–0.02)	−0.04 (−0.27, 0.28)
East Asia	2.42 (2.00–2.80)	1.60 (1.03–2.41)	0.13 (0.02–0.39)	3.08 (−0.46, 26.32)	0.01 (0.00–0.03)	0.26 (−0.71, 2.51)
Southeast Asia	12.32 (10.97–13.66)	2.26 (2.00–2.62)	0.42 (0.21–0.73)	23.46 (7.15–78.24)	0.04 (0.02–0.06)	−0.26 (−0.63, 0.37)
Oceania	33.11 (29.29–36.89)	29.05 (20.68–39.56)	1.39 (0.40–3.30)	2169.85 (487.4–7568.75)	0.12 (0.04–0.29)	3.06 (−0.15, 13.79)
Central Asia	0.86 (0.67–1.07)	−0.42 (−0.54, −0.28)	0.28 (0.18–0.43)	124.89 (39.17–391.81)	0.06 (0.04–0.09)	−0.18 (−0.47, 0.21)
Central Europe	0.37 (0.32–0.43)	−0.41 (−0.52, −0.11)	0.01 (0.00–0.02)	1.18 (−0.27, 5.47)	0.00 (0.00–0.00)	−0.06 (−0.68, 1.48)
Eastern Europe	2.26 (1.62–2.99)	−0.06 (−0.30, 0.17)	1.07 (0.67–1.57)	28.29 (10.91–67.41)	0.23 (0.14–0.33)	−0.04 (−0.42, 0.53)
High-income Asia Pacific	0.20 (0.16–0.24)	−0.45 (−0.57, 0.78)	0.00 (0.00–0.01)	0.10 (−0.83, 8.57)	0.00 (0.00–0.00)	−0.09 (−0.71, 1.68)
Australasia	0.09 (0.07–0.10)	−0.86 (−0.87, −0.85)	0.00 (0.00–0.01)	−0.17 (−0.79, 2.83)	0.00 (0.00–0.00)	0.71 (−0.50, 4.59)
Western Europe	0.28 (0.24–0.33)	−0.87 (−0.88, −0.86)	0.01 (0.00–0.01)	−0.56 (−0.78, −0.11)	0.00 (0.00–0.00)	−0.05 (−0.39, 0.54)
Southern Latin America	4.86 (4.19–5.58)	−0.34 (−0.39, −0.29)	0.07 (0.02–0.23)	1.73 (−0.49, 13.96)	0.01 (0.00–0.03)	−0.01 (−0.71, 1.69)
High-income North America	0.21 (0.17–0.24)	−0.84 (−0.86, −0.83)	0.00 (0.00–0.01)	−0.92 (−0.97, −0.74)	0.00 (0.00–0.00)	0.29 (−0.49, 2.31)
Caribbean	7.92 (6.77–9.19)	−0.50 (−0.54, −0.46)	0.04 (0.02–0.10)	−0.20 (−0.78, 1.47)	0.00 (0.00–0.01)	0.30 (−0.53, 2.34)
Andean Latin America	5.07 (4.27–5.96)	−0.35 (−0.46, −0.23)	0.41 (0.18–0.84)	5.35 (0.91–23.45)	0.03 (0.01–0.06)	−0.02 (−0.51, 0.93)
Central Latin America	3.53 (3.05–4.06)	−0.41 (−0.47, −0.35)	0.12 (0.05–0.26)	14.83 (4.03–46.39)	0.01 (0.00–0.02)	0.47 (−0.33, 1.98)
Tropical Latin America	5.92 (5.04–6.87)	−0.49 (−0.55, −0.44)	0.21 (0.05–0.61)	37.95 (4.16–379.96)	0.02 (0.00–0.05)	0.69 (−0.57, 3.87)
North Africa and Middle East	0.91 (0.79–1.04)	0.39 (0.20–0.63)	0.02 (0.01–0.04)	18.98 (7.13–50.98)	0.00 (0.00–0.00)	0.38 (−0.31, 1.59)
South Asia	5.70 (4.76–6.68)	5.48 (4.37–6.81)	0.48 (0.13–1.14)	581.21 (63.4–7557.26)	0.01 (0.00–0.03)	−0.19 (−0.75, 1.12)
Central sub-Saharan Africa	93.62 (83.18–104.7)	−0.51 (-0.54, −0.47)	2.38 (0.89–5.4)	3.17 (−0.04, 20.91)	0.01 (0.01–0.03)	−0.17 (−0.70, 1.43)
Eastern sub-Saharan Africa	165.66 (144.49–187.93)	−0.51 (−0.55, −0.47)	6.70 (3.88–11.16)	25.58 (9.47–67.97)	0.04 (0.02–0.07)	0.33 (−0.27, 1.32)
Southern sub-Saharan Africa	666.44 (589.51–755.78)	1.15 (0.93–1.43)	25.07 (11.59–53.46)	23.7 (5.27–108.91)	0.16 (0.07–0.33)	−0.06 (−0.67, 1.31)
Western sub-Saharan Africa	66.37 (57.78–75.03)	−0.31 (−0.38, −0.22)	2.47 (1.17–4.88)	4.24 (0.88–14.34)	0.02 (0.01–0.03)	−0.24 (−0.62, 0.44)
High SDI	0.30 (0.26–0.35)	−0.77 (−0.78, −0.76)	0.01 (0.01–0.02)	−0.55 (−0.75, −0.17)	0.00 (0.00–0.00)	−0.05 (−0.33, 0.38)
High-middle SDI	1.70 (1.46–1.96)	0.11 (0.02–0.23)	0.22 (0.14–0.34)	12.75 (5.64–29.07)	0.04 (0.03–0.06)	−0.08 (−0.43, 0.40)
Middle SDI	22.22 (19.82–24.89)	2.80 (2.40–3.24)	0.84 (0.42–1.69)	20.64 (6.55–63.32)	0.02 (0.01–0.03)	0.00 (−0.42, 0.73)
Low-middle SDI	24.21 (21.32–27.66)	−0.11 (−0.18, −0.04)	1.12 (0.66–1.72)	19.31 (5.37–61.56)	0.02 (0.01–0.03)	−0.22 (−0.54, 0.31)
Low SDI	68.88 (60.48–77.35)	−0.51 (−0.55, −0.47)	2.78 (1.69–4.31)	9.38 (3.28–21.86)	0.02 (0.01–0.03)	0.17 (−0.27, 0.82)

Globally, the World Health Organization began to recommend the XDR-TB surveillance. Consequently, the age-standardized prevalence rate of HIV-XDR-TB has been tracked and reported since 1991. However, the GBD 2021 database provides total percentage change data for the periods 1990–2000, 2000–2021, 1990–2021, 2010–2021, and 2019–2021. Therefore, percentage change of ASPR for HIV-XDR-TB spanning 2010–2021 were used in the study

*ASPR* Age-standardized prevalence rate, *GBD* Global Burden of Disease, *HIV-DS-TB* HIV-infected drug-susceptible tuberculosis, *HIV-MDR-TB* HIV-infected. multidrug-resistant tuberculosis without extensive drug resistance, *HIV-XDR-TB* HIV-infected extensively drug-resistant tuberculosis, *SDI* Sociodemographic Index, *UI* uncertainty intervals

In 2021, the global prevalence of HIV-DS-TB was 1,682,115 cases (95% UI: 1,494,990–1,881,082 persons), the prevalence of HIV-MDR-TB was 71,455 cases (95% UI: 48,999–106,009 persons), and the prevalence of HIV-XDR-TB was 1727 cases (95% UI: 1241–2427 persons. Additional file 1: Table S3).

In 2021, the ASPR for HIV-DS-TB, HIV-MDR-TB and HIV-XDR-TB showed no significant differences between genders (all *P* > 0.05). Compared to 1990, the ASPR of HIV-DS-TB and HIV-MDR-TB increased in both females and males (all *P* < 0.05. Table 2).

In 2021, the highest ASPR for HIV-DS-TB and HIV-MDR-TB was observed in the low SDI region, while the lowest was in the high SDI region. For HIV-XDR-TB, the highest ASPR was found in the high-middle SDI region, and the lowest in the high SDI region. From 1990 to 2021, the ASPR for HIV-DS-TB and HIV-MDR-TB initially increased across all five SDI regions but started to decline after 2010. Conversely, the ASPR for HIV-XDR-TB has been rapidly rising in the low SDI region and slowly increasing in the high and middle SDI regions (Additional file 1: Fig.S2 A–C).

In 2021, the highest ASPR for HIV-DS-TB and HIV-MDR-TB were recorded in Southern sub-Saharan Africa. For HIV-XDR-TB, the highest ASPR was in Eastern Europe. Compared to 1990, regions with increased ASPR for HIV-DS-TB in 2021 included Oceania, South Asia, Southeast Asia, East Asia, and North Africa and the Middle East (all *P* < 0.05. Table 2). Conversely, the ASPR for HIV-DS-TB decreased in Western Europe and high-income North America (both *P* < 0.05. Table 2). The regions with the most significant increases in ASPR for HIV-MDR-TB were Oceania, followed by South Asia and Central Asia (all *P* < 0.05. Table 2). The ASPR for HIV-MDR-TB decreased only in high-income North America (*P* < 0.05, Table 2).

In 2021, the country with the highest ASPR for HIV-DS-TB was Lesotho. For HIV-MDR-TB and HIV-XDR-TB, the highest ASPR were observed in Eswatini. Compared to 1990, the country with the most significant increases in HIV-DS-TB and HIV-MDR-TB prevalence rates by 2021 was Pakistan (both *P* < 0.05. Additional file 1: Table S2).

### Mortality and temporal trend

In 2021, the global ASMR for HIV-DS-TB was 2.22 per 100,000 population (95% UI: 1.73–2.74 per 100,000 population), for HIV-MDR-TB it was 0.21 per 100,000 population (95% UI: 0.09–0.39 per 100,000 population), and for HIV-XDR-TB it was 0.01 per 100,000 population (95% UI: 0.00–0.02 per 100,000 population. Table 3). The EAPC for the ASMR of HIV-DS-TB from 1990 to 2021 was −1.56 (95% *CI:* −3.22, 0.12), and for HIV-MDR-TB it was 4.78 (95% *CI:* 1.32–8.32). The EAPC for the ASMR of HIV-XDR-TB from 1993 to 2021 was 10.00 (95% *CI:* 6.09–14.05).

**Table 3:** ASMR (per 100,000 population) of HIV-DS-TB, HIV-MDR-TB, and HIV-XDR-TB in 2021, and percentage change of ASMR were analyzed across GBD regions

Region	HIV-DS-TB		HIV-MDR-TB		HIV-XDR-TB	
	ASMR (95% UI) 2021	Percentage change of ASMR (95% UI) 1990–2021	ASMR (95% UI) 2021	Percentage change of ASMR (95% UI) 1990–2021	ASMR (95% UI) 2021	Percentage change of ASMR (95% UI) 2010–2021
Global	2.22 (1.73–2.74)	0.04 (−0.22, 0.47)	0.21 (0.09–0.39)	14.58 (8.31–27.16)	0.01 (0.00–0.02)	−0.36 (−0.50, −0.18)
Male	2.09 (1.64–2.57)	0.06 (−0.19, 0.43)	0.20 (0.09–0.37)	12.14 (7.10–21.85)	0.01 (0.01–0.02)	−0.38 (−0.52, −0.20)
Female	2.35 (1.82–2.88)	0.03 (−0.26, 0.51)	0.23 (0.10–0.42)	17.7 (9.05–36.05)	0.01 (0.00–0.01)	−0.32 (−0.48, −0.14)
East Asia	0.17 (0.10–0.25)	1.94 (0.40–21.45)	0.02 (0.00–0.05)	3.01 (−0.51, 78.95)	0.00 (0.00–0.01)	0.00 (−0.74, 1.68)
Southeast Asia	1.22 (0.95–1.52)	2.76 (2.03–3.64)	0.08 (0.03–0.16)	18.84 (4.86–77.43)	0.02 (0.01–0.04)	−0.44 (−0.73, −0.01)
Oceania	2.74 (1.9–3.97)	34.11 (20.73–49.35)	0.25 (0.06–0.64)	1529.45 (369.49–5526.74)	0.05 (0.01–0.12)	2.26 (−0.28, 10.01)
Central Asia	0.09 (0.05–0.16)	−0.60 (−0.75, −0.44)	0.05 (0.02–0.09)	61.99 (19.38–196.68)	0.02 (0.01–0.04)	−0.41 (−0.58, −0.22)
Central Europe	0.06 (0.04–0.09)	−0.56 (−0.62, −0.44)	0.00 (0.00–0.01)	0.21 (−0.63, 3.44)	0.00 (0.00–0.00)	−0.23 (−0.74, 1.20)
Eastern Europe	0.28 (0.13–0.53)	−0.24 (−0.59, 0.14)	0.23 (0.12–0.39)	18.39 (6.99–52.98)	0.11 (0.05–0.18)	−0.39 (−0.58, −0.11)
High-income Asia Pacific	0.04 (0.04–0.05)	0.90 (0.57–1.15)	0.00 (0.00–0.00)	2.11 (−0.28, 11.14)	0.00 (0.00–0.00)	0.05 (−0.64, 1.89)
Australasia	0.01 (0.01–0.02)	−0.90 (−0.92, −0.88)	0.00 (0.00–0.00)	−0.41 (−0.85, 1.69)	0.00 (0.00–0.00)	0.60 (−0.56, 4.33)
Western Europe	0.08 (0.05–0.12)	−0.89 (−0.90, −0.86)	0.00 (0.00–0.01)	−0.67 (−0.84, −0.32)	0.00 (0.00–0.00)	−0.11 (−0.44, 0.43)
Southern Latin America	1.07 (0.70–1.36)	0.53 (0.01–0.93)	0.03 (0.01–0.11)	4.51 (0.00–26.67)	0.01 (0.00–0.03)	0.01 (−0.70, 1.49)
High-income North America	0.04 (0.02–0.06)	−0.90 (−0.92, −0.89)	0.00 (0.00–0.01)	−0.95 (−0.98, −0.86)	0.00 (0.00–0.00)	0.28 (−0.47, 1.99)
Caribbean	1.73 (0.94–4.30)	−0.67 (−0.75, −0.47)	0.02 (0.00–0.06)	−0.50 (−0.89, 1.20)	0.00 (0.00–0.01)	0.09 (−0.66, 2.21)
Andean Latin America	0.73 (0.44–1.18)	0.33 (−0.19, 1.15)	0.13 (0.04–0.29)	10.56 (2.24–43.89)	0.02 (0.01–0.05)	−0.23 (−0.58, 0.36)
Central Latin America	0.58 (0.37–0.86)	−0.52 (−0.62, −0.38)	0.04 (0.01–0.09)	10.41 (3.07–29.66)	0.01 (0.00–0.02)	0.19 (−0.38, 1.15)
Tropical Latin America	0.97 (0.60–1.49)	−0.50 (−0.63, −0.37)	0.07 (0.01–0.23)	31.38 (3.49–308.34)	0.01 (0.00–0.04)	0.42 (−0.61, 2.68)
North Africa and Middle East	0.13 (0.09–0.18)	0.53 (−0.03, 1.50)	0.01 (0.00–0.02)	16.32 (5.39–49.57)	0.00 (0.00–0.00)	0.04 (−0.48, 0.81)
South Asia	0.54 (0.36–0.73)	43.9 (19.26–116.44)	0.09 (0.02–0.21)	3356.9 (493.55–42,803.18)	0.00 (0.00–0.01)	−0.56 (−0.85, 0.00)
Central sub-Saharan Africa	8.92 (6.62–11.85)	−0.54 (−0.71, −0.21)	0.53 (0.16–1.27)	2.78 (−0.20, 20.25)	0.01 (0.00–0.02)	−0.47 (−0.80, 0.49)
Eastern sub-Saharan Africa	19.59 (14.85–24.34)	−0.55 (−0.70, −0.28)	1.81 (0.74–3.72)	27.20 (8.43–88.72)	0.03 (0.01–0.05)	−0.20 (−0.53, 0.38)
Southern sub-Saharan Africa	66.48 (55.76–73.54)	2.05 (1.06–3.58)	6.13 (2.27–13.47)	36.31 (7.69–219.1)	0.09 (0.03–0.21)	−0.41 (−0.76, 0.35)
Western sub-Saharan Africa	7.84 (5.18–11.29)	−0.10 (−0.37, 0.36)	0.67 (0.21–1.58)	4.45 (0.89–17.96)	0.01 (0.00–0.02)	−0.37 (−0.67, 0.18)
High SDI	0.05 (0.03–0.08)	−0.86 (−0.88, −0.84)	0.00 (0.00–0.01)	−0.79 (−0.89, −0.62)	0.00 (0.00–0.00)	−0.21 (−0.43, 0.13)
High-middle SDI	0.19 (0.11–0.28)	−0.16 (−0.30, −0.01)	0.04 (0.02–0.07)	8.10 (3.74–19.23)	0.02 (0.01–0.03)	−0.39 (−0.57, -0.15)
Middle SDI	2.12 (1.68–2.46)	3.51 (2.80–4.31)	0.18 (0.06–0.39)	30.92 (10.80–90.7)	0.01 (0.00–0.02)	−0.26 (−0.53, 0.17)
Low-middle SDI	2.94 (2.16–3.83)	0.15 (−0.22, 0.78)	0.29 (0.12–0.56)	23.65 (7.44–78.47)	0.01 (0.00–0.02)	−0.49 (−0.67, −0.21)
Low SDI	7.63 (5.83–9.34)	−0.56 (−0.69, −0.32)	0.73 (0.30–1.44)	8.88 (3.21–26.21)	0.01 (0.00–0.02)	−0.23 (−0.51, 0.17)

Globally, the World Health Organization began to recommend the XDR-TB surveillance. Consequently, the ASMR of HIV-XDR -TB has been tracked and reported since 1991. However, the GBD 2021 database provides total percentage change data for the periods 1990–2000, 2000–2021, 1990–2021, 2010–2021, and 2019–2021. Therefore, percentage change of ASMR for HIV-XDR-TB spanning 2010–2021 were used in the study

*ASMs* Age-standardized mortality rate, *GBD* Global Burden of Disease, *HIV-DS-TB* HIV-infected drug-susceptible tuberculosis, *HIV-MDR-TB* HIV-infected. multidrug-resistant tuberculosis without extensive drug resistance, *HIV-XDR-TB* HIV-infected extensively drug-resistant tuberculosis, *SDI* Sociodemographic Index, *UI* uncertainty intervals

In 2021, the global number of deaths due to HIV-DS-TB was 182,597 individuals (95% UI: 141,923–225,076 individuals), for HIV-MDR-TB it was 17,458 (95% UI: 7574–32,229 individuals), and for HIV-XDR-TB it was 840 (95% UI: 385–1492 individuals. Additional file 1: Table S4).

In 2021, there was no significant difference in the ASMR for HIV-DS-TB, HIV-MDR-TB and HIV-XDR-TB between males and females (all *P* > 0.05. Table 3). Compared to 1990, the ASMR for HIV-DS-TB did not show significant changes in either gender (*P* > 0.05. Table 3). However, the ASMR for HIV-MDR-TB significantly increased in both males and females (both *P* < 0.05. Table 3).

In 2021, the highest ASMR for HIV-DS-TB and HIV-MDR-TB were observed in the low SDI region, while the lowest rates were in the high SDI region. Conversely, the highest ASMR for HIV-XDR-TB was recorded in the high-middle SDI region, with the lowest in the high SDI region (Table 3). From 1990 to 2021, the ASMR for HIV-DS-TB, HIV-MDR-TB, and HIV-XDR-TB initially increased and then decreased across the five SDI regions (Additional file 1: Fig. S3 A–C). Notably, post-2010, these rates generally declined. However, the decrease in ASMR for HIV-XDR-TB markedly slowed (Additional file 1: Fig. S3 A–C).

In 2021, the highest ASMR for HIV-DS-TB and HIV-MDR-TB was found in Southern sub-Saharan Africa, while the highest ASMR for HIV-XDR-TB was in Eastern Europe. Compared to 1990, regions with an increased ASMR for HIV-DS-TB in 2021 included South Asia, Oceania, Southern sub-Saharan Africa, Southeast Asia, East Asia, high-income Asia Pacific, and Southern Latin America (all *P* < 0.05. Table 3). In contrast, Western Europe and high-income North America experienced a decline in ASMR for HIV-DS-TB (both *P* < 0.05. Table 3). The regions with the largest increase in ASMR for HIV-MDR-TB were South Asia, Oceania, and Central Asia (all *P* < 0.05. Table 3). Conversely, Western Europe and high-income North America were the regions with a decline in ASMR for HIV-MDR-TB (both *P* < 0.05. Table 3).

In 2021, the country with the highest ASMR for HIV-DS-TB was Lesotho. For both HIV-MDR-TB and HIV-XDR-TB, the highest ASMR was in Eswatini. Compared to 1990, the country with the largest increase in ASMR for HIV-DS-TB in 2021 was Pakistan, while the country with the largest increase in ASMR for HIV-MDR-TB was Cambodia (all *P* < 0.05. Additional file 1: Table S2).

### DALY and temporal trend

In 2021, the global age-standardized DALY rate for HIV-DS-TB was 122.54 per 100,000 population (95% UI: 96.79–149.60 per 100,000 population), for HIV-MDR-TB it was 11.48 per 100,000 population (95% UI: 5.31–20.78 per 100,000 population), and for HIV-XDR-TB it was 0.51 per 100,000 population (95% UI: 0.24–0.91 per 100,000 population. Table 4). In addition, the EAPC for the age-standardized DALY rates of HIV-DS-TB and HIV-MDR-TB from 1990 to 2021 were −1.74 (95% *CI:* −3.36, −0.09) and 4.65 (95% *CI:* 1.25–8.17), respectively. The EAPC for the age-standardized DALY rate of HIV-XDR-TB from 1991 to 2021 was 19.35 (95% *CI:* 10.93–28.42).

**Table 4:** Age-standardized DALY rate (per 100,000 population) of HIV-DS-TB, HIV-MDR-TB, and HIV-XDR-TB in 2021, and percentage change of age-standardized DALY rate were analyzed across GBD regions

Region	HIV-DS-TB		HIV-MDR-TB		HIV-XDR-TB	
	Age-standardized DALY rate (95% UI) 2021	Percentage change of age-standardized DALY rate (95% UI) 1990–2021	Age-standardized DALY rate (95% UI) 2021	Percentage change of age-standardized DALY rate (95% UI) 1990–2021	Age-standardized DALY rate (95% UI) 2021	Percentage change of age-standardized DALY rate (95% UI) 2010–2021
Global	122.54 (96.79–149.60)	−0.04 (−0.26, 0.30)	11.48 (5.13–20.78)	14.05 (7.87–26.04)	0.51 (0.24–0.91)	−0.38 (−0.51, −0.22)
Male	111.04 (88.18–135.54)	−0.03 (−0.23, 0.26)	10.42 (4.75–18.87)	11.78 (6.97–20.99)	0.6 (0.28–1.06)	−0.41 (−0.54, −0.23)
Female	134.52 (106.26–164.14)	−0.04 (−0.29, 0.33)	12.59 (5.59–23.19)	16.74 (8.47–33.67)	0.42 (0.19–0.74)	−0.34 (−0.49, −0.18)
East Asia	8.76 (5.63–12.50)	2.09 (0.59–13.88)	0.84 (0.14–2.44)	3.32 (−0.46, 69.84)	0.16 (0.02–0.52)	−0.03 (−0.75, 1.59)
Southeast Asia	64.34 (51.56–78.18)	2.39 (1.81–3.12)	4.10 (1.55–8.22)	17.01 (4.32–69.27)	0.78 (0.27–1.76)	−0.46 (−0.73, −0.05)
Oceania	140.37 (103.32–199.04)	30.61 (19.97–42.90)	12.07 (2.70–30.59)	1413.68 (350.23–5120.04)	2.28 (0.48–5.79)	1.93 (−0.34, 9.07)
Central Asia	5.12 (2.83–8.54)	−0.61 (−0.74, −0.46)	2.84 (1.36–4.57)	62.01 (19.51–195.64)	1.30 (0.6–2.27)	−0.42 (−0.58, −0.23)
Central Europe	3.41 (2.35–4.91)	−0.61 (−0.66, −0.51)	0.16 (0.05–0.37)	0.01 (−0.71, 2.79)	0.07 (0.02–0.18)	−0.25 (−0.75, 1.17)
Eastern Europe	15.47 (7.74–28.1)	−0.25 (−0.57, 0.12)	12.38 (6.39–20.6)	18.08 (6.85–51.79)	5.67 (2.81–9.34)	−0.42 (−0.59, −0.15)
High-income Asia Pacific	2.05 (1.68–2.32)	0.56 (0.29–0.80)	0.05 (0.01–0.15)	1.67 (−0.36, 9.34)	0.01 (0.00–0.04)	0.00 (−0.66, 1.78)
Australasia	0.62 (0.38–0.97)	−0.91 (−0.92, −0.89)	0.05 (0.01–0.13)	−0.45 (−0.86, 1.50)	0.01 (0.00–0.04)	0.57 (−0.56, 4.20)
Western Europe	3.63 (2.39–5.42)	−0.90 (−0.91, −0.88)	0.18 (0.07–0.4)	−0.71 (−0.86, −0.41)	0.05 (0.02–0.10)	−0.13 (−0.45, 0.38)
Southern Latin America	51.69 (34.31–65.19)	0.40 (−0.05, 0.77)	1.55 (0.30–5.19)	4.22 (−0.04, 24.95)	0.41 (0.08–1.42)	−0.04 (−0.71, 1.38)
High-income North America	1.66 (1.09–2.52)	−0.92 (−0.93, −0.90)	0.06 (0.02–0.17)	−0.96 (−0.99, −0.88)	0.02 (0.00–0.04)	0.23 (−0.49, 1.87)
Caribbean	89.57 (49.97–216.73)	−0.69 (−0.77, −0.51)	0.98 (0.20–3.16)	−0.53 (−0.90, 1.08)	0.17 (0.03–0.58)	0.05 (−0.68, 2.14)
Andean Latin America	37.60 (23.31–59.52)	0.24 (−0.22, 0.94)	6.59 (2.13–14.58)	10.2 (2.16–41.48)	1.10 (0.33–2.61)	−0.27 (−0.61, 0.29)
Central Latin America	30.21 (19.9–43.65)	−0.53 (−0.62, −0.40)	2.17 (0.66–4.62)	10.13 (3.05–28.52)	0.36 (0.11–0.84)	0.17 (−0.39, 1.11)
Tropical Latin America	47.93 (30.18–72.05)	−0.56 (−0.67, −0.45)	3.52 (0.61–10.86)	27.34 (2.95–273.5)	0.59 (0.09–2.00)	0.34 (−0.63, 2.47)
North Africa and Middle East	6.44 (4.49–8.94)	0.43 (−0.05, 1.23)	0.37 (0.13–0.78)	15.41 (5.13–45.72)	0.03 (0.01–0.06)	0.03 (−0.46, 0.77)
South Asia	27.38 (18.7–36.67)	27.74 (14.56–50.68)	4.5 (1.10–10.59)	2653.53 (373.49–33,653.19)	0.23 (0.05–0.58)	−0.57 (−0.86, −0.02)
Central sub-Saharan Africa	461.74 (346.48–600.28)	−0.57 (−0.71, −0.31)	26.72 (8.06–64.04)	2.54 (−0.23, 19.13)	0.36 (0.10–1.01)	−0.48 (−0.80, 0.45)
Eastern sub-Saharan Africa	989.91 (761.62–1210.95)	−0.58 (−0.72, −0.37)	90.59 (37.58–182.99)	25.33 (8.32–79.72)	1.24 (0.49–2.60)	−0.21 (−0.54, 0.39)
Southern sub-Saharan Africa	3400.80 (2869.16–3753.34)	1.55 (0.81–2.49)	304.42 (114.45–658.52)	30.77 (6.52–184.92)	4.17 (1.46–10.03)	−0.44 (−0.76, 0.30)
Western sub-Saharan Africa	386.45 (264.71–552.51)	−0.21 (−0.43, 0.17)	31.97 (10.19–73.71)	3.77 (0.67–15.54)	0.44 (0.13–0.98)	−0.4 (−0.69, 0.11)
High SDI	2.42 (1.63–3.56)	−0.87 (−0.89, −0.85)	0.13 (0.06–0.28)	−0.81 (−0.90, −0.65)	0.03 (0.01–0.07)	−0.25 (−0.46, 0.06)
High-middle SDI	9.86 (6.37–14.22)	−0.19 (−0.31, −0.06)	2.35 (1.21–3.81)	8.02 (3.68–18.58)	0.98 (0.50–1.63)	−0.42 (−0.58, −0.19)
Middle SDI	113.35 (91.86–130.79)	3.19 (2.59–3.83)	9.29 (3.29–20.22)	31.28 (11.27–88.25)	0.47 (0.17–0.96)	−0.29 (−0.55, 0.10)
Low-middle SDI	152.62 (114.05–198.07)	0.00 (−0.30, 0.45)	14.40 (6.00–27.46)	20.54 (6.17–66.69)	0.45 (0.18–0.83)	−0.50 (−0.68, −0.24)
Low SDI	395.23 (310.15–475.43)	−0.58 (−0.70, −0.39)	37.26 (15.52–72.41)	8.29 (2.95–24.32)	0.61 (0.24–1.22)	−0.24 (−0.52, 0.18)

Globally, the World Health Organization began to recommend the XDR-TB surveillance. Consequently, the age-standardized DALY rate of HIV-XDR-TB has been tracked and reported since 1991. However, the GBD 2021 database provides total percentage change data for the periods 1990–2000, 2000–2021, 1990–2021, 2010–2021, and 2019–2021. Therefore, percentage change of age-standardized DALY rates for HIV-XDR-TB spanning 2010–2021 were used in the study

*DALYs* disability-adjusted life years, *GBD* Global Burden of Disease, *HIV-DS-TB* HIV-infected drug-susceptible tuberculosis, *HIV-MDR-TB* HIV-infected multidrug-resistant tuberculosis without extensive drug resistance, *HIV-XDR-TB* HIV-infected extensively drug-resistant tuberculosis, *SDI* Sociodemographic Index, *UI* uncertainty intervals

In 2021, the DALY numbers for HIV-DS-TB, HIV-MDR-TB, and HIV-XDR-TB were 9,910,866 individuals (95% UI: 7,825,966–12,110,494 individuals), 925,471 (95% UI: 413,530–1,668,293 individuals), and 42,095 individuals (95% UI: 19,698–74,093 individuals), respectively (Additional file 1: Table S5).

In 2021, the age-standardized DALY rates for HIV-DS-TB, HIV-MDR-TB, and HIV-XDR-TB showed no significant differences between females and males (both *P* > 0.05; Table 4). Compared to 1990, the age-standardized DALY rate for HIV-DS-TB in 2021 did not exhibit significant changes in either gender. However, the DALY rate for HIV-MDR-TB showed a significant upward trend in both genders (both *P* < 0.05; Table 4).

In 2021, the highest age-standardized DALY rates for HIV-DS-TB and HIV-MDR-TB were recorded in the low SDI region, while the lowest rates were observed in the high SDI region. For HIV-XDR-TB, the highest age-standardized DALY rate was found in the high-middle SDI region, with the lowest in the high SDI region (Table 4). From 1990 to 2021, the age-standardized DALY rates for HIV-DS-TB and HIV-MDR-TB initially increased across the five SDI regions but began to decline thereafter. In contrast, the age-standardized DALY rate for HIV-XDR-TB has shown a very slow decline in recent years (Additional file 1: Fig. S4 A–C).

In 2021, the highest age-standardized DALY rates for HIV-DS-TB and HIV-MDR-TB were found in Southern sub-Saharan Africa, while Eastern Europe had the highest rate for HIV-XDR-TB. In 2021, compared to 1990, regions with increased age-standardized DALY rates for HIV-DS-TB included Oceania, South Asia, East Asia, Southeast Asia, Southern sub-Saharan Africa, and high-income Asia Pacific (all *P* < 0.05. Table 4). Conversely, Western Europe and high-income North America saw decreases in the age-standardized DALY rates for HIV-DS-TB in 2021 (both *P* < 0.05. Table 4). The age-standardized DALY rate for HIV-MDR-TB increased in most of the 21 regions, with the largest increases observed in South Asia, Oceania, and Central Asia (all *P* < 0.05. Table 4). Western Europe and high-income North America were the only regions where the age-standardized DALY rate for HIV-MDR-TB decreased (both *P* < 0.05. Table 4).

In 2021, Lesotho had the highest age-standardized DALY rate for HIV-DS-TB, while Eswatini had the highest rates for both HIV-MDR-TB and HIV-XDR-TB. Compared to 1990, Pakistan showed the largest increases in age-standardized DALY rates for both HIV-DS-TB and HIV-MDR-TB in 2021 (both *P* < 0.05; Additional file 1: Table S2).

### Age and gender distribution

In 2021, the age-specific incidence rate of HIV-DS-TB was higher in females than in males for the age groups 15–19, 20–24, 25–29, and 31–34 years (all *P* < 0.05), with no significant differences in other age groups (all *P* > 0.05). Similarly, the incidence rate of HIV-MDR-TB was higher in females than in males in the 20–24 age group (*P* < 0.05), with no significant differences in other age groups (all *P* > 0.05). No significant differences in the incidence rate of HIV-XDR-TB were observed between males and females across all age groups (all *P* > 0.05. Additional file 1: Fig. S5 A–C).

In 2021, the age-specific prevalence rate of HIV-DS-TB was higher among females than males in the age groups 15–19, 20–24, 25–29, and 31–34 years, with no significant differences in other age groups (all *P* > 0.05). For HIV-MDR-TB, the prevalence rate was higher in females than males in the 20–24 age group, with no significant differences in other age groups (all *P* > 0.05). The prevalence rate of HIV-XDR-TB showed no significant differences between males and females across all age groups (all *P* > 0.05. Additional file 1: Fig. S6 A–C).

In 2021, the age-specific mortality rate of HIV-DS-TB was higher in females than males in the 20–24 and 25–30 age groups (all P < 0.05), with no significant differences in other age groups (all *P* > 0.05). There were no significant differences in the age-specific mortality rates of HIV-MDR-TB and HIV-XDR-TB between males and females across all age groups (all *P* > 0.05. Additional file 1: Fig. S7 A–C).

In 2021, the age-specific DALY rate for HIV-DS-TB was higher in females than males in the 15–19, 20–24, and 25–29 age groups (all *P* < 0.05), with no significant differences in other age groups (all *P* > 0.05). The age-specific DALY rates for HIV-MDR-TB and HIV-XDR-TB showed no significant differences between males and females across all age groups (all *P* > 0.05. Additional file 1: Fig. S8 A–C).

### Association between ASRs and SDI

In 2021, across 204 countries and territories, the ASIR (*r* = −0.707, *P* < 0.001), ASPR (*r* = −0.720, *P* < 0.001), ASMR (*r* = −0.702, *P* < 0.001), and age-standardized DALY rate (*r* = −0.717, *P* < 0.001) of HIV-DS-TB all demonstrated strong inverse relationship with SDI (Additional file 1: Fig. S9 A–D). Similarly, for HIV-MDR-TB, negative correlations were observed between SDI and ASIR (*r* = −0.644, *P* < 0.001), ASPR (*r* = −0.679, *P* < 0.001), ASMR (*r* = −0.668, *P* < 0.001), and the age-standardized DALY rate (*r* = −0.675, *P* < 0.001. Additional file 1: Fig. S10 A–D). For HIV-XDR-TB, the negative correlations with SDI were also significant across ASIR (*r* = −0.489, *P* < 0.001), ASPR (*r* = −0.503, *P* < 0.001), ASMR (*r* = −0.466, *P* < 0.001), and the age-standardized DALY rate (*r* = −0.468, *P* < 0.001. Additional file 1: Fig.S11 A–D).

From 1990 to 2021, the ASIR (*r* = −0.731, *P* < 0.001), ASPR (*r* = −0.755, *P* < 0.001), ASMR (*r* = −0.697, *P* < 0.001), and age-standardized DALY rate (*r* = −0.698, *P* < 0.001) for HIV-DS-TB exhibited significant negative correlations with the SDI. However, these trends were not uniform across all regions. In Southern, Eastern, and Western sub-Saharan Africa, these metrics initially increased rapidly with rising SDI, reached a peak, and then declined sharply with further increases in SDI (Additional file 1: Fig. S12A–D). For HIV-MDR-TB, similar negative correlations were observed between SDI and ASIR (*r* = −0.573, *P* < 0.001), ASPR (*r* = −0.611, *P* < 0.001), ASMR (*r* = −0.572, *P* < 0.001), and the age-standardized DALY rate (*r* = −0.572, *P* < 0.001). In Southern, Eastern, and Western sub-Saharan Africa, these indicators initially rose rapidly with increasing SDI, peaked, and then declined sharply (Additional file 1: Fig. S13 A–D). Furthermore, when the SDI is below 0.75, the ASIR, ASPR, ASMR, and age-standardized DALY rate for HIV-XDR-TB increase gradually with rising SDI. However, once the SDI surpasses 0.80, these indicators decline rapidly with further increases in SDI (Additional file 1: Fig.S14 A–D).

### Risk factors for ASMR and age-standardized DALY rate

Globally, the primary risk factors for the ASMR and age-standardized DALY rate of HIV-DS-TB from 1990 to 2021 were (in descending order): unsafe sex, drug use, and intimate partner violence. In global, middle SDI, and low-middle SDI regions, the contribution of unsafe sex to the ASMR and age-standardized DALY rate of HIV-DS-TB has been increasing. In high SDI regions, drug use has increasingly contributed to the ASMR and age-standardized DALY rate of HIV-DS-TB (Additional file 1: Fig. S15 A–B).

For HIV-MDR-TB, the contribution of drug use to the ASMR and age-standardized DALY rate initially increased and then declined in high-middle and high SDI regions from 1990 to 2021. However, unsafe sex remained the largest contributor to the ASMR and age-standardized DALY rate throughout this period (Additional file 1: Fig. S15 C–D).

The primary risk factors for the ASMR and age-standardized DALY rate of HIV-XDR-TB were (in descending order): unsafe sex, drug use, and intimate partner violence. However, the contributions of these factors varied by region. In high-middle SDI regions, drug use surpassed unsafe sex as the leading contributor to the ASMR and age-standardized DALY rate. In low SDI regions, intimate partner violence contributed more to the ASMR and age-standardized DALY rate than drug use (Additional file 1: Fig. S15 E–F).

### Projecting disease burden

**Fig. 1:**
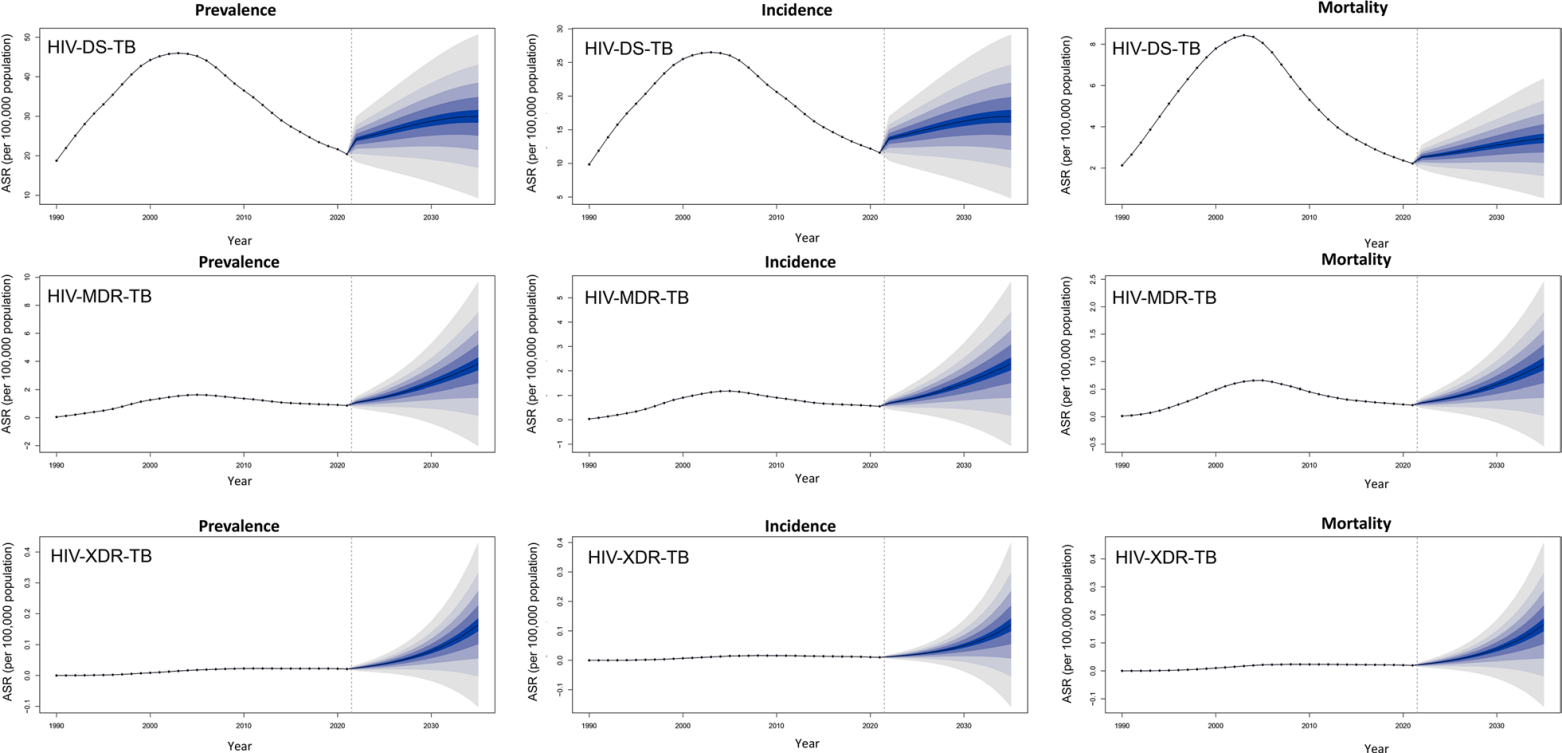
The ASR of HIV-DS-TB, HIV-MDR-TB, and HIV-XDR-TB was assessed globally from 1990 to 2021, with forecasted ASR values projected for 2022 to 2035 *ASR* age-standardized incidence rate, *HIV-DS-TB* HIV-infected drug-susceptible tuberculosis, *HIV-MDR-TB* HIV-infected multidrug-resistant tuberculosis without extensive drug resistance, *HIV-XDR-TB* HIV-infected extensively drug-resistant tuberculosis

The study projects the ASR and EAPC for HIV-DS-TB, HIV-MDR-TB, and HIV-XDR-TB from 2022 to 2035. It highlights that the ASIR, ASPR, and ASMR for HIV-DS-TB, HIV-MDR-TB, and HIV-XDR-TB are expected to show an increasing trend globally (Table 5. Figure 1. Additional file 1: Table S6).

**Table 5:** Predicted ASR (per 100,000 population) of HIV-DS-TB, HIV-MDR-TB, and HIV-XDR-TB spanning 2022–2035, based on the Bayesian Age-Period-Cohort Model

Index	HIV-DS-TB		HIV-MDR-TB		HIV-XDR-TB	
	ASR (per 100,000 population) (95% *CI*) 2035	EAPC (95% *CI*) 2022–2035	ASR (per 100,000 population) (95% *CI*) 035	EAPC (95% *CI*) 2022–2035	ASR (per 100,000 population) (95% *CI*) 2035	EAPC (95% *CI*) 2022–2035
Incidence	17.00 (2.55–31.45)	1.75 (1.54–1.97)	2.28 (0.00–6.28)	9.78 (9.61–9.96)	0.16 (0.00–0.50)	16.18 (16.12–16.23)
Prevalence	29.98 (5.40–54.57)	1.75 (1.53–1.96)	3.82 (0.00–10.76)	10.24 (10.05–10.44)	0.16 (0.00–0.47)	15.76 (15.74–15.79)
Death	3.44 (0.00–6.88)	2.50 (2.40–2.60)	0.96 (0.00–2.73)	10.84 (10.76–10.91)	0.12 (0.00–0.43)	18.99 (18.95–19.03)

Globally, the World Health Organization began to recommend XDR-TB surveillance in 1991. Consequently, the age-standardized incidence rate and prevalence rate of HIV-XDR-TB have been tracked and reported since 1991, and the age-standardized mortality rate has been tracked and reported since 1993. When the ASRs is predicted for a given year, if the lower limits of the 95% *CI*s is below 0, 0 is set

*ASR* age-standardized rate, *CI* Confidence interval, *EAPC* estimated annual percentage change, *HIV-DS-TB* HIV-infected drug-susceptible tuberculosis, *HIV-MDR-TB* HIV-infected. multidrug-resistant tuberculosis without extensive drug resistance, *HIV-XDR-TB* HIV-infected extensively drug-resistant tuberculosis

## Discussion

The study presents the global burden of HIV-TB co-infection based on data from the GBD 2021 database. The findings indicate that the decline in the ASIR for HIV-MDR-TB and HIV-XDR-TB is notably slow in middle-income and low-income countries, underscoring the ongoing severity of HIV-TB as a global public health issue. Furthermore, HIV-TB remains a significant and unresolved threat in sub-Saharan Africa and Asia. The study provides essential insights for policymakers and health administrators to develop targeted measures for HIV-TB prevention and control.

### Early screening essential for controlling HIV-TB co-infection transmission

The study found that the ASIR of HIV-XDR-TB is decreasing in low-income regions but increasing in middle-income and high-middle-income regions. This discrepancy is attributed to the low disease detection capabilities in resource-limited areas. Early detection of infection sources and timely implementation of preventive measures are essential to interrupting the transmission of infectious diseases. HIV-TB co-infection is a significant public health concern, as the diseases exacerbate each other, increasing the risk of morbidity and mortality. Early detection and treatment of HIV-TB co-infected individuals are therefore crucial for controlling both TB and HIV/AIDS epidemics [5, 6].

World Health Organization recommends bidirectional screening as the primary strategy for identifying HIV-TB co-infected patients. Regardless of the TB prevalence and HIV infection levels in a country or region, all TB patients should undergo HIV antibody testing, and all HIV-positive individuals should be screened for active TB. This approach aims to strengthen the prevention and control of these two severe chronic infectious diseases simultaneously [13]. Studies have shown that the proportion of TB diagnoses among newly identified PLWH is significantly higher than among previously known HIV-positive patients [5, 6]. The finding underscores the necessity of comprehensive TB screening, particularly for newly diagnosed HIV-positive individuals.

Previously, TB screening among PLWH primarily relied on symptomatic indicators of TB, with traditional methods such as acid-fast bacilli smear microscopy, TB culture, and immunological tests (e.g., tuberculin skin test, interferon-γ release assay (IGRA)) playing a crucial role, especially in resource-limited settings [14]. However, advances in diagnostic technologies have facilitated earlier TB diagnosis in HIV-positive individuals. New methods and technologies, such as the Gene Xpert MTB/RIF molecular diagnostic technique, offer advantages like shorter detection times and simpler operation, reducing TB diagnosis time from weeks to mere hours. Next-generation sequencing provides rapid diagnostic guidance, particularly in cases of rare or multiple pathogen co-infections in PLWH [14, 15]. In addition, certain biomarkers have demonstrated diagnostic value for HIV-TB co-infection [16]. Recently, multi-omics approaches have also provided new avenues for the early diagnosis of TB in PLWH [17]. In summary, future efforts should focus on enhancing the proactive detection of HIV-TB co-infected individuals through highly sensitive and specific screening methods and continually optimizing screening strategies. Achieving and maintaining high rates of TB testing among HIV/AIDS patients and HIV antibody testing among TB patients is essential for effectively controlling the spread of HIV-TB.

### HAART, anti-TB treatment with new drugs, and public health services can significantly reduce the transmission of HIV-TB co-infection

This study found that the ASMR of HIV-DS-TB has declined rapidly in high-income regions but has decreased very slowly in low-income countries. Moreover, the ASMR for HIV-MDR-TB and HIV-XDR-TB is increasing in low-income countries and regions. This disparity is largely attributed to the accessibility of medical resources and the standardized management and treatment of patients in developed countries, in contrast to the limited accessibility of medical resources in low-income countries.

Individuals with advanced HIV/AIDS are particularly susceptible to severe illnesses and death, even after initiating HARRT, and the most common cause of death is pulmonary TB, cryptococcal meningitis, and severe bacterial infections [18]. Due to the long-term immunosuppression experienced by AIDS patients, immune reconstitution is essential to alleviate this suppression. HAART effectively suppresses viral replication, restores the damaged cellular immune function in PLWH, and achieves immune reconstitution. This treatment reduces the occurrence of opportunistic infections, delays disease progression, improves quality of life, and extends the lifespan of patients [18, 19]. In addition, Strengthening the diagnosis of OPIs among PLWH, particularly MDR-TB and XDR-TB, is crucial. Antiretroviral and anti-TB medications should be administered continuously and immediately upon diagnosis, regardless of the environment or patient relocation. Additionally, it is imperative to address factors that hinder continuous treatment, such as stigma and discrimination in healthcare settings, remote facility locations, transportation and opportunity costs, and long waiting times [4].

The study found that the incidence of HIV-TB co-infection was highest among patients aged 15–39 years. This can be attributed to increased social activities, higher exposure, risky behaviors, and greater mobility within this age group. Interestingly, HIV-TB co-infection was more common in females than in males, contrasting with the global trend where TB is more prevalent in males [20]. Additionally, among individuals co-infected with HIV and TB, females had a higher mortality rate than males, consistent with previous studies [21, 22]. This disparity may be due to unsafe sexual practices and intimate partner violence, which are significant factors contributing to the gender differences in HIV burden in high-prevalence countries. These findings highlight the substantial role of gender-specific health risk factors in HIV and TB co-infection and underscore the urgency and importance of targeted prevention and treatment strategies for women in these high-burden regions [23]. Failing to recognize the specific drivers of HIV-TB epidemics in different countries impedes the adaptation of the ‘END TB Strategy’ at the national level. The results of this analysis can inform the design of future studies aimed at identifying country-specific drivers of TB using individual-level data [23].

In sub-Saharan Africa, the estimated HIV incidence among men who have sex with men (MSM) in 2020 was nearly 5 cases per 100 person-years, which is 27 to 150 times higher than that of the general adult male population (aged 15 and above) in the region [24]. Young people from key populations are particularly vulnerable, struggling to maintain health and safety in environments characterized by stigma, discrimination, harassment, punitive laws, and social taboos [6, 25]. In the Asia–Pacific region, HIV infection rates among young MSM have more than doubled in Indonesia (from 6% in 2011 to 13% in 2019), nearly quadrupled in Malaysia (from 6% in 2012 to 15% in 2022), and almost quadrupled in Vietnam (from 3% in 2011 to 11% in 2022) [26]. The increase in new HIV cases, coupled with frequent social activities among adolescents, insufficient HIV and TB diagnostic capacities, and inadequate health services and medical supplies, has led to a rise in HIV-TB co-infection incidence and mortality in Southeast Asian and African countries.

The study found that in low- and middle-income regions such as Africa, the Middle East, and South Asia, the incidence and mortality rates of HIV-DS-TB, HIV-MDR-TB, and HIV-XDR-TB are decreasing very slowly. Several factors contribute to this issue. Poverty, malnutrition, overcrowded living conditions, and inadequate healthcare infrastructure are widespread in these regions, facilitating the spread of TB [27]. Additionally, insufficient monitoring leads to over 50% of newly diagnosed HIV infections being at an advanced AIDS stage. This is driven by stigma and discrimination, side effects of medications, affordability issues, and unreliable healthcare services [28, 29]. Approximately one-quarter of PLWH discontinue HAART within six months of initiation, and over one-fifth of those on HAART do not achieve viral suppression [30]. In low- and lower-middle-income regions, PLWH have significantly lower CD4^+^ T-cell counts post-HAART compared to those in upper-middle and high-income countries, contributing to increased OPIs.

In many regions with a high prevalence of TB and HIV, patients face limited access to quality healthcare, diagnostic services, and effective treatments. This scarcity of medical services leads to delays in diagnosis and treatment, exacerbating the spread of TB, including drug-resistant strains, and increasing patient mortality [31]. Furthermore, in areas with inadequate treatment supervision, the overuse or misuse of TB medications contributes to the emergence of drug-resistant TB strains, accelerating the spread of the disease. Insufficient public health education leads to misunderstandings about TB, delays in seeking medical care, and poor adherence to treatment regimens. These factors accelerate the spread of *Mtb* strains, increasing the incidence and mortality of HIV-TB co-infection [32, 33]. Insufficient public health education leads to misunderstandings about TB, delays in seeking medical care, and poor adherence to treatment regimens. These factors accelerate the *Mtb* transmission, increasing the incidence and mortality of HIV-TB co-infection [32, 33].

### Control strategies and measures derived from the One Health approach can curb HIV-TB transmission

In the new era, preventing and controlling the HIV-TB epidemic necessitates a One Health approach integrating medical, social, economic, and environmental interventions [34–37]. Essential strategies include coordinated care, routine screening, and holistic treatment plans. Strengthening healthcare infrastructure through improved accessibility, capacity building, and efficient supply chain management will enhance care delivery. Public health policies must prioritize comprehensive national strategies, adequate funding, and robust surveillance systems.

In controlling HIV-TB co-infection, community engagement through awareness campaigns, the deployment of community health workers, and stigma reduction programs is essential to enhance public understanding and support. Addressing social determinants of health, such as poverty alleviation, improved living conditions, and increased education and employment opportunities, is crucial. Additionally, research and innovation should focus on vaccine development, new treatment regimens, and implementation science. International collaboration through global partnerships, funding mechanisms, and technical assistance can bolster national and regional efforts [34–38]. This multifaceted approach aims to reduce the incidence and improve outcomes of HIV-TB co-infection.

Several limitations of this study need to be acknowledged. First, the inherent limitations of the GBD 2021 study methodology affect the accuracy and completeness of model estimates. Missing HIV and TB data from some countries and regions significantly impact these estimates. Additionally, variations in data quality, accuracy, and comparability can introduce biases [9, 10]. Data on the incidence, prevalence, mortality, and DALYs of HIV-MDR-TB and HIV-XDR-TB are insufficient in some regions, particularly for HIV-XDR-TB, as surveillance for XDR-TB only began in 1991 in a few countries, with many lacking the capacity for such surveillance, leading to data gaps [4]. Second, the GBD 2021 database relies on model fitting rather than real-world data, potentially resulting in overestimation or underestimation. Third, the rates for HIV-DS-TB, HIV-MDR-TB, and HIV-XDR-TB in 204 countries and territories were calculated based on a globally standardized population to ensure comparability. However, these standardized rates may not accurately represent the true disease burden of HIV-TB co-infection in each country. Fourth, EAPC estimates the average trend over the past three decades without accounting for the uncertainty of these rates. While EAPC is accurate under linear trends, it can be misleading when rates exhibit non-linear trends, such as U-shaped, V-shaped, or L-shaped patterns. Fifth, a comprehensive assessment of the disease burden should consider broader economic, familial, and social impacts. Sixth, the GBD 2021 data lack comprehensive information on overall HIV-TB co-infection, hindering a holistic assessment of the situation. Future studies should employ multidimensional analyses to enhance the accuracy and robustness of the results.

## Conclusions

The findings indicate a critical need for enhanced diagnostic and treatment strategies in low- and middle-income countries where the burden of HIV-TB co-infection remains high. Strengthening healthcare infrastructure, increasing accessibility to quality medical care, and improving public health education are pivotal in combating the dual epidemic. Moreover, the development of new screening technologies and comprehensive management plans tailored to high-burden regions could significantly reduce the incidence and mortality associated with HIV-TB co-infection. Addressing social determinants of health and ensuring sustained political and financial commitment are crucial for achieving long-term control and eventual eradication of HIV and TB.

### Supplementary Information

Supplementary material 1. Contains materials used throughout the study. Table S1: The number of incidence cases of HIV, HIV-DS-TB, HIV-MDR-TB, and HIV-XDR-TB individuals in 2021, and percentage change of the number of incidence case were analyzed across GBD regions. Table S2: Age-standardized rates of HIV-DS-TB, HIV-MDR-TB, and HIV-XDR-TB in 2021, and percentage change of age-standardized rates in 204 countries and territories. Table S3: The number of prevalence cases of HIV, HIV-DS-TB, HIV-MDR-TB, and HIV-XDR-TB individuals in 2021, and percentage change of number of prevalence cases were analyzed across GBD regions. Table S4: The number of death cases of HIV, HIV-DS-TB, HIV-MDR-TB, and HIV-XDR-TB individuals in 2021, and percentage change of number of death cases were analyzed across GBD regions. Table S5: The number of DALY cases of HIV, HIV-DS-TB, HIV-MDR-TB, and HIV-XDR-TB individuals in 2021, and percentage change of the number of DALY cases were analyzed across GBD regions. Table S6: Predicted age-standardized rates of HIV-DS-TB, HIV-MDR-TB, and HIV-XDR-TB spanning 2022–2035, based on the Bayesian Age-Period-Cohort Model. Fig. S1: The trends in the age-standardized incidence rate for HIV-DS-TB, HIV-MDR-TB, HIV-XDR-TB varied across the five SDI regions. Fig. S2: The trends in the age-standardized prevalence rate for HIV-DS-TB, HIV-MDR-TB, HIV-XDR-TB varied across the five SDI regions. Fig. S3: The trends in the age-standardized mortality rate for HIV-DS-TB, HIV-MDR-TB, HIV-XDR-TB varied across the five SDI regions. Fig. S4: The trends in the age-standardized DALY rates for HIV-DS-TB, HIV-MDR-TB, HIV-XDR-TB varied across the five SDI regions. Fig. S5: The specific incidence rate of HIV-DS-TB, HIV-MDR-TB, and HIV-XDR-TB showed notable differences across age and gender distributions in 2021. Fig. S6: The specific prevalence rate of HIV-DS-TB, HIV-MDR-TB, and HIV-XDR-TB showed notable differences across age and gender distributions in 2021. Fig. S7: The specific mortality rate of HIV-DS-TB, HIV-MDR-TB, and HIV-XDR-TB showed notable differences across age and gender distributions in 2021. Fig. S8: The specific age-standardized DALY rate of HIV-DS-TB, HIV-MDR-TB, and HIV-XDR-TB showed notable differences across age and gender distributions in 2021. Fig. S9: The association between the SDI and the age-standardized incidence rate, mortality rate, and DALY rate of HIV-DS-TB across 204 countries and regions in 2021. Fig. S10: The association between the SDI and the age-standardized incidence rate, death rate, and DALY rate of HIV-MDR-TB across 204 countries and regions in 2021. Fig. S11: The association between the SDI and the age-standardized incidence rate, death rate, and DALY rate of HIV-XDR-TB across 204 countries and regions in 2021. Fig.S12: The association between the age-standardized incidence rate, prevalence rate, mortality rate, and DALY rate of HIV-DS-TB with the SDI from 1990 to 2021. Fig. S13: The association between the age-standardized incidence rate, prevalence rate, mortality rate, and DALY rate of HIV-MDR-TB with the SDI from 1990 to 2021. Fig. S14: The association between the age-standardized incidence rate, prevalence rate, mortality rate, and DALY rate of HIV-XDR-TB with the SDI from 1990 to 2021. Fig. S15: The association between risk factors and the age-standardized mortality rate, age-standardized DALY rate of HIV-DS-TB, HIV-MDR-TB, HIV-XDR-TB from 1990 to 2021.

## Data Availability

The datasets analysed during the current study are available at http://ghdx.healthdata.org/gbd-results-tool.

## Abbreviations

AIDS: Acquired immune deficiency syndrome
ASMR: Age-standardized mortality rate
ASIR: Age-standardized incidence rate
ASPR: Age-standardized prevalence rate
ASRs: Age-standardized rate
BAPC: Bayesian age-period-cohort
*CI*: Confidence interval
DALYs: Disability-adjusted life years
DisMoD-MR: Disease-model-Bayesian meta-regression
DS-TB: Drug-susceptible tuberculosis
EAPC: Annual percentage changes
GATHER: Guidelines for Accurate and Transparent Health Estimates Reporting
GBD: Global Burden of Disease
HARRT: Highly active anti-retroviral therapy
HIV: Human immunodeficiency virus
HIV-DS-TB: HIV-infected drug-susceptible tuberculosis
HIV-MDR-TB: HIV-infected multidrug-resistant tuberculosis without extensive drug resistance
HIV-XDR-TB: HIV-infected extensively drug-resistant tuberculosis
IHME: Institute for Health Metrics and Evaluation
ICD: International Classification of Diseases
IGRA: Interferon-γ release assay
MDR-TB: Multidrug-resistant tuberculosis without extensive drug resistance
MR-BRT: Bayesian priors, regularisation, and trimming
MSM: Men who have sex with men
*Mtb*: *Mycobacterium tuberculosis*
OPIs: Opportunistic infections
PLWH: Person living with HIV
SDI: Sociodemographic Index
TB: Tuberculosis
UI: Uncertainty interval
