# Supplementary material for: Epidemiological features and temporal trends of the co-infection between HIV and tuberculosis, 1990–2021: findings from the Global Burden of Disease Study 2021
Source: Infect Dis Poverty. 2024 Aug 16;13:59. doi: 10.1186/s40249-024-01230-3 (PMC11328430; doi:10.1186/s40249-024-01230-3)
Supplement: Supplementary file 1 — Supplementary material 1. Contains materials used throughout the study. Table S1: The number of incidence cases of HIV, HIV-DS-TB, HIV-MDR-TB, and HIV-XDR-TB individuals in 2021, and percentage change of the number of incidence case were analyzed across GBD regions. Table S2: Age-standardized rates of HIV-DS-TB, HIV-MDR-TB, and HIV-XDR-TB in 2021, and percentage change of age-standardized rates in 204 countries and territories. Table S3: The number of prevalence cases of HIV, HIV-DS-TB, HIV-MDR-TB, and HIV-XDR-TB individuals in 2021, and percentage change of number of prevalence cases were analyzed across GBD regions. Table S4: The number of death cases of HIV, HIV-DS-TB, HIV-MDR-TB, and HIV-XDR-TB individuals in 2021, and percentage change of number of death cases were analyzed across GBD regions. Table S5: The number of DALY cases of HIV, HIV-DS-TB, HIV-MDR-TB, and HIV-XDR-TB individuals in 2021, and percentage change of the number of DALY cases were analyzed across GBD regions. Table S6: Predicted age-standardized rates of HIV-DS-TB, HIV-MDR-TB, and HIV-XDR-TB spanning 2022–2035, based on the Bayesian Age-Period-Cohort Model. Fig. S1: The trends in the age-standardized incidence rate for HIV-DS-TB, HIV-MDR-TB, HIV-XDR-TB varied across the five SDI regions. Fig. S2: The trends in the age-standardized prevalence rate for HIV-DS-TB, HIV-MDR-TB, HIV-XDR-TB varied across the five SDI regions. Fig. S3: The trends in the age-standardized mortality rate for HIV-DS-TB, HIV-MDR-TB, HIV-XDR-TB varied across the five SDI regions. Fig. S4: The trends in the age-standardized DALY rates for HIV-DS-TB, HIV-MDR-TB, HIV-XDR-TB varied across the five SDI regions. Fig. S5: The specific incidence rate of HIV-DS-TB, HIV-MDR-TB, and HIV-XDR-TB showed notable differences across age and gender distributions in 2021. Fig. S6: The specific prevalence rate of HIV-DS-TB, HIV-MDR-TB, and HIV-XDR-TB showed notable differences across age and gender distributions in 2021. Fig. S7: The specific m [file 40249_2024_1230_MOESM1_ESM.docx]

**Additional file1**

**Epidemiological features and temporal trends of the co-infection between HIV and Tuberculosis, 1990–2021: findings from the Global Burden of Disease Study 2021**

Shun-Xian Zhang^1,2†^, Ji-Chun Wang^3†^, Jian Yang^3^, Shan Lv^2,4^, Lei Duan^2,4^, Yan Lu^2,4^, Li-Guang Tian^2,4^, Mu-Xin Chen^2,4^, Qin Liu^2,4^, Fan-Na Wei^2,4^, Xin-Yu Feng^4^, Guo-Bing Yang^5^, Yong-Jun Li^5^, Yu Wang^1^, Xiao-Jie Hu^1^, Ming Yang^1^, Zhen-Hui Lu^1^, Shao-Yan Zhang^1^, Shi-Zhu Li^2,4*^, Jin-Xin Zheng^4*^

^1^ Longhua Hospital, Shanghai University of Traditional Chinese Medicine, Shanghai 200032, China.

^2^ National Institute of Parasitic Diseases at Chinese Center for Disease Control and Prevention (Chinese Center for Tropical Diseases Research); NHC Key Laboratory of Parasite and Vector Biology; WHO Collaborating Centre for Tropical Diseases; National Center for International Research on Tropical Diseases; National Key Laboratory of Intelligent Tracking and Forecasting for Infectious Diseases, Shanghai 200025, China.

^3^ Department of Science and Technology, Chinese Center for Disease Control and Prevention; National Key Laboratory of Intelligent Tracking and Forecasting for Infectious Diseases, Beijing 102206, China.

^4^ School of Global Health, Chinese Center for Tropical Diseases Research-Shanghai Jiao Tong University School of Medicine, Shanghai 200025, China.

^5^ Gansu Provincial Center for Disease Control and Prevention, Lanzhou 730000, China.

^†^ Shun-Xian Zhang and Ji-Chun Wang contributed equally to this work.

*Correspondence : Shi-Zhu Li, lisz@nipd.chinacdc.cn

Jin-Xin Zheng, jamesjin63@163.com

**Items**

1 Search method for data from GBD 2021 Datebase.

2 Case definition for HIV-DS-TB, HIV-MDR-TB, and HIV-XDR-TB

3 Risk factors were categorized under Level 2 for HIV-DS-TB, HIV-MDR-TB, and HIV-XDR-TB.

4 Table S1: The number of incidence cases of HIV, HIV-DS-TB, HIV-MDR-TB, and HIV-XDR-TB individuals in 2021, and percentage change of the number of incidence case were analyzed across GBD regions.

5 Table S2: Age-standardized rates of HIV-DS-TB, HIV-MDR-TB, and HIV-XDR-TB in 2021 year, and percentage change of age-standardized rates in 204 countries and territories.

6 Table S3: The number of prevalence cases of HIV, HIV-DS-TB, HIV-MDR-TB, and HIV-XDR-TB individuals in 2021, and percentage change of number of prevalence cases were analyzed across GBD regions.

7 Table S4: The number of death cases of HIV, HIV-DS-TB, HIV-MDR-TB, and HIV-XDR-TB individuals in 2021, and percentage change of number of death cases were analyzed across GBD regions.

8 Table S5: The number of DALY cases of HIV, HIV-DS-TB, HIV-MDR-TB, and HIV-XDR-TB individuals in 2021, and percentage change of the number of DALY cases were analyzed across GBD regions.

9 Table S6: Predicted age-standardized rates of HIV-DS-TB, HIV-MDR-TB, and HIV-XDR-TB spanning 2022－2035, based on the Bayesian Age-Period-Cohort Model.

10 Fig. S1: The trends in the age-standardized incidence rate for HIV-DS-TB, HIV-MDR-TB, HIV-XDR-TB varied across the five SDI regions.

11 Fig. S2: The trends in the age-standardized prevalence rate for HIV-DS-TB, HIV-MDR-TB, HIV-XDR-TB varied across the five SDI regions.

12 Fig. S3: The trends in the age-standardized mortality rate for HIV-DS-TB, HIV-MDR-TB, HIV-XDR-TB varied across the five SDI regions.

13 Fig. S4: The trends in the age-standardized DALY rates for HIV-DS-TB, HIV-MDR-TB, HIV-XDR-TB varied across the five SDI regions

14 Fig. S5: The specific incidence rate of HIV-DS-TB, HIV-MDR-TB, and HIV-XDR-TB showed notable differences across age and gender distributions in 2021 year.

15 Fig. S6: The specific prevalence rate of HIV-DS-TB, HIV-MDR-TB, and HIV-XDR-TB showed notable differences across age and gender distributions in 2021 year.

16 Fig. S7: The specific mortality rate of HIV-DS-TB, HIV-MDR-TB, and HIV-XDR-TB showed notable differences across age and gender distributions in 2021 year.

17 Fig. S8: The specific age-standardized DALY rate of HIV-DS-TB, HIV-MDR-TB, and HIV-XDR-TB showed notable differences across age and gender distributions in 2021 year.

18 Fig. S9: The association between the SDI and the age-standardized incidence rate, mortality rate, and DALY rate of HIV-DS-TB across 204 countries and regions in 2021 year.

19 Fig. S10: The association between the SDI and the age-standardized incidence rate, death rate, and DALY rate of HIV-MDR-TB across 204 countries and regions in 2021 year.

20 Fig. S11: The association between the SDI and the age-standardized incidence rate, death rate, and DALY rate of HIV-XDR-TB across 204 countries and regions in 2021 year.

21 Fig. S12: The association between the age-standardized incidence rate, prevalence rate, mortality rate, and DALY rate of HIV-DS-TB with the SDI from 1990 to 2021 year.

22 Fig. S13: The association between the age-standardized incidence rate, prevalence rate, mortality rate, and DALY rate of HIV-MDR-TB with the SDI from 1990 to 2021 year.

23 Fig. S14: The association between the age-standardized incidence rate, prevalence rate, mortality rate, and DALY rate of HIV-XDR-TB with the SDI from 1990 to 2021 year.

24 Fig. S15: The association between risk factors and the age-standardized mortality rate, age-standardized DALY rate of HIV-DS-TB, HIV-MDR-TB, HIV-XDR-TB from 1990 to 2021 year.

**Search method for data from GBD 2021 Datebase.**

Data for this study were obtained from the Institute for Health Metrics and Evaluation (IHME) by accessing their official website (https://ghdx.healthdata.org/gbd-results) via a web browser. The specific search criteria in the "Search" interface were as follows: GBD Estimate (Cases of death or injury, risk factor), Measure (Incidence, Deaths, Prevalence, DALYs), Metric (Number, Percent, Rate), Cause (HIV/AIDS-Drug-susceptible Tuberculosis, HIV/AIDS Multidrug-resistant Tuberculosis without extensive drug resistance, HIV/AIDS-Extensively drug-resistant Tuberculosis), Location (Global, All countries and regions, Different SDI regions, 204 countries and territories), Age (All ages, Age-standardized, <5 years, 5–9 years, 10–14 years, 15–19 years, 20–24 years, 25–29 years, 30–34 years, 35–39 years, 40–44 years, 45–49 years, 50–54 years, 55–59 years, 60–64 years, 65–69 years, 70–74 years, 75－79 years, 80–84 years, 85–89 years, 90–94 years, 95+ years), Sex (Both, male, female), Year(1990–2021, 2010–2021, and each year from 1990 to 2021). As XDR-TB diagnosis and confirmation were only recommended by WHO post-1991, HIV-XDR-TB data became available in 1991 from GBD 2021 database, including incidence, prevalence, and DALY numbers and rates. From 1993 onwards, data on the number cases and rate of mortality also became available. Hence, for data pertaining to HIV-XDR-TB, it was records from either 1991 or 1993 onward .

**Case definition for HIV-DS-TB, HIV-MDR-TB, and HIV-XDR-TB**

Infection with the HIV causes influenza-like symptoms during the acute period following infection and can lead to AIDS if untreated. HIV attacks the immune system of its host, leaving infected individuals more susceptible to opportunistic infections like tuberculosis. Although there are two different subtypes of HIV, HIV-1 and HIV-2, no distinction is made in our estimation process or presentation of results. For HIV, International Classification of Diseases (ICD)-10 codes are B20-B24, C46-C469, D84.9; ICD-9 codes are 042–044, 112–118 (after 1980), 130 (after 1980), 136.3–136.8 (after 1980), 176.0–176.9 (after 1980), 279 (after 1980); and ICD–9 BTL codes are B184–B185.

TB is an infectious disease caused by Mtb. The case definition includes all forms of TB, including pulmonary TB and extrapulmonary TB, which are bacteriologically confirmed or clinically diagnosed. For TB, the ICD–10 codes are A10-A19.9, B90-B90.9, K67.3, K93.0, M49.0, P37.0, and ICD-9 codes are 010–019.9, 137–137.9, 138.0, 138.9, 139.9, 320.4, 730.4–730.6. For HIV-TB, the ICD-10 code is B20.0. For HIV-TB, the ICD-10 codes is B20.0

**Risk factors were categorized under Level 2 for HIV-DS-TB, HIV-MDR-TB, and HIV-XDR-TB.**

GBD 2021 estimated mortality and DALYs for various risk factors and combinations of risk factors across different geographical units, including countries [16, 17][9, 10]. In the study, data on ASMR and age standardized DALY rate due to risk factors were categorized under Level 2, including air pollution, alcohol use, child and maternal malnutrition, childhood sexual abuse and bullying, dietary risks, drug use, high body-mass index, high fasting plasma glucose, high low density lipoprotein cholesterol, high systolic blood pressure, intimate partner violence, kidney disfunction, low bone mineral density, low physical activity, non-optimal temperature, occupational risks, other environmental risks, tobacco, unsafe sex, unsafe water, sanitation and hand washing

Table S1 The number of incidence cases of HIV, HIV-DS-TB, HIV-MDR-TB, and HIV-XDR-TB individuals in 2021, and percentage change of the number of incidence case were analyzed across GBD regions.

| Region | HIV/AIDS | HIV/AIDS | HIV-DS-TB | HIV-DS-TB | HIV-MDR-TB | HIV-MDR-TB | HIV-XDR-TB | HIV-XDR-TB |
| --- | --- | --- | --- | --- | --- | --- | --- | --- |
|  | Incidence number  (95% UI)  2021 | Percentage change of incidence number (95% UI)  1990–2021 | Incidence number  (95% UI)  2021 | Percentage change of incidence number (95% UI)  1990–2021 | Incidence number  (95% UI)  2021 | Percentage change of incidence number  (95% UI)  1990–2021 | Incidence number  (95% UI)  2021 | Percentage change of incidence number (95% UI)  2010–2021 |
| Global | 1,645,333(1,484,721–1,822,432) | -0.18(-0.27, -0.07) | 955,221(854,661–107,5240) | 0.82(0.72–0.93) | 45,589(31,326–66,723) | 23.36(12.5–41.01) | 1606(1164–2183) | -0.03(-0.27, 0.27) |
| Male | 851,686(766,117–944,187) | -0.07(-0.18, 0.06) | 416,660(373,491–468,395) | 0.84(0.75–0.94) | 21,653(14,972–31,626) | 20.79(11.36–36.02) | 986(696–1369) | -0.04(-0.28, 0.27) |
| Female | 793,647(703,406–901,252) | -0.27(-0.36, -0.17) | 538,561(481,363–606,506) | 0.81(0.69–0.94) | 23,936(16,491–35,421) | 26.27(13.26–48.54) | 620(456–825) | -0.01(-0.26, 0.29) |
| East Asia | 34,453(17,655–52,755) | 3.2(0.63–5.63) | 21,606(18,376–24,540) | 1.98(1.47–3.08) | 1141(229–3390) | 2.69(-0.43, 18.41) | 104(21–312) | 0.23(-0.68, 2.3) |
| Southeast Asia | 102,476(78,577–14,1348) | 0.37(0.12–0.7) | 50,309(45,213–55,425) | 3.72(3.46–3.98) | 1723(880–3019) | 30.43(10.08–96.49) | 173(86–305) | -0.3(-0.66, 0.27) |
| Oceania | 4113(1898–7779) | 57.25(21.2–126.3) | 1196(1062–1336) | 32.4(27.29–47.65) | 54(16–132) | 2082.31(433.52–7365.13) | 7(2–18) | 3.04(-0.14, 12.85) |
| Central Asia | 21,600(14018–30,450) | 6.47(3.15–10.9) | 634(497–775) | 0.03(-0.18, 0.22) | 243(151–370) | 196.59(62.77–565.7) | 54(33–81) | -0.06(-0.39, 0.37) |
| Central Europe | 2845(2037–3900) | 2.15(1.46–3.05) | 382(328–438) | -0.4(-0.49, -0.22) | 9(4–18) | 0.62(-0.48, 3.95) | 2(1–5) | -0.24(-0.75, 1.16) |
| Eastern Europe | 145,560(116,674–182,918) | 11.66(8.43–16.71) | 5743(4122–7695) | -0.06(-0.3, 0.16) | 3435(2084–5233) | 36.75(13.6–91.54) | 724(440–1103) | 0.07(-0.36, 0.73) |
| High-income Asia Pacific | 4011(2331–5906) | 1.39(0.42–2.25) | 683(587–785) | -0.05(-0.26, 2.64) | 10(3–31) | 0.8(-0.68, 7.18) | 1(0–4) | 0.03(-0.67–2.05) |
| Australasia | 1767(1048–2575) | 0.41(-0.2, 1.14) | 43(37–50) | -0.72(-0.75, -0.7) | 2(1–4) | 0.72(-0.58, 7.2) | 0(0–0) | 1.38(-0.34, 7.32) |
| Western Europe | 29,244(24,363–34,334) | -0.43(-0.53, -0.32) | 1428(1241–1643) | -0.8(-0.8, -0.79) | 39(24–63) | -0.48(-0.75, 0.06) | 8(4–12) | -0.05(-0.42, 0.58) |
| Southern Latin America | 12,872(10,930–15,468) | 0.74(0.43–1.13) | 2838(2455–3312) | 0.16(0.06–0.26) | 46(12–142) | 3.78(-0.17, 25.86) | 9(2–26) | 0.2(-0.66, 2.11) |
| High-income North America | 62,127(25,619–97,752) | -0.03(-0.58, 0.79) | 758(651–882) | -0.73(-0.75, -0.71) | 14(5–33) | -0.89(-0.96, -0.66) | 2(1–6) | 0.45(-0.41, 2.64) |
| Caribbean | 20,181(12,252–31,456) | -0.52(-0.7, -0.3) | 2831(2495–3193) | -0.26(-0.33, -0.19) | 16(6–40) | -0.13(-0.79, 2.58) | 2(1–5) | 0.24(-0.6, 2.56) |
| Andean Latin America | 9607(7424–12,659) | 3.06(0.62–5.86) | 2768(2346–3224) | 0.37(0.18–0.61) | 240(108–493) | 12.03(2.9–49.11) | 21(10–44) | 0.12(-0.44, 1.21) |
| Central Latin America | 38,141(29,554–47,931) | 0.71(0.26–1.13) | 5919(5207–6769) | 0.18(0.08–0.29) | 224(96–453) | 25.09(7.76–74.47) | 25(11–49) | 0.45(-0.31, 1.84) |
| Tropical Latin America | 53,406(34,739–76,186) | 1.21(0.68–1.8) | 13,834(11,684–16,225) | 0.1(-0.02, 0.20) | 549(118–1539) | 76.91(8.75–729.3) | 49(11–135) | 0.86(-0.53, 4.27) |
| North Africa and Middle East | 26,786(11,435–70,371) | 6.66(0.72–33.79) | 5057(4445–5787) | 2(1.63–2.56) | 137(74–259) | 28.54(10.09–79.15) | 6(3–10) | 0.49(-0.28, 1.68) |
| South Asia | 98,389(59,786–171,965) | 3.9(1.53–9.15) | 68,347(56,745–80,154) | 10.43(8.74–12.27) | 6325(1714–14,984) | 940.6(107.2–9844.36) | 162(45–383) | -0.27(-0.78, 0.87) |
| Central Sub-Saharan Africa | 88,441(54,104–140,657) | -0.32(-0.6, 0.12) | 44,102(39,223–49,387) | 0.23(0.15–0.31) | 1169(434–2581) | 8.67(1.32–47.84) | 10(4–22) | -0.25(-0.74, 1.21) |
| Eastern Sub-Saharan Africa | 354,894(261,404–491,407) | -0.62(-0.72, -0.47) | 278,560(244,160–318,445) | 0.13(0.05–0.24) | 12,362(6912–20,664) | 56.38(20.59–149.74) | 107(60–180) | 0.14(-0.36, 1.01) |
| Southern Sub-Saharan Africa | 284,502(221,171–360,122) | -0.07(-0.3, 0.19) | 331,855(294,313–373,263) | 2.17(1.86–2.51) | 13,123(6235–28,58) | 30.25(7.74–131.14) | 98(48–206) | -0.21(-0.71, 0.95) |
| Western Sub-Saharan Africa | 249,917(207,057–297,319) | -0.16(-0.33 0.02) | 116,327(101,778–131,447) | 0.69(0.53–0.89) | 4728(2161–9842) | 11.73(3.37–37.32) | 42(20–84) | -0.18(-0.58, 0.59) |
| High-middle SDI | 194,553(161,398–236,339) | 2.51(1.89–3.3) | 17,695(15,021–20,857) | 0.23(0.11–0.36) | 3901(2406–5908) | 20.15(9.98–43.93) | 772(477–1161) | 0.07(-0.34, 0.65) |
| High SDI | 94,568(52,818–133,660) | -0.02(-0.4, 0.42) | 3386(2956–3884) | -0.62(-0.64–-0.59) | 102(65–167) | -0.5(-0.72, -0.1) | 16(11–23) | -0.02(-0.29, 0.4) |
| Low-middle SDI | 424,832(360,099–512,001) | -0.41(-0.5, -0.28) | 256,258(225,676–294,179) | 0.62(0.5–0.75) | 13194(7640–20,424) | 34.72(9.73–116.13) | 273(167–437) | -0.25(-0.53, 0.18) |
| Low SDI | 408,301(312,595–536,835) | -0.56(-0.66, -0.41) | 303,826(268,085–342,865) | 0.1(0.03–0.2) | 134,73(7954–21,579) | 22.06(8.51–50.98) | 144(89–225) | 0.07(-0.34, 0.69) |
| Middle SDI | 521,662(462,244–593,918) | 1.38(1.02–1.86) | 373,580(335,384–419,400) | 4.55(4.09–5.05) | 149,03(7505–29,959) | 27.28(8.7–83.17) | 401(239–612) | -0.04(-0.43, 0.59) |

Notes: Globally, the World Health Organization began to recommend the XDR-TB surveillance in 1991. Consequently, the number of incidence cases of HIV-XDR-TB has been tracked and reported since 1991. However, the GBD 2021 database provides total percentage change data for the periods 1990–2000, 2000–2021, 1990–2021, 2010–2021, and 2019–2021. Therefore, percentage change of the number of incidence cases for HIV-XDR-TB of 2010–2021 were used in the study. GBD: Global Burden of Disease. HIV-DS-TB: HIV-infected drug-susceptible tuberculosis. HIV-MDR-TB: HIV-infected multidrug-resistant tuberculosis without extensive drug resistance. HIV-XDR-TB: HIV-infected extensively drug-resistant tuberculosis).

Table S2 Age-standardized rates of HIV-DS-TB, HIV-MDR-TB, and HIV-XDR-TB in 2021 year, and percentage change of age-standardized rates in 204 countries and territories (Top five).

| ID | Sequence | Feature | Year | Index | Diseases | Nations | Value (percentage) (95%: UI ) |
| --- | --- | --- | --- | --- | --- | --- | --- |
| 1 | 1 | high | 2021 | incidence rate | HIV-DS-TB | Lesotho | 632.25(533.01–748.33) |
| 2 | 2 | high | 2021 | incidence rate | HIV-DS-TB | South Africa | 439.35(388.67–492.81) |
| 3 | 3 | high | 2021 | incidence rate | HIV-DS-TB | Eswatini | 433.63(338.32–527.63) |
| 4 | 4 | high | 2021 | incidence rate | HIV-DS-TB | Zimbabwe | 331.28(273.8–392.99) |
| 5 | 5 | high | 2021 | incidence rate | HIV-DS-TB | Mozambique | 310.6(259.27–362.4) |
| 6 | 1 | high | 2021 | incidence rate | HIV-MDR-TB | Eswatini | 57.01(13.63–148.73) |
| 7 | 2 | high | 2021 | incidence rate | HIV-MDR-TB | Lesotho | 37.76(10.63–96.13) |
| 8 | 3 | high | 2021 | incidence rate | HIV-MDR-TB | Mozambique | 20.11(5.44–48.91) |
| 9 | 4 | high | 2021 | incidence rate | HIV-MDR-TB | Zimbabwe | 17.94(2.9–57.12) |
| 10 | 5 | high | 2021 | incidence rate | HIV-MDR-TB | Namibia | 17.77(6.14–40.66) |
| 11 | 1 | high | 2021 | incidence rate | HIV-XDR-TB | Eswatini | 0.49(0.12–1.28) |
| 12 | 2 | high | 2021 | incidence rate | HIV-XDR-TB | Ukraine | 0.46(0.21–0.81) |
| 13 | 3 | high | 2021 | incidence rate | HIV-XDR-TB | Lesotho | 0.38(0.11–0.98) |
| 14 | 4 | high | 2021 | incidence rate | HIV-XDR-TB | Russian Federation | 0.29(0.15–0.47) |
| 15 | 5 | high | 2021 | incidence rate | HIV-XDR-TB | Republic of Moldova | 0.19(0.11–0.29) |
| 16 | 1 | high | 2021 | Prevalence rate | HIV-DS-TB | Lesotho | 1069.94(883.58–1273.29) |
| 17 | 2 | high | 2021 | Prevalence rate | HIV-DS-TB | South Africa | 704.5(618.79–792.43) |
| 18 | 3 | high | 2021 | Prevalence rate | HIV-DS-TB | Eswatini | 689(527.66–841.08) |
| 19 | 4 | high | 2021 | Prevalence rate | HIV-DS-TB | Mozambique | 577.81(479.93–677.78) |
| 20 | 5 | high | 2021 | Prevalence rate | HIV-DS-TB | Zimbabwe | 515.99(422.93–612.33) |
| 21 | 1 | high | 2021 | Prevalence rate | HIV-MDR-TB | Eswatini | 84.76(20.53–216.72) |
| 22 | 2 | high | 2021 | Prevalence rate | HIV-MDR-TB | Lesotho | 58.49(16.4–149.29) |
| 23 | 3 | high | 2021 | Prevalence rate | HIV-MDR-TB | Mozambique | 35.72(9.9–85.41) |
| 24 | 4 | high | 2021 | Prevalence rate | HIV-MDR-TB | Namibia | 29.53(10.39–66.16) |
| 25 | 5 | high | 2021 | Prevalence rate | HIV-MDR-TB | Zimbabwe | 26.65(4.34–84.55) |
| 26 | 1 | high | 2021 | Prevalence rate | HIV-XDR-TB | Eswatini | 0.53(0.13–1.35) |
| 27 | 2 | high | 2021 | Prevalence rate | HIV-XDR-TB | Ukraine | 0.38(0.17–0.66) |
| 28 | 3 | high | 2021 | Prevalence rate | HIV-XDR-TB | Lesotho | 0.36(0.1–0.93) |
| 29 | 4 | high | 2021 | Prevalence rate | HIV-XDR-TB | Mozambique | 0.22(0.06–0.53) |
| 30 | 5 | high | 2021 | Prevalence rate | HIV-XDR-TB | Myanmar | 0.22(0.07–0.52) |
| 31 | 1 | high | 2021 | Death rate | HIV-DS-TB | Lesotho | 168.02(124.82–209.72) |
| 32 | 2 | high | 2021 | Death rate | HIV-DS-TB | Eswatini | 100.31(59.52–136.53) |
| 33 | 3 | high | 2021 | Death rate | HIV-DS-TB | Botswana | 73.61(42.6–102.12) |
| 34 | 4 | high | 2021 | Death rate | HIV-DS-TB | Zimbabwe | 69.41(50.48–89.24) |
| 35 | 5 | high | 2021 | Death rate | HIV-DS-TB | Mozambique | 64.72(48.33–79.34) |
| 36 | 1 | high | 2021 | Death rate | HIV-MDR-TB | Eswatini | 168.02(124.82–209.72) |
| 37 | 2 | high | 2021 | Death rate | HIV-MDR-TB | Lesotho | 100.31(59.52–136.53) |
| 38 | 3 | high | 2021 | Death rate | HIV-MDR-TB | Mozambique | 73.61(42.6–102.12) |
| 39 | 4 | high | 2021 | Death rate | HIV-MDR-TB | Botswana | 69.41(50.48–89.24) |
| 40 | 5 | high | 2021 | Death rate | HIV-MDR-TB | Zimbabwe | 64.72(48.33–79.34) |
| 41 | 1 | high | 2021 | Death rate | HIV-XDR-TB | Eswatini | 0.39(0.08–1.01) |
| 42 | 2 | high | 2021 | Death rate | HIV-XDR-TB | Lesotho | 0.31(0.07–0.85) |
| 43 | 3 | high | 2021 | Death rate | HIV-XDR-TB | Ukraine | 0.21(0.1–0.38) |
| 44 | 4 | high | 2021 | Death rate | HIV-XDR-TB | Mozambique | 0.13(0.03–0.34) |
| 45 | 5 | high | 2021 | Death rate | HIV-XDR-TB | Botswana | 0.13(0.02–0.42) |
| 46 | 1 | high | 2021 | DALY rate | HIV-DS-TB | Lesotho | 8437.49(6417.95–10181.81) |
| 47 | 2 | high | 2021 | DALY rate | HIV-DS-TB | Eswatini | 5107.97(3123.54–6573.07) |
| 48 | 3 | high | 2021 | DALY rate | HIV-DS-TB | Mozambique | 3602.19(2723.92–4373.13) |
| 49 | 4 | high | 2021 | DALY rate | HIV-DS-TB | Botswana | 3366.64(2002–4528.86) |
| 50 | 5 | high | 2021 | DALY rate | HIV-DS-TB | South Africa | 3306.93(2732.02–3664.86) |
| 51 | 1 | high | 2021 | DALY rate | HIV-MDR-TB | Eswatini | 1400.14(333.13–3392.58) |
| 52 | 2 | high | 2021 | DALY rate | HIV-MDR-TB | Lesotho | 1086.24(267.83–2875.82) |
| 53 | 3 | high | 2021 | DALY rate | HIV-MDR-TB | Mozambique | 513.02(121.14–1308.49) |
| 54 | 4 | high | 2021 | DALY rate | HIV-MDR-TB | Botswana | 412.89(74.53–1257.72) |
| 55 | 5 | high | 2021 | DALY rate | HIV-MDR-TB | Zimbabwe | 374.96(57.7–1173.28) |
| 56 | 1 | high | 2021 | DALY rate | HIV-XDR-TB | Eswatini | 19.21(4.02–51.09) |
| 57 | 2 | high | 2021 | DALY rate | HIV-XDR-TB | Lesotho | 15(3.41–40.65) |
| 58 | 3 | high | 2021 | DALY rate | HIV-XDR-TB | Ukraine | 11.06(4.97–19.58) |
| 59 | 4 | high | 2021 | DALY rate | HIV-XDR-TB | Mozambique | 7.01(1.63–18) |
| 60 | 5 | high | 2021 | DALY rate | HIV-XDR-TB | Botswana | 5.72(0.93–18.55) |
| 61 | 1 | high | 1990-2021 | incidence rate | HIV-DS-TB | Pakistan | 259.06(68.25–5353.27) |
| 62 | 2 | high | 1990-2021 | incidence rate | HIV-DS-TB | Bangladesh | 117.35(97.14–145.98) |
| 63 | 3 | high | 1990-2021 | incidence rate | HIV-DS-TB | Philippines | 106.43(99.77–112.39) |
| 64 | 4 | high | 1990-2021 | incidence rate | HIV-DS-TB | Cambodia | 65.66(54.95–77.97) |
| 65 | 5 | high | 1990-2021 | incidence rate | HIV-DS-TB | Lao People's Democratic Republic | 40(33.42–48.61) |
| 66 | 1 | high | 1990-2021 | incidence rate | HIV-MDR-TB | Pakistan | 8644.21(1059.97–107,650.48) |
| 67 | 2 | high | 1990-2021 | incidence rate | HIV-MDR-TB | Philippines | 4770.4(663.97–64,768.96) |
| 68 | 3 | high | 1990-2021 | incidence rate | HIV-MDR-TB | Djibouti | 2530.53(414.13–28,750.82) |
| 69 | 4 | high | 1990-2021 | incidence rate | HIV-MDR-TB | Papua New Guinea | 1939.9(256.19–24,720.93) |
| 70 | 5 | high | 1990-2021 | incidence rate | HIV-MDR-TB | Madagascar | 1668.02(199.3–16598.14) |
| 71 | 1 | high | 2010-2021 | incidence rate | HIV-XDR-TB | Vanuatu | 3.9(-0.3, 34.36) |
| 72 | 2 | high | 2010-2021 | incidence rate | HIV-XDR-TB | Micronesia (Federated States of) | 2.64(-0.5, 22.3) |
| 73 | 3 | high | 2010-2021 | incidence rate | HIV-XDR-TB | Pakistan | 2.59(-0.2, 12.47) |
| 74 | 4 | high | 2010-2021 | incidence rate | HIV-XDR-TB | Solomon Islands | 2.3(-0.53, 21.52) |
| 75 | 5 | high | 2010-2021 | incidence rate | HIV-XDR-TB | Papua New Guinea | 2.13(-0.34, 9.79) |
| 76 | 1 | high | 1990-2021 | Prevalence rate | HIV-DS-TB | Pakistan | 415.06(98.5–9041.3) |
| 77 | 2 | high | 1990-2021 | Prevalence rate | HIV-DS-TB | Bangladesh | 179.77(148.45–220.17) |
| 78 | 3 | high | 1990-2021 | Prevalence rate | HIV-DS-TB | Cambodia | 168.43(141.41–201.78) |
| 79 | 4 | high | 1990-2021 | Prevalence rate | HIV-DS-TB | Lao People's Democratic Republic | 101.91(84.29–122.07) |
| 80 | 5 | high | 1990-2021 | Prevalence rate | HIV-DS-TB | Mongolia | 101.01(0–0) |
| 81 | 1 | high | 1990-2021 | Prevalence rate | HIV-MDR-TB | Pakistan | 51,846.63(5576.97–3,386,620.48) |
| 82 | 2 | high | 1990-2021 | Prevalence rate | HIV-MDR-TB | Bangladesh | 14805.45(2339.48–179,404.73) |
| 83 | 3 | high | 1990-2021 | Prevalence rate | HIV-MDR-TB | Mongolia | 9468.29(0–0) |
| 84 | 4 | high | 1990-2021 | Prevalence rate | HIV-MDR-TB | Papua New Guinea | 6454.69(833.16–107,668.17) |
| 85 | 5 | high | 1990-2021 | Prevalence rate | HIV-MDR-TB | Philippines | 3816.45(533.29–51,503.14) |
| 86 | 1 | high | 2010-2021 | Prevalence rate | HIV-XDR-TB | Vanuatu | 3.12(-0.43, 28.52) |
| 87 | 2 | high | 2010-2021 | Prevalence rate | HIV-XDR-TB | Comoros | 2.99(-0.39 , 18.7) |
| 88 | 3 | high | 2010-2021 | Prevalence rate | HIV-XDR-TB | Papua New Guinea | 2.87(-0.19, 13.18) |
| 89 | 4 | high | 2010-2021 | Prevalence rate | HIV-XDR-TB | Pakistan | 2.64(-0.21, 13.19) |
| 90 | 5 | high | 2010-2021 | Prevalence rate | HIV-XDR-TB | Micronesia (Federated States of) | 2.25(-0.55, 19.72) |
| 91 | 1 | high | 1990-2021 | Death rate | HIV-DS-TB | Pakistan | 348738(32,338.87–30,390,154.05) |
| 92 | 2 | high | 1990-2021 | Death rate | HIV-DS-TB | Cambodia | 28837.11(2200.18–403,870.07) |
| 93 | 3 | high | 1990-2021 | Death rate | HIV-DS-TB | Papua New Guinea | 27859.33(4391.97–429,677.98) |
| 94 | 4 | high | 1990-2021 | Death rate | HIV-DS-TB | Djibouti | 21848.49(2983.26–705,878.24) |
| 95 | 5 | high | 1990-2021 | Death rate | HIV-DS-TB | Bangladesh | 13956.18(2441.96–158,384.28) |
| 96 | 1 | high | 1990-2021 | Death rate | HIV-MDR-TB | Cambodia | 3119.84(1346.41–8246.78) |
| 97 | 2 | high | 1990-2021 | Death rate | HIV-MDR-TB | Pakistan | 3101.39(469.84–86,773.16) |
| 98 | 3 | high | 1990-2021 | Death rate | HIV-MDR-TB | Lao People's Democratic Republic | 390.31(218.59–956.01) |
| 99 | 4 | high | 1990-2021 | Death rate | HIV-MDR-TB | Papua New Guinea | 310.29(123.74–1248.63) |
| 100 | 5 | high | 1990-2021 | Death rate | HIV-MDR-TB | Bangladesh | 184.25(125.86–331.07) |
| 101 | 1 | high | 2010-2021 | Death rate | HIV-XDR-TB | Vanuatu | 3.91(-0.3, 34.96) |
| 102 | 2 | high | 2010-2021 | Death rate | HIV-XDR-TB | Micronesia (Federated States of) | 2.63(-0.51, 25.62) |
| 103 | 3 | high | 2010-2021 | Death rate | HIV-XDR-TB | Pakistan | 2.59(-0.27, 17.25) |
| 104 | 4 | high | 2010-2021 | Death rate | HIV-XDR-TB | Solomon Islands | 2.27(-0.59, 24.38) |
| 105 | 5 | high | 2010-2021 | Death rate | HIV-XDR-TB | Nauru | 2.21(-0.59, 19.41) |
| 106 | 1 | high | 1990-2021 | DALY rate | HIV-DS-TB | Pakistan | 2180.61(447.27–47,103.01) |
| 107 | 2 | high | 1990-2021 | DALY rate | HIV-DS-TB | Cambodia | 962.58(663.82–1417.72) |
| 108 | 3 | high | 1990-2021 | DALY rate | HIV-DS-TB | Lao People's Democratic Republic | 320.93(206.42–569.77) |
| 109 | 4 | high | 1990-2021 | DALY rate | HIV-DS-TB | Papua New Guinea | 194.76(90.53–614.6) |
| 110 | 5 | high | 1990-2021 | DALY rate | HIV-DS-TB | Bangladesh | 187.5(139.79–268.18) |
| 111 | 1 | high | 1990-2021 | DALY rate | HIV-MDR-TB | Pakistan | 289209.18(28790.75–19,555,308.01) |
| 112 | 2 | high | 1990-2021 | DALY rate | HIV-MDR-TB | Papua New Guinea | 20470.46(3126.24–289,384.58) |
| 113 | 3 | high | 1990-2021 | DALY rate | HIV-MDR-TB | Djibouti | 16957.89(2429.5–468,060.97) |
| 114 | 4 | high | 1990-2021 | DALY rate | HIV-MDR-TB | Bangladesh | 14372.77(2509.96–164,504.8) |
| 115 | 5 | high | 1990-2021 | DALY rate | HIV-MDR-TB | Cambodia | 14048.15(1118.06–169,029.49) |
| 116 | 1 | high | 2010-2021 | DALY rate | HIV-XDR-TB | Vanuatu | 3.82(-0.31, 34.38) |
| 117 | 2 | high | 2010-2021 | DALY rate | HIV-XDR-TB | Micronesia (Federated States of) | 2.57(-0.52, 24.73) |
| 118 | 3 | high | 2010-2021 | DALY rate | HIV-XDR-TB | Pakistan | 2.46(-0.28, 15.06) |
| 119 | 4 | high | 2010-2021 | DALY rate | HIV-XDR-TB | Tuvalu | 2.23(-0.55, 25.23) |
| 120 | 5 | high | 2010-2021 | DALY rate | HIV-XDR-TB | Cook Islands | 2.22(-0.50, 22.33) |

Notes: Globally, the World Health Organization began to recommend the XDR-TB surveillance in 1991. Consequently, the age-standardized incidence rate of HIV-XDR-TB has been tracked and reported since 1993, age-standardized prevalence rate, age-standardized mortality rate and age-standardized DALY rate has been tracked and reported since 1991. However, the GBD 2021 database provides total percentage change data for the periods 1990–2000, 2000–2021, 1990–2021, 2010–2021, and 2019–2021. Therefore, percentage change of ASRs for XDR-TB of 2010–2021 were used in the study. ASR: age-standardized rate. GBD: Global Burden of Disease. HIV-DS-TB: HIV-infected drug-susceptible tuberculosis. HIV-MDR-TB: HIV-infected multidrug-resistant tuberculosis without extensive drug resistance. HIV-XDR-TB: HIV-infected extensively drug-resistant tuberculosis.

Table S3 The number of prevalence cases of HIV, HIV-DS-TB, HIV-MDR-TB, and HIV-XDR-TB individuals in 2021, and percentage change of number of prevalence cases were analyzed across GBD regions.

| Region | HIV/AIDS | HIV/AIDS | HIV-DS-TB | HIV-DS-TB | HIV-MDR-TB | HIV-MDR-TB | HIV-XDR-TB | HIV-XDR-TB |
| --- | --- | --- | --- | --- | --- | --- | --- | --- |
|  | Prevalence cases  (95% UI)  2021 | Percentage change of prevalence cases  (95% U)  1990–2021 | Prevalence cases  (95% UI)  2021 | Percentage change of prevalence cases (95% U)  1990–2021 | Prevalence cases  (95% UI)  2021 | Percentage change of prevalence case  (95% U)  1990–2021 | Prevalence cases (95% UI)  2021 | Percentage change of prevalence cases (95% U)  2010–2021 |
| Global | 40,036,936(38,036,249–42,372,669) | 4.05(3.68–4.47) | 1,682,115(1,494,990–1,881.082) | 0.68(0.58–0.8) | 71,455(48,999–106,009) | 13.7(25.57–0.8) | 1727(1241–2427) | 0.06(-0.2, 25.57) |
| Global male | 17,952,494(16,582,924–19,402,060) | 3.59(3.2–4.05) | 719,793(642,869–799,879) | 0.75(0.65–0.86) | 32,592(22,182–47,827) | 23.98(12.93–40.91) | 1027(727–1476) | 0.04(-0.24, 0.47) |
| Global female | 22,084,442(21,164,831–23,205,503) | 4.48(4.05–4.99) | 962,322(851,723–1,078,035) | 0.64(0.52–0.77) | 38,863(26,508–56,788) | 27.06(13.81–49.64) | 700(518–972) | 0.1(-0.17, 0.47) |
| East Asia | 575,501(309,988–1,027,198) | 11.52(7.19–19.54) | 43,218(35,661–50,080) | 2.99(2.17–4.42) | 2237(381–6907) | -0.18(5.29–4.42) | 196(33–605) | 0.35(-0.69, 5.29) |
| Southeast Asia | 1,549,666(1,209,139–2,060,608) | 15.49(13.19–17.71) | 93,848(83,549–104,118) | 4.49(4.05–5.09) | 3166(1615–5526) | 12.74(40.06–5.09) | 277(142–484) | -0.14(-0.58, 40.06) |
| Oceania | 6,0649(43,722–84,376) | 269.33(183.68–389.67) | 4095(3623–4566) | 64.04(45.64–87.51) | 172(50–408) | 1062.05(4718.31–87.51) | 15(4–36) | 4.19(0.09–4718.31) |
| Central Asia | 71,184(56,829–94,354) | 13.9(9.74–18.87) | 890(694–1099) | -0.1(-0.29, 0.12) | 291(182–445) | 59.64(191.96–0.12) | 61(38–94) | -0.03(-0.38, 191.96) |
| Central Europe | 38,201(25,909–52,401) | 5.94(4.42–8.37) | 476(412–551) | -0.42(-0.53, -0.15) | 10(5–20) | -0.29(1.08, -0.15) | 2(1–4) | -0.11(-0.69, 1.08) |
| Eastern Europe | 1,459,394(1,141,975–1,807,561) | 18.63(13.27–23.66) | 5267(3764–6959) | -0.1(-0.33, 0.12) | 2507(1577–3672) | 10.47(27.18–0.12) | 527(332–772) | -0.03(-0.41, 27.18) |
| High-income Asia Pacific | 6,2531(36,868–91,634) | 8.58(5.84–14.26) | 495(413–591) | -0.28(-0.44, 1.27) | 6(2–19) | -0.77(0.41–1.27) | 1(0–2) | -0.02(-0.69, 0.41) |
| Australasia | 22,030(14,034–30,215) | 1.14(0.84–1.42) | 34(29–40) | -0.76(-0.78, -0.74) | 1(0–3) | -0.64(0.45–-0.74) | 0(0–0) | 1.13(-0.38, 0.45) |
| Western Europe | 997,042(798,249–1,166,763) | 1.52(1.35–1.68) | 1590(1363–1848) | -0.83(-0.84, -0.82) | 40(25–63) | -0.71(-0.42, -0.82) | 5(3–8) | 0.04(-0.34, -0.42) |
| Southern Latin America | 229,508(195,308–270,809) | 3.29(2.83–3.82) | 3695(3189–4243) | 0.04(-0.04, 0.12) | 54(13–176) | -0.21(3.31–0.12) | 6(2–21) | 0.18(-0.66, 3.31) |
| High-income North America | 1,823,069(1,009,261–2,663,601) | 0.99(0.54–1.41) | 950(802–1124) | -0.77(-0.79, -0.76) | 16(6–42) | -0.96(-0.88, -0.76) | 2(1–5) | 0.46(-0.43, -0.88) |
| Caribbean | 358,394(310,645–406,615) | 1.26(0.69–2.18) | 3962(3386–4598) | -0.28(-0.34, -0.22) | 22(9–51) | -0.68(0.16–-0.22) | 2(1–4) | 0.45(-0.48, 0.16) |
| Andean Latin America | 147,211(115,558–183,852) | 11.38(8.51–15.22) | 3488(2938–4101) | 0.29(0.07–0.52) | 283(125–581) | 2.75(11.49–0.52) | 22(10–44) | 0.25(-0.37, 11.49) |
| Central Latin America | 529,405(398,838–684,145) | 7.4(6.05–8.9) | 9454(8190–10,881) | 0.1(0–0.21) | 334(136–693) | 8.39(28.86–0.21) | 25(10–53) | 0.72(-0.21, 28.86) |
| Tropical Latin America | 705,505(451,488–992,673) | 3.36(2.72–4.02) | 15,172(12,930–17,615) | -0.15(-0.24, -0.07) | 545(120–1563) | 7.63(64.45–-0.07) | 41(9–119) | 0.97(-0.5, 64.45) |
| North Africa and Middle East | 229,286(142,519–406,822) | 17.24(4.95–43.44) | 5916(5149–6779) | 2.19(1.76–2.76) | 158(88–278) | 17.47(44.55–2.76) | 5(3–10) | 0.71(-0.15, 44.55) |
| South Asia | 2,110,424(1,770,014–2,573,655) | 60.54(41.82–90.3) | 105,791(88,304–123,954) | 11.47(9.33–14.2) | 8826(2377–21121) | 120.43(1111.12–14.2) | 209(56–500) | -0.01(-0.7, 1111.12) |
| Central Sub-Saharan Africa | 1,237,893(1,047,503–1,434,515) | 1.54(1.02–2.28) | 97431(86,636–108,994) | 0.16(0.08–0.24) | 2489(926–5600) | 1.26(8.82–0.24) | 16(6–35) | 0.09(-0.61, 8.82) |
| Eastern Sub-Saharan Africa | 11,480,329(10,637,773–1,2631,901) | 2.22(1.89–2.73) | 522,225(453,808–590,926) | 0.01(-0.07, 0.11) | 21,595(12,291–36,375) | 21.2(55.56–0.11) | 135(77–227) | 0.7(-0.07, 55.56) |
| Southern Sub-Saharan Africa | 11,549,270(11,064,897–12,052,321) | 12.82(11.14–14.59) | 530,816(469,127–602,263) | 2.54(2.15–3.01) | 20,033(9192–42,753) | 9.27(39.74–3.01) | 125(57–267) | 0.09(-0.62, 39.74) |
| Western Sub-Saharan Africa | 4,800,443(4,517,421–5,079,614) | 3.8(2.97–4.74) | 233,301(203,029–263,919) | 0.55(0.41–0.76) | 8671(4121–17,157) | 3.22(10.68–0.76) | 54(26–107) | -0.02(-0.5, 10.68) |
| High-middle SDI | 2,410,263(1,982,432–2,874,836) | 5.65(4.87–6.57) | 25,894(22,233–29,755) | 0.51(0.37–0.67) | 3261(2103–5154) | 7.86(17.42–0.67) | 591(381–872) | -0.01(-0.39, 17.42) |
| High SDI | 2,538,148(1,581,354–3,510,854) | 1.24(0.88–1.59) | 4041(3487–4669) | -0.68(-0.7, -0.67) | 129(83–213) | -0.67(-0.39, -0.67) | 16(11–25) | 0.07(-0.24, -0.39) |
| Low-middle SDI | 9,876,631(9,372,625–10,386,889) | 3.9(3.39–4.49) | 453,520(399,294–518,011) | 0.57(0.46–0.72) | 20,919(12,369–32,076) | 10.18(34.94–0.72) | 377(217–622) | -0.06(-0.44, 34.94) |
| Low SDI | 1,1499,846(10,632,720–12,671,653) | 1.97(1.64–2.39) | 596,879(523,034–669,614) | 0.03(-0.05, 0.12) | 24,483(14,805–38,104) | 8.12(21.27–0.12) | 192(117–296) | 0.5(-0.06, 21.27) |
| Middle SDI | 1,3683,296(12,746,460–14,849,390) | 24.09(21.64–27.5) | 600,861(536,310–673,728) | 5.03(4.38–5.76) | 22,632(11,424–45,654) | 11.77(35.69–5.76) | 550(331–914) | 0.14(-0.34, 35.69) |

Notes: Globally, the World Health Organization began to recommend the XDR-TB surveillance in 1991. Consequently, the number of prevalence cases of HIV-XDR-TB has been tracked and reported since 1991. However, the GBD 2021 database provides total percentage change data for the periods 1990–2000, 2000–2021, 1990–2021, 2010–2021, and 2019–2021. Therefore, percentage change of the number of prevalence cases for HIV-XDR-TB spanning 2010–2021 were used in the study. GBD: Global Burden of Disease. HIV-DS-TB: HIV-infected drug-susceptible tuberculosis. HIV-MDR-TB: HIV-infected multidrug-resistant tuberculosis without extensive drug resistance. HIV-XDR-TB: HIV-infected extensively drug-resistant tuberculosis.

Table S4 The number of death cases of HIV, HIV-DS-TB, HIV-MDR-TB, and HIV-XDR-TB individuals in 2021, and percentage change of number of death cases were analyzed across GBD regions

| Region | HIV/AIDS | HIV/AIDS | HIV-DS-TB | HIV-DS-TB | HIV-MDR-TB | HIV-MDR-TB | HIV-XDR-TB | HIV-XDR-TB |
| --- | --- | --- | --- | --- | --- | --- | --- | --- |
|  | Death cases  (95% UI)  2021 | Percentage change of death cases  (95% UI)  1990–2021 | Death cases  (95% UI)  2021 | Percentage change of death cases  (95% UI)  1990–2021 | Death cases  (95% UI)  2021 | Percentage change of death cases  (95% UI)  1990–2021 | Death cases  (95% UI)  2021 | Percentage change of death cases  (95% UI)  2010–2021 |
| Global | 718,079(669,271–785,447) | 1.35(0.89–25.57) | 182,597(141,923–225,076) | 0.64(0.23–25.57) | 17,458(7574–32,229) | 23.96(13.75–25.57) | 840(385–1492) | -0.26(-0.43, 25.57) |
| Male | 354,674(330,935–389,943) | 1.25(0.85–1.82) | 86,958(68,033–107082) | 0.7(0.3–1.29) | 8313(3674–15,181) | 20.61(12.31–36.56) | 507(235–894) | -0.28(-0.44, -0.07) |
| Female | 363,405(331,298–406,712) | 1.44(0.87–2.33) | 95,639(73,892–117,048) | 0.59(0.15–1.33) | 9145(3964–17,117) | 28.06(14.63–56.67) | 333(151–581) | -0.22(-0.4, -0.01) |
| East Asia | 32,999(23,836–41,952) | 20.63(9.69–5.29) | 3092(1847–4602) | 3.9(1.3–5.29) | 311(51–942) | 5.68(-0.17, 5.29) | 61(9–204) | 0.12(-0.71, 5.29) |
| Southeast Asia | 33,435(31,188–37,365) | 2.05(1.84–40.06) | 9419(7338–11748) | 5.66(4.33–40.06) | 616(227–1255) | 33.45(9.2–40.06) | 120(41–278) | -0.34(-0.68, 40.06) |
| Oceania | 956(667–1631) | 42.96(29.69–4718.31) | 333(236–486) | 76.55(48.73–4718.31) | 30(7–76) | 3383.05(836.33–4718.31) | 6(1–15) | 3.04(-0.09, 4718.31) |
| Central Asia | 1467(1464–1469) | 2.66(2.65–191.96) | 96(51–165) | -0.33(-0.57, 191.96) | 55(25–90) | 107.05(34.11–191.96) | 26(12–45) | -0.28(-0.48, 191.96) |
| Central Europe | 454(453–455) | -0.04(-0.05, 1.08) | 71(48–104) | -0.56(-0.62, 1.08) | 3(1–8) | 0.24(-0.61, 1.08) | 2(0–4) | -0.26(-0.75, 1.08) |
| Eastern Europe | 26,411(26,338–26,478) | 4.25(4.23–27.18) | 675(321–1260) | -0.24(-0.59, 27.18) | 555(280–924) | 18.4(6.99–27.18) | 259(127–429) | -0.37(-0.56, 27.18) |
| High-income Asia Pacific | 329(329–329) | 2.28(2.28–0.41) | 114(92–130) | 1.59(1.11–0.41) | 3(1–9) | 3.24(-0.02, 0.41) | 1(0–2) | 0.12(-0.62, 0.41) |
| Australasia | 74(74–74) | -0.83(-0.83, 0.45) | 5(3–8) | -0.83(-0.86, 0.45) | 0(0–1) | -0.01(-0.75, 0.45) | 0(0–0) | 0.93(-0.47, 0.45) |
| Western Europe | 2915(2912–2918) | -0.71(-0.71, -0.42) | 413(267–625) | -0.85(-0.87, -0.42) | 21(7–47) | -0.57(-0.8, -0.42) | 6(2–12) | -0.06(-0.41, -0.42) |
| Southern Latin America | 2412(2405–2419) | 2.19(2.19–3.31) | 807(527–1023) | 1.4(0.59–3.31) | 25(5–83) | 7.66(0.57–3.31) | 7(1–23) | 0.19(-0.65, 3.31) |
| High-income North America | 6483(6481–6485) | -0.77(-0.77, -0.88) | 175(111–269) | -0.86(-0.87, -0.88) | 6(2–19) | -0.93(-0.98, -0.88) | 2(0–5) | 0.43(-0.41, -0.88) |
| Caribbean | 7701(6462–9292) | -0.03(-0.29, 0.16) | 864(467–2143) | -0.51(-0.63, 0.16) | 10(2–32) | -0.24(-0.83, 0.16) | 2(0–6) | 0.22(-0.63, 0.16) |
| Andean Latin America | 2976(2798–3228) | 6.58(5.91–11.49) | 494(297–795) | 1.85(0.74–11.49) | 89(28–196) | 23.82(5.96–11.49) | 15(4–36) | -0.01(-0.46, 11.49) |
| Central Latin America | 12,266(12,253–12,279) | 1.8(1.8–28.86) | 1550(990–2286) | -0.07(-0.28, 28.86) | 114(34–247) | 21.05(6.92–28.86) | 19(6–45) | 0.43(-0.26, 28.86) |
| Tropical Latin America | 15,244(15,221–15,273) | 0.99(0.98–64.45) | 2521(1547–3858) | -0.1(-0.33, 64.45) | 190(32–593) | 57.55(7.09–64.45) | 32(5–110) | 0.71(-0.53, 64.45) |
| North Africa and Middle East | 11,120(6,905–22,268) | 15.2(5.39–44.55) | 801(544–1116) | 2.46(1.22–44.55) | 48(16–100) | 38.36(13.98–44.55) | 4(1–8) | 0.36(-0.29, 44.55) |
| South Asia | 45,796(34,370–67,841) | 186.14(82.18–1111.12) | 9903(6578–13,499) | 89.15(39.38–1111.12) | 1691(398–3937) | 6706.61(977.22–1111.12) | 89(20–228) | -0.44(-0.82, 1111.12) |
| Central Sub-Saharan Africa | 35,873(28,747–46,983) | 0.89(0.29–8.82) | 9276(6842–12,280) | 0.1(-0.28, 8.82) | 560(167–1348) | 8.04(0.95–8.82) | 8(2–22) | -0.27(-0.72, 8.82) |
| Eastern Sub-Saharan Africa | 200,795(178,848–227,048) | 0.37(0.01–55.56) | 62,078(47,086–77,528) | -0.03(-0.34, 55.56) | 5926(2419–12,025) | 60.69(20.87–55.56) | 82(32–174) | 0.11(-0.35, 55.56) |
| Southern Sub-Saharan Africa | 133,814(127,327–141,095) | 4.68(2.97–39.74) | 52,778(44,114–58,185) | 4.03(2.47–39.74) | 4877(1804–10,740) | 60.67(13.59–39.74) | 68(23–164) | -0.29(-0.7, 39.74) |
| Western Sub-Saharan Africa | 144,560(120,982–17,6310) | 2.89(1.93–10.68) | 27,134(17,948–39,120) | 1(0.42–10.68) | 2328(715–5439) | 10.85(3.1–10.68) | 32(10–73) | -0.18(-0.57, 10.68) |
| High-middle SDI | 41,673(39,558–44,903) | 2.62(2.42–17.42) | 2923(1790–4322) | 0.2(-0.01, 17.42) | 682(343–1130) | 11.72(5.63–17.42) | 286(143–481) | -0.35(-0.54, 17.42) |
| High SDI | 10,346(10,260–10,424) | -0.71(-0.71, -0.39) | 710(469–1058) | -0.8(-0.82, -0.39) | 38(15–81) | -0.71(-0.84–-0.39) | 10(4–20) | -0.11(-0.36, -0.39) |
| Low-middle SDI | 227,955(200,105–266,287) | 2.52(1.65–34.94) | 54,499(40,048–71,754) | 1(0.37–34.94) | 5304(2160–10,459) | 41.82(13.23–34.94) | 172(68–320) | -0.36(-0.59, 34.94) |
| Low SDI | 221,819(197,858–252,794) | 0.34(0.02–21.27) | 66,685(51,532–81,944) | -0.05(-0.31, 21.27) | 6542(2634–12,882) | 20.43(8.14–21.27) | 109(42–219) | 0.04(-0.34, 21.27) |
| Middle SDI | 215,741(206,054–226,473) | 6.88(6.26–35.69) | 57,666(45,977–67,017) | 6.59(5.43, 35.69) | 4884(1663–10,665) | 56.11(20.16–35.69) | 263(92–538) | -0.14(-0.47, 35.69) |

Notes: Globally, the World Health Organization began to recommend the XDR-TB surveillance in 1991. Consequently, the number of death cases of HIV-XDR-TB has been tracked and reported since 1993. However, the GBB 2021 database provides total percentage change data for the periods 1990－2000, 2000－2021, 1990－2021, 2010－2021, and 2019－2021. Therefore, percentage change of number of death cases for HIV-XDR-TB spanning 2010–2021 were used in the study. GBD: Global Burden of Disease. HIV-DS-TB: HIV-infected drug-susceptible tuberculosis. HIV-MDR-TB: HIV-infected multidrug-resistant tuberculosis without extensive drug resistance. HIV-XDR-TB: HIV-infected extensively drug-resistant tuberculosis.

Table S5 The number of DALY cases of HIV, HIV-DS-TB, HIV-MDR-TB, and HIV-XDR-TB individuals in 2021, and percentage change of the number of DALY cases were analyzed across GBD regions

| Region | HIV/AIDS | HIV/AIDS | HIV-DS-TB | HIV-DS-TB | HIV-MDR-TB | HIV-MDR-TB | HIV-XDR-TB | HIV-XDR-TB |
| --- | --- | --- | --- | --- | --- | --- | --- | --- |
|  | DALY cases  (95% UI)  2021 | Percentage change of DALY cases  (95% UI)  1990–2021 | DALY cases  (95% UI)  2021 | Percentage change of DALY cases (95% UI)  1990–2021 | DALY cases  (95% UI)  2021 | Percentage change of DALY cases  (95% UI)  1990–2021 | DALY cases  (95% UI)  2021 | Percentage change of DALY cases (95% UI)  2010–2021 |
| Global | 40,266,792(37,13,092–44,796,387) | 1.16(0.78–25.57) | 9,910,866(7,825,966–1,211,0494) | 0.43(0.1–25.57) | 925,471(413,530–1,668,293) | 21.74(12.31–25.57) | 42,095(1,9698–74,093) | -0.3(-0.45, 25.57) |
| Male | 19,157,019(17,890,274–21,178,799) | 1.06(0.76–1.49) | 4,553,416(3,616,328–5,560,144) | 0.47(0.17–0.9) | 426,216(194,728–770,792) | 18.8(11.19–33.09) | 25,007(11,748–43,661) | -0.32(-0.47, -0.12) |
| Female | 21,109,773(19,118,854–23,632,158) | 1.25(0.8–1.9) | 5,357,450(4,231,439–6,519,671) | 0.4(0.05–0.94) | 499,255(222,091–917,208) | 25.03(12.87–49.9) | 170,88(7835–29,832) | -0.26(-0.42, -0.06) |
| East Asia | 575,501(309,988–1,027,198) | 17.56(8.42–5.29) | 4,3218(35,661–50,080) | 3.44(1.27–5.29) | 2237(381–6907) | 5.18(-0.23–5.29) | 196(33–605) | 0.03(-0.74, 5.29) |
| Southeast Asia | 1,549,666(1,209,139–2,060,608) | 1.75(1.55–40.06) | 93,848(83,549–104,118) | 4.62(3.63–40.06) | 3166(1615–5526) | 28.37(7.64–40.06) | 277(142–484) | -0.37(-0.69, 40.06) |
| Oceania | 60,649(43,722–84,376) | 43.09(30.58–4718.31) | 4095(3623–4566) | 66.85(44.19–4718.31) | 172(50–408) | 3064.26(788.12–4718.31) | 15(4–36) | 2.58(-0.18, 4718.31) |
| Central Asia | 71,184(56,829–94,354) | 2.52(2.44–191.96) | 890(694–1099) | -0.37(-0.59, 191.96) | 291(182–445) | 102.97(32.97–191.96) | 61(38–94) | -0.3(-0.49, 191.96) |
| Central Europe | 38,201(25,909–52,401) | -0.1(-0.14–1.08) | 476(412–551) | -0.63(-0.67, 1.08) | 10(5–20) | -0.01(-0.7, 1.08) | 2(1–4) | -0.29(-0.76, 1.08) |
| Eastern Europe | 1,459,394(1,141,975–1,807,561) | 4.37(4.24–27.18) | 5267(3764–6959) | -0.27(-0.58, 27.18) | 2507(1577–3672) | 17.76(6.72–27.18) | 527(332–772) | -0.4(-0.59, 27.18) |
| High-income Asia Pacific | 62,531(36,868–91,634) | 2.15(1.84–0.41) | 495(413–591) | 0.88(0.54–0.41) | 6(2–19) | 2.27(-0.21–0.41) | 1(0–2) | 0.03(-0.65, 0.41) |
| Australasia | 22,030(14,034–30,215) | -0.79(-0.82, 0.45) | 34(29–40) | -0.85(-0.88, 0.45) | 1(0–3) | -0.14(-0.78, 0.45) | 0(0–0) | 0.87(-0.48, 0.45) |
| Western Europe | 997,042(798,249–1,166,763) | -0.65(-0.69, -0.42) | 1590(1363–1848) | -0.88(-0.9, -0.42) | 40(25–63) | -0.65(-0.83, -0.42) | 5(3–8) | -0.12(-0.44, -0.42) |
| Southern Latin America | 229,508(195,308–270,809) | 2.1(2.01–3.31) | 3695(3189–4243) | 1.14(0.45–3.31) | 54(13–176) | 6.96(0.46–3.31) | 6(2–21) | 0.12(-0.67, 3.31) |
| High-income North America | 1,823,069(1,009,261–2,663,601) | -0.75(-0.79 , -0.88) | 950(802–1124) | -0.89(-0.9, -0.88) | 16(6–42) | -0.95(-0.98, -0.88) | 2(1–5) | 0.33(-0.45, -0.88) |
| Caribbean | 358,394(310,645–406,615) | -0.11(-0.33, 0.16) | 3962(3386–4598) | -0.57(-0.68, 0.16) | 22(9–51) | -0.35(-0.86, 0.16) | 2(1–4) | 0.15(-0.65, 0.16) |
| Andean Latin America | 147,211(115,558–183,852) | 5.94(5.39–11.49) | 3488(2938–4101) | 1.43(0.53–11.49) | 283(125–581) | 20.92(5.18–11.49) | 22(10–44) | -0.07(-0.5, 11.49) |
| Central Latin America | 529,405(398,838–684,145) | 1.57(1.53–28.86) | 9454(8190–10,881) | -0.17(-0.34, 28.86) | 334(136–693) | 18.43(6.11–28.86) | 25(10–53) | 0.36(-0.29, 28.86) |
| Tropical Latin America | 705,505(451,488–992,673) | 0.69(0.64–64.45) | 15,172(12,930–17,615) | -0.26(-0.43, 64.45) | 545(120–1563) | 47.01(5.65–64.45) | 41(9–119) | 0.57(-0.56, 64.45) |
| North Africa and Middle East | 229,286(142,519–406,822) | 12.81(4.9–44.55) | 5916(5149–6779) | 2.07(1.04–44.55) | 158(88–278) | 34.24(12.22–44.55) | 5(3–10) | 0.3(-0.32, 44.55) |
| South Asia | 2,110,424(1,770,014–2,573,655) | 130.88(67.82–1111.12) | 10,5791(88,304–123,954) | 54.32(29.16–1111.12) | 8826(2377–21,121) | 5069.67(712.4–1111.12) | 209(56–500) | -0.46(-0.82, 1111.12) |
| Central Sub-Saharan Africa | 1,237,893(1,047,503–1,434,515) | 0.77(0.23–8.82) | 97,431(86,636–108,994) | 0.01(-0.31, 8.82) | 2489(926–5600) | 7.3(0.82–8.82) | 16(6–35) | -0.29(-0.72, 8.82) |
| Eastern Sub-Saharan Africa | 11,480,329(10,637,773–12,631,901) | 0.28(-0.01, 55.56) | 522,225(453,808–590,926) | -0.13(-0.38, 55.56) | 21,595(12,291–36,375) | 55(19.55–55.56) | 135(77–227) | 0.07(-0.37, 55.56) |
| Southern Sub-Saharan Africa | 11,549,270(11,064,897–12,052,321) | 4.04(2.74–39.74) | 530,816(469,127–602,263) | 2.97(1.91–39.74) | 20033(9192–42,753) | 48.48(10.71–39.74) | 125(57–267) | -0.33(-0.71, 39.74) |
| Western Sub-Saharan Africa | 4,800,443(4,517,421–5,079,614) | 2.49(1.7–10.68) | 233,301(203,029–263,919) | 0.76(0.29–10.68) | 8671(4121–17,157) | 9.35(2.58–10.68) | 54(26–107) | -0.23(-0.6, 10.68) |
| High-middle SDI | 2,410,263(1,982,432–2,874,836) | 2.42(2.2–17.42) | 25,894(22,233–29,755) | 0.08(-0.08, 17.42) | 3261(2103–5154) | 11.05(5.27–17.42) | 591(381–872) | -0.39(-0.56, 17.42) |
| High SDI | 2,538,148(1,581,354–3,510,854) | -0.68(-0.72, -0.39) | 4041(3487–4669) | -0.83(-0.85, -0.39) | 129(83–213) | -0.75(-0.87, -0.39) | 16(11–25) | -0.18(-0.4, -0.39) |
| Low-middle SDI | 9,876,631(9,372,625–10,386,889) | 1.98(1.39–34.94) | 453,520(399,294–518,011) | 0.63(0.16–34.94) | 20,919(1,2369–32,076) | 34.24(11.13–34.94) | 377(217–622) | -0.39(-0.6, 34.94) |
| Low SDI | 11,499,846(10,632,720–12,671,653) | 0.27(0–21.27) | 596,879(523,034–669,614) | -0.13(-0.35, 21.27) | 24,483(14,805–38,104) | 18.74(7.33–21.27) | 192(117–296) | 0.01(-0.36, 21.27) |
| Middle SDI | 13,683,296(12,746,460–14,849,390) | 6.33(5.79–35.69) | 600,861(536,310–673,728) | 5.53(4.61–35.69) | 22,632(11,424–45,654) | 51.08(18.91–35.69) | 550(331–914) | -0.2(-0.5, 35.69) |

Notes: Globally, the World Health Organization began to recommend the XDR-TB surveillance in 1991. Consequently, the age-standardized incidence rate of HIV-XDR-TB has been tracked and reported since 1991. However, the GBD 2021 database provides total percentage change data for the periods 1990－2000, 2000－2021, 1990－2021, 2010－2021, and 2019－2021. Therefore, percentage change of DALY number cases for HIV-XDR-TB of 2010－2021 were used in the study. DALYs: disability-adjusted life years. HIV-DS-TB: HIV-infected drug-susceptible tuberculosis. HIV-MDR-TB: HIV-infected multidrug-resistant tuberculosis without extensive drug resistance. HIV-XDR-TB: HIV-infected extensively drug-resistant tuberculosis.

Table S6 Predicted age-standardized rates of HIV-DS-TB, HIV-MDR-TB, and HIV-XDR-TB spanning 2022－2035, based on the Bayesian Age-Period-Cohort Model.

| Year | HIV-DS-TB | | | HIV-MDR-TB | | |  | HIV-XDR-TB | |
| --- | --- | --- | --- | --- | --- | --- | --- | --- | --- |
|  | ASIR (per 100,000 population)  (95% *CI*) | ASPR (per 100,000 population)  (95% *CI*) | ASMR (per 100,000 population)  (95% *CI*) | ASIR (per 100,000 population)  (95% *CI)* | ASPR (per 100,000 population)  (95% *CI*) | ASMR (per 100,000 population)  (95% *CI*) | ASIR (per 100,000 population)  (95% *CI*) | ASPR (per 100,000 population)  (95% *CI*) | ASMR (per 100,000 population)  (95% *CI*) |
| 2022 | 13.69(9.8–17.58) | 24.19(17.42–30.96) | 2.53(1.84–3.22) | 0.69(0.39–0.98) | 1.09(0.6–1.57) | 0.25(0.14–0.37) | 0.02(0.01–0.03) | 0.02(0.01–0.03) | 0.01(0.00–0.02) |
| 2023 | 14.02(9.21–18.83) | 24.76(16.46–33.06) | 2.59(1.66–3.51) | 0.76(0.31–1.20) | 1.20(0.47–1.94) | 0.28(0.11–0.45) | 0.03(0.01–0.04) | 0.03(0.01–0.05) | 0.01(0.00–0.03) |
| 2024 | 14.35(8.67–20.03) | 25.34(15.57–35.11) | 2.65(1.51–3.79) | 0.83(0.23–1.43) | 1.33(0.33–2.33) | 0.31(0.08–0.55) | 0.03(0.00–0.06) | 0.03(0.01–0.06) | 0.02(0.00–0.04) |
| 2025 | 14.69(8.16–21.23) | 25.94(14.73–37.15) | 2.72(1.38–4.06) | 0.92(0.15–1.69) | 1.47(0.19–2.76) | 0.34(0.04–0.65) | 0.04(0.00–0.07) | 0.04(0.00–0.07) | 0.02(0.00–0.05) |
| 2026 | 15.04(7.66–22.42) | 26.55(13.90–39.19) | 2.79(1.25–4.34) | 1.01(0.05–1.97) | 1.63(0.02–3.24) | 0.38(0.00–0.76) | 0.04(-0.01–0.09) | 0.04(0.00–0.09) | 0.02(0.00–0.06) |
| 2027 | 15.38(7.15–23.62) | 27.16(13.07–41.24) | 2.87(1.12–4.62) | 1.12(0.00–2.29) | 1.81(0.00–3.78) | 0.42(0.00–0.89) | 0.05(-0.01–0.11) | 0.05(0.00–0.11) | 0.03(0.00–0.08) |
| 2028 | 15.71(6.63–24.79) | 27.73(12.22–43.25) | 2.95(1.00–4.91) | 1.23(0.00–2.64) | 2.00(0.00–4.39) | 0.47(0.00–1.04) | 0.06(-0.02–0.13) | 0.06(0.00–0.13) | 0.03(0.00–0.09) |
| 2029 | 16.01(6.10–25.92) | 28.26(11.33–45.18) | 3.03(0.87–5.20) | 1.35(0.00–3.02) | 2.21(0.00–5.06) | 0.52(0.00–1.21) | 0.07(-0.03–0.16) | 0.07(0.00–0.16) | 0.04(0.00–0.12) |
| 2030 | 16.27(5.54–27.00) | 28.73(10.41–47.04) | 3.11(0.73–5.49) | 1.48(0.00–3.45) | 2.43(0.00–5.80) | 0.58(0.00–1.40) | 0.08(-0.05–0.2) | 0.08(0.00–0.19) | 0.05(0.00–0.15) |
| 2031 | 16.51(4.97–28.05) | 29.14(9.46–48.83) | 3.19(0.59–5.78) | 1.62(0.00–3.93) | 2.68(0.00–6.62) | 0.65(0.00–1.61) | 0.09(-0.06–0.24) | 0.09(0.00–0.23) | 0.06(0.00–0.19) |
| 2032 | 16.71(4.39–29.04) | 29.50(8.49–50.52) | 3.26(0.45–6.07) | 1.78(0.00–4.45) | 2.94(0.00–7.53) | 0.71(0.00–1.85) | 0.1(-0.08–0.29) | 0.11(0.00–0.28) | 0.07(0.00–0.23) |
| 2033 | 16.87(3.78–29.95) | 29.77(7.48–52.06) | 3.33(0.3–6.36) | 1.94(0.00–5.01) | 3.22(0.00–8.53) | 0.79(0.00–2.11) | 0.12(-0.11–0.35) | 0.12(0.00–0.33) | 0.08(0.00–0.28) |
| 2034 | 16.96(3.17–30.75) | 29.93(6.44–53.41) | 3.39(0.15–6.63) | 2.11(0.00–5.62) | 3.51(0.00–9.60) | 0.87(0.00–2.41) | 0.14(-0.14–0.42) | 0.14(0.00–0.40) | 0.10(0.00–0.35) |
| 2035 | 17.00(2.55–31.45) | 29.98(5.40–54.57) | 3.44(0.00–6.88) | 2.28(0.00–6.28) | 3.820.00–10.76) | 0.96(0.00–2.73) | 0.16(-0.18–0.5) | 0.16(0.00–0.47) | 0.12(0.00–0.43) |

Notes: Globally, the World Health Organization began to recommend XDR-TB surveillance in 1991. Consequently, the age-standardized incidence rate and prevalence rate of HIV-XDR-TB have been tracked and reported since 1991, and the age-standardized mortality rate has been tracked and reported since 1993. When the ASRs is predicted for a given year, if the lower limits of the 95% confidence intervals is below 0, 0 is set. ASRs: age-standardized rates. CI: Confidence interval. EAPC: annual percentage changes. HIV-DS-TB: HIV-infected drug-susceptible tuberculosis. HIV-MDR-TB: HIV-infected. multidrug-resistant tuberculosis without extensive drug resistance. HIV-XDR-TB: HIV-infected extensively drug-resistant tuberculosis.


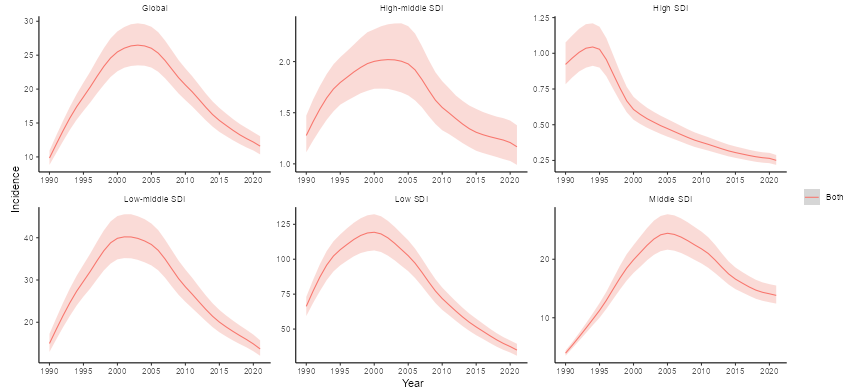


A


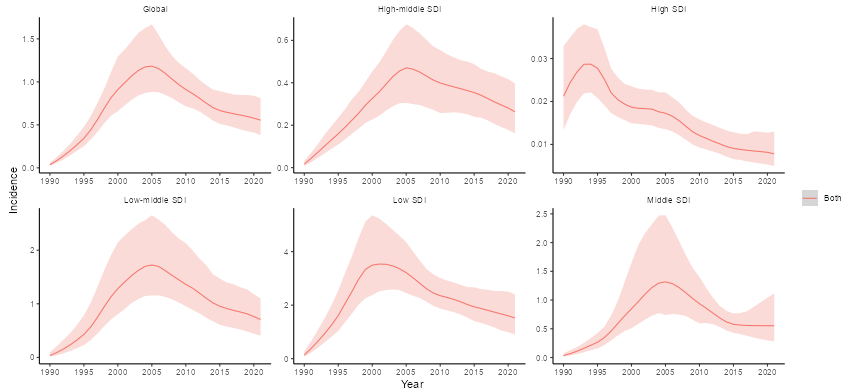


B


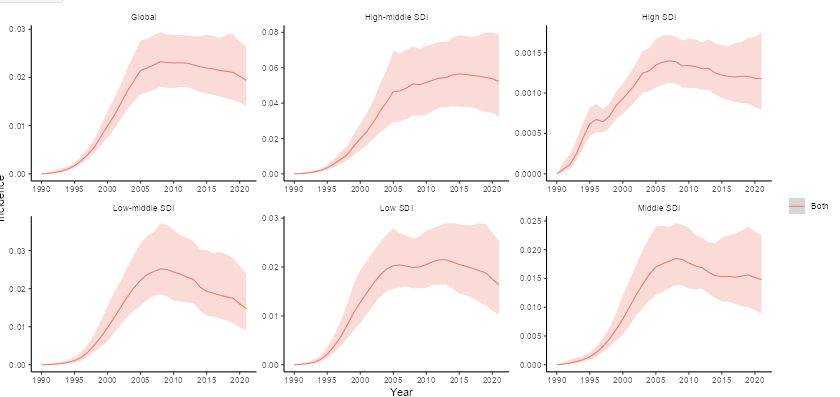


C

Fig. S1 The trends in the age-standardized incidence rate for HIV-DS-TB, HIV-MDR-TB, HIV-XDR-TB varied across the five SDI regions (A: HIV-DS-TB. B: HIV-MDR-TB. C: HIV-XDR-TB. HIV-DS-TB: HIV-infected drug-susceptible tuberculosis. HIV-MDR-TB: HIV-infected multidrug-resistant tuberculosis without extensive drug resistance. HIV-XDR-TB: HIV-infected extensively drug-resistant tuberculosis).


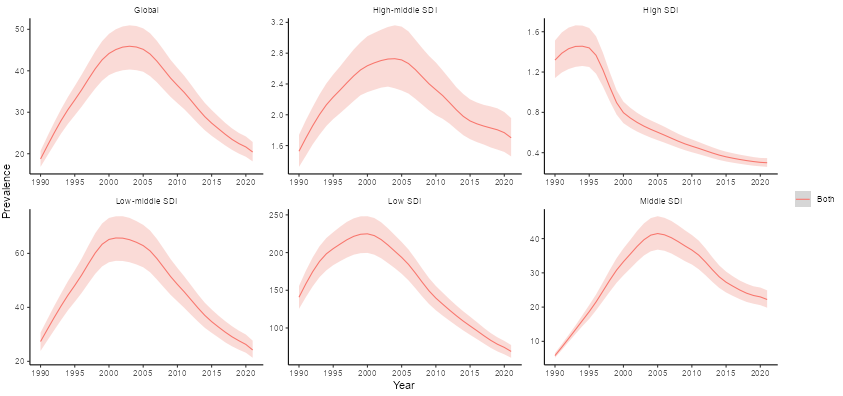


A


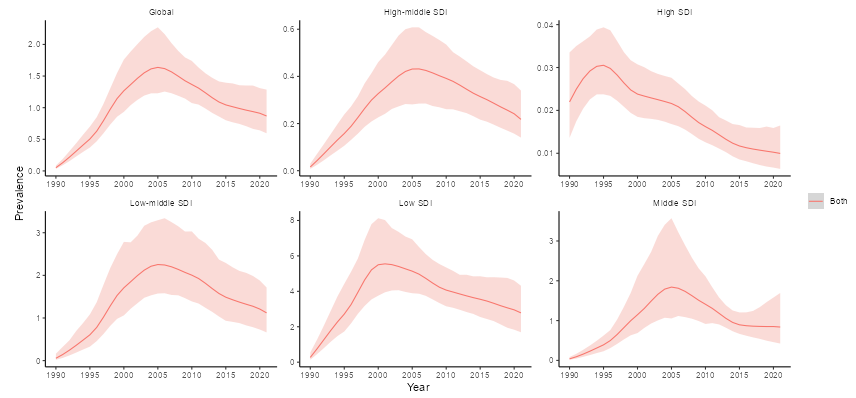


B


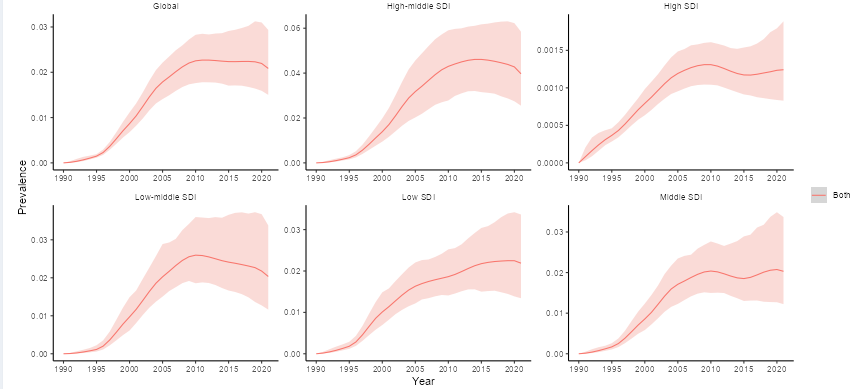


C

Fig. S2 The trends in the age-standardized prevalence rate for HIV-DS-TB, HIV-MDR-TB, HIV-XDR-TB varied across the five SDI regions( A: HIV-DS-TB. B: HIV-MDR-TB. C: HIV-XDR-TB. HIV-DS-TB: HIV-infected drug-susceptible tuberculosis. HIV-MDR-TB: HIV-infected multidrug-resistant tuberculosis without extensive drug resistance. HIV-XDR-TB: HIV-infected extensively drug-resistant tuberculosis).


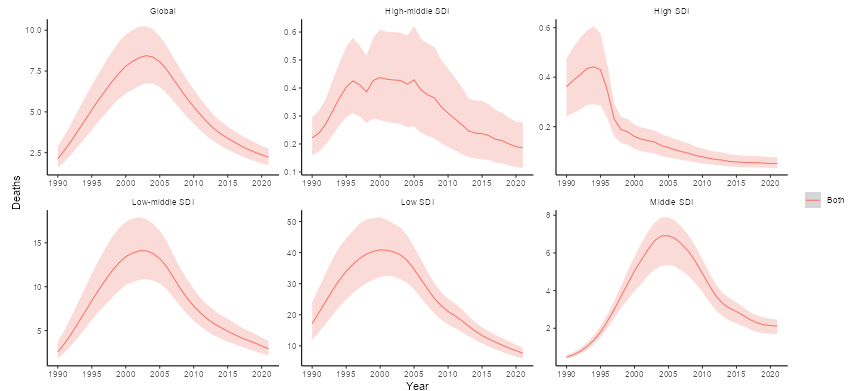


A


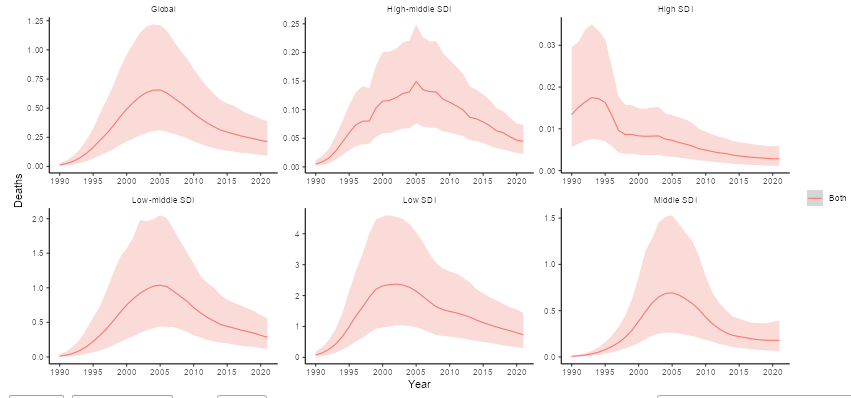


B

C


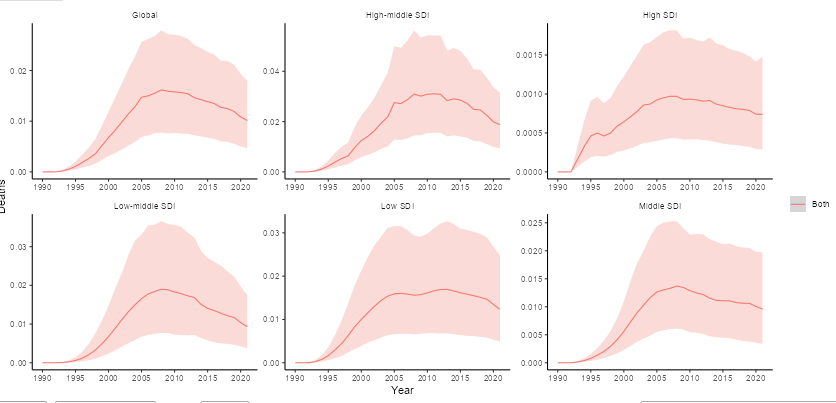


Fig. S3 The trends in the age-standardized mortality rate for HIV-DS-TB, HIV-MDR-TB, HIV-XDR-TB varied across the five SDI regions (A: HIV-DS-TB. B: HIV-MDR-TB. C: HIV-XDR-TB. HIV-infected drug-susceptible tuberculosis. HIV-MDR-TB: HIV-infected multidrug-resistant tuberculosis without extensive drug resistance. HIV-XDR-TB: HIV-infected extensively drug-resistant tuberculosis).


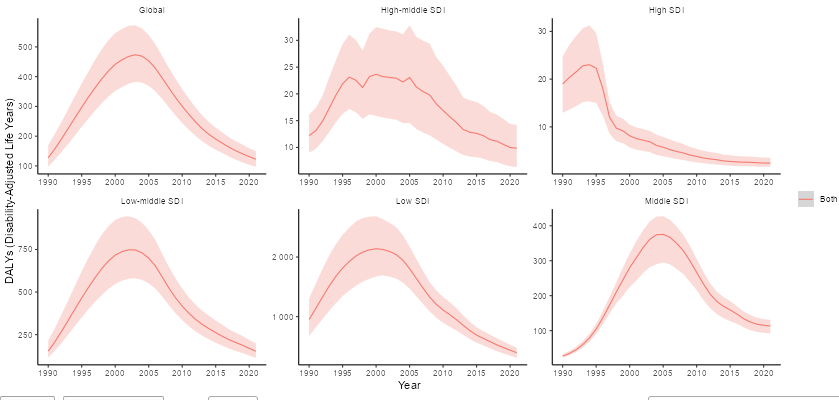


A


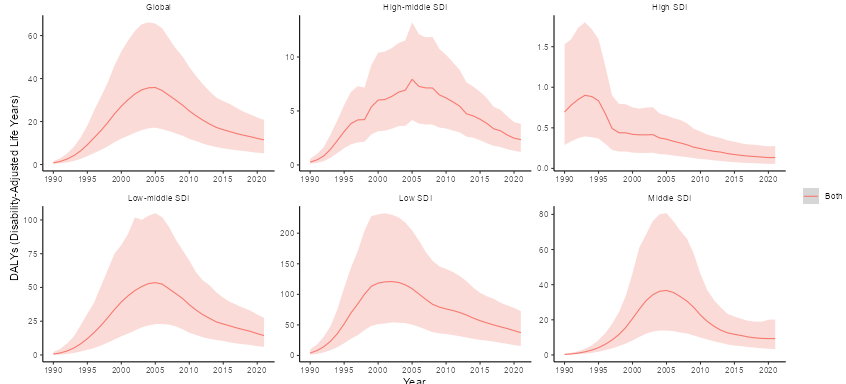


B


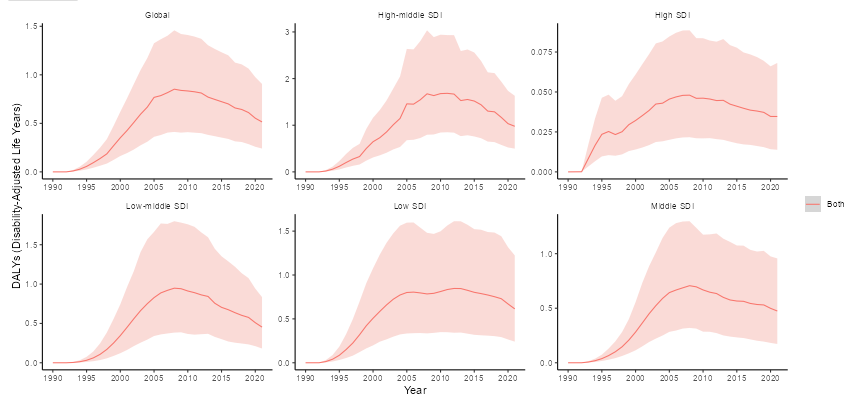


C

Fig. S4 The trends in the age-standardized DALY rates for HIV-DS-TB, HIV-MDR-TB, HIV-XDR-TB varied across the five SDI regions (A: HIV-DS-TB. B: HIV-MDR-TB. C: HIV-XDR-TB. HIV-DS-TB: HIV-infected drug-susceptible tuberculosis. HIV-MDR-TB: HIV-infected multidrug-resistant tuberculosis without extensive drug resistance. HIV-XDR-TB: HIV-infected extensively drug-resistant tuberculosis).


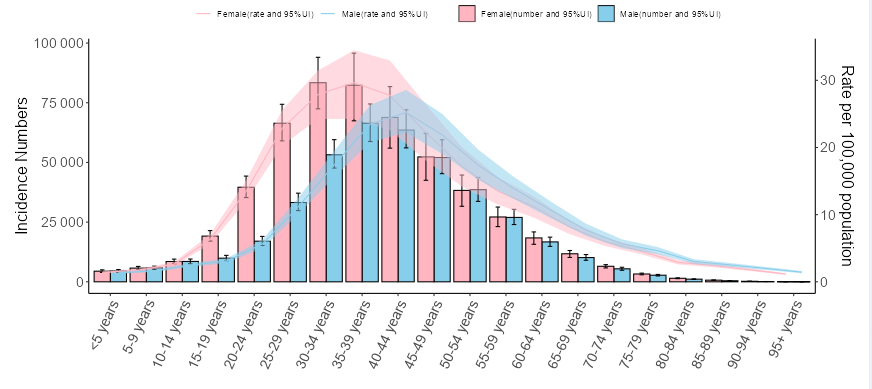


A


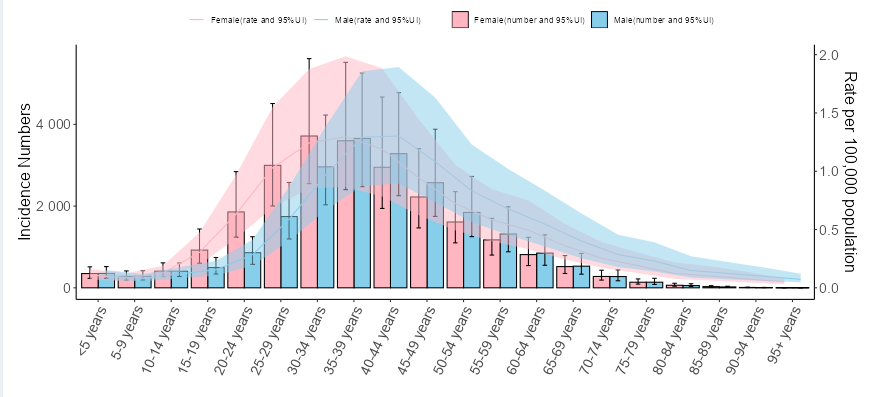


B


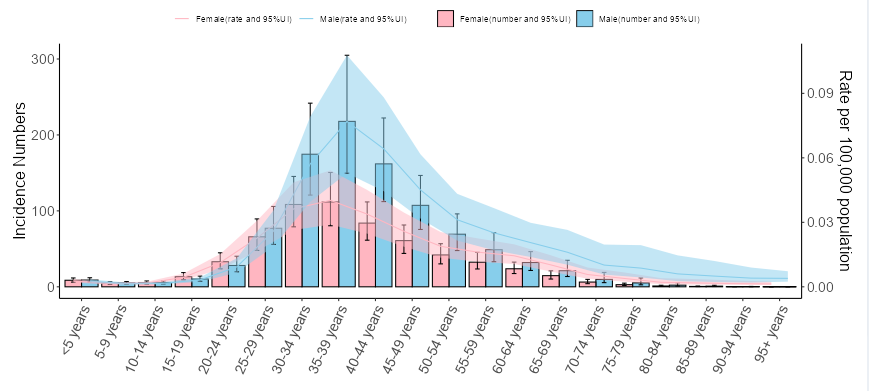


C

Fig. S5 The specific incidence rate of HIV-DS-TB, HIV-MDR-TB, and HIV-XDR-TB showed notable differences across age and gender distributions in 2021 year (A: Incidence rate of HIV-DS-TB. B: Incidence rate of HIV-MDR-TB. C: Incidence rate of HIV-XDR-TB. HIV-DS-TB: HIV-infected drug-susceptible tuberculosis. HIV-MDR-TB: HIV-infected multidrug-resistant tuberculosis without extensive drug resistance. HIV-XDR-TB: HIV-infected extensively drug-resistant tuberculosis).


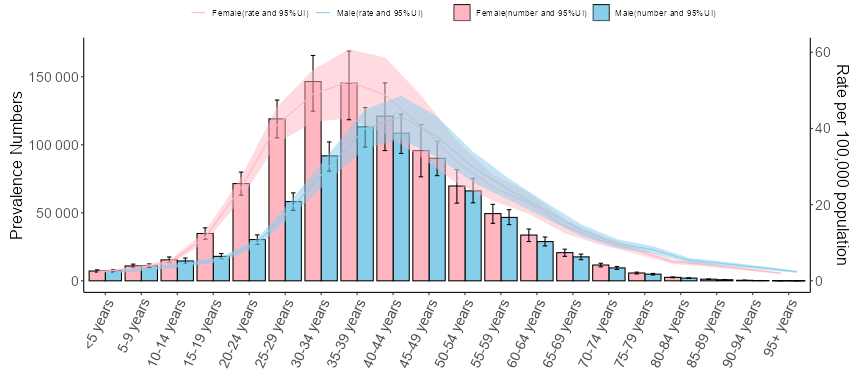


A


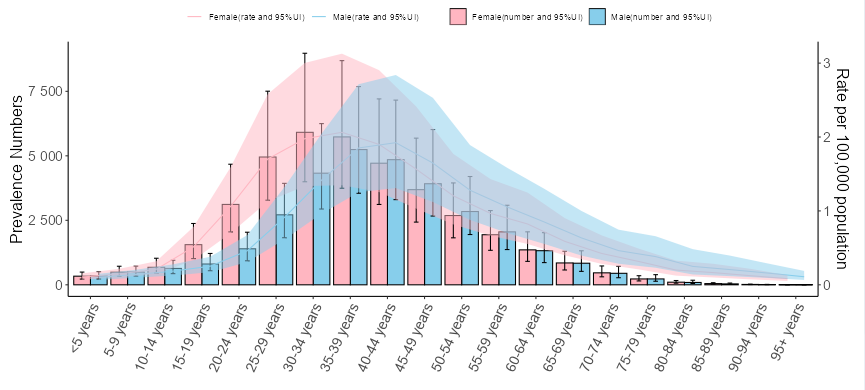


B


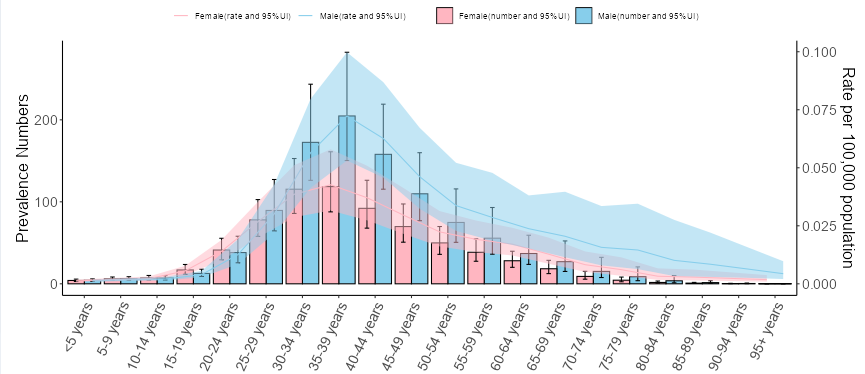


C

Fig. S6 The specific prevalence rate of HIV-DS-TB, HIV-MDR-TB, and HIV-XDR-TB showed notable differences across age and gender distributions in 2021 year ( A: Prevalence rate of HIV-DS-TB. B: Prevalence rate of HIV-MDR-TB. C: Prevalence rate of HIV-XDR-TB. HIV-DS-TB: HIV-infected drug-susceptible tuberculosis. HIV-MDR-TB: HIV-infected multidrug-resistant tuberculosis without extensive drug resistance. HIV-XDR-TB: HIV-infected extensively drug-resistant tuberculosis).


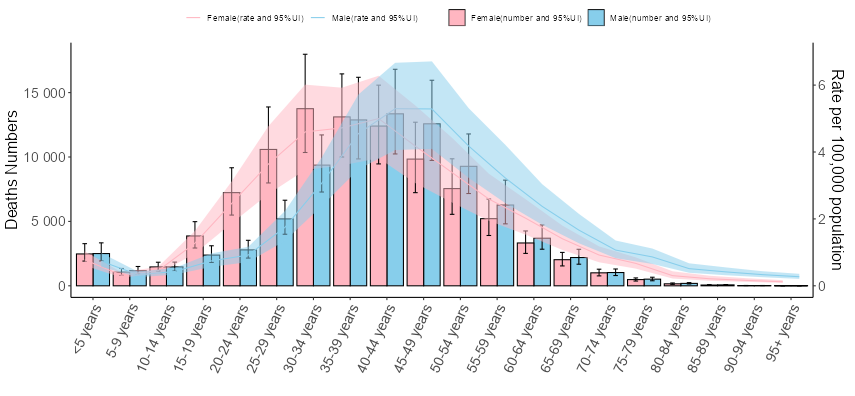


A


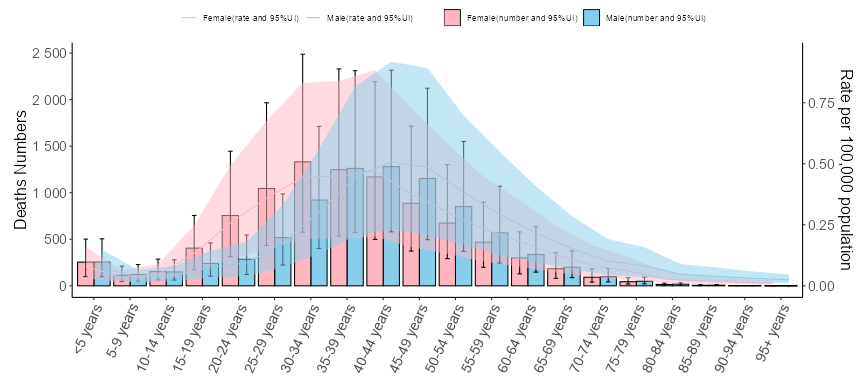


B


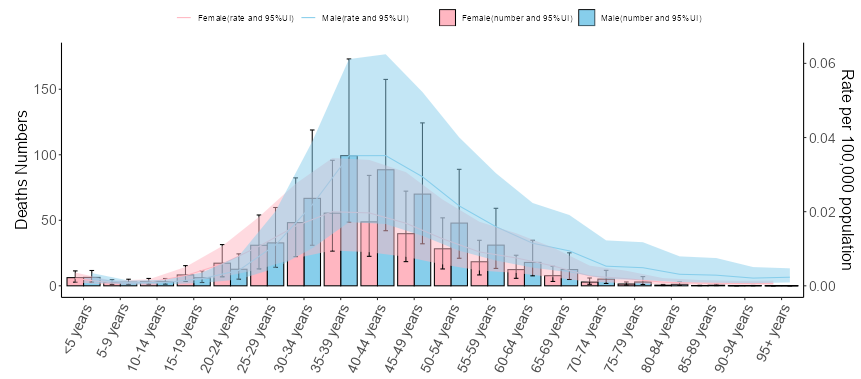


C

Fig. S7 The specific mortality rate of HIV-DS-TB, HIV-MDR-TB, and HIV-XDR-TB showed notable differences across age and gender distributions in 2021 year ( A: Mortality rate of HIV-DS-TB. B: Mortality rate of HIV-MDR-TB. C: Mortality rate of HIV-XDR-TB. HIV-DS-TB: HIV-infected drug-susceptible tuberculosis. HIV-MDR-TB: HIV-infected multidrug-resistant tuberculosis without extensive drug resistance. HIV-XDR-TB: HIV-infected extensively drug-resistant tuberculosis).


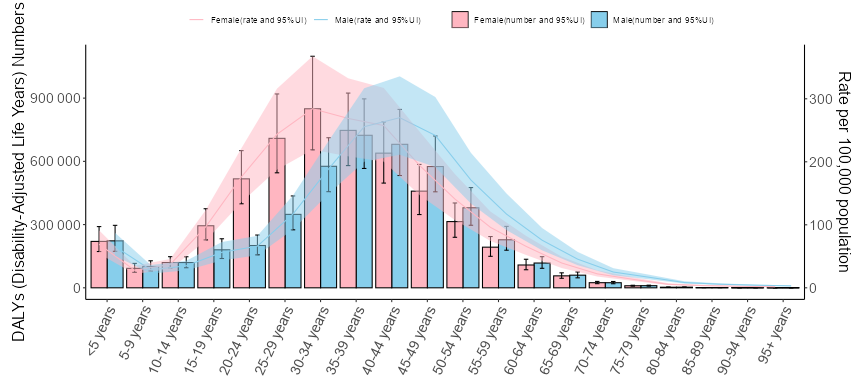


A


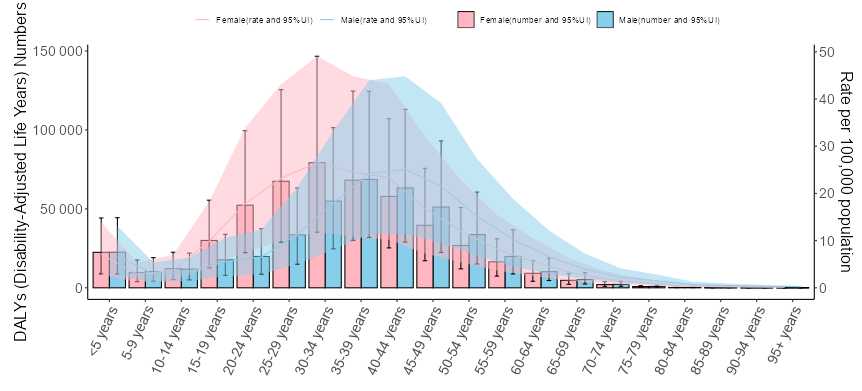


B


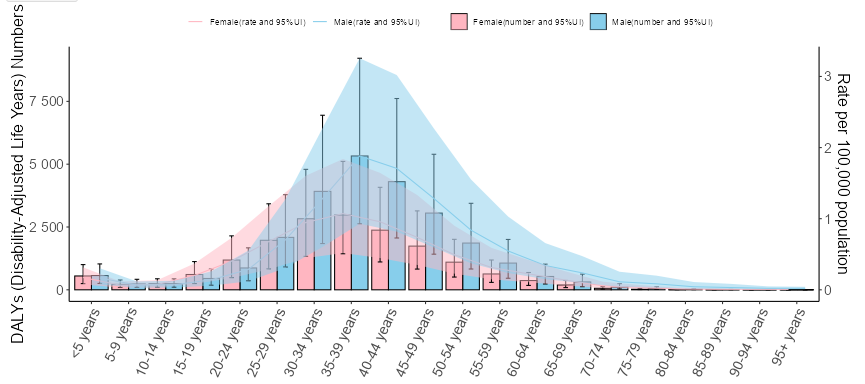


C

Fig. S8 The specific age-standardized DALY rate of HIV-DS-TB, HIV-MDR-TB, and HIV-XDR-TB showed notable differences across age and gender distributions in 2021 year ( A: Age-standardized DALY rate of HIV-DS-TB. B: Age-standardized DALY rate of HIV-MDR-TB. C: Age-standardized DALY rate of HIV-XDR-TB. DALY: disability-adjusted life years. HIV-DS-TB: HIV-infected drug-susceptible tuberculosis. HIV-MDR-TB: HIV-infected multidrug-resistant tuberculosis without extensive drug resistance. HIV-XDR-TB: HIV-infected extensively drug-resistant tuberculosis).


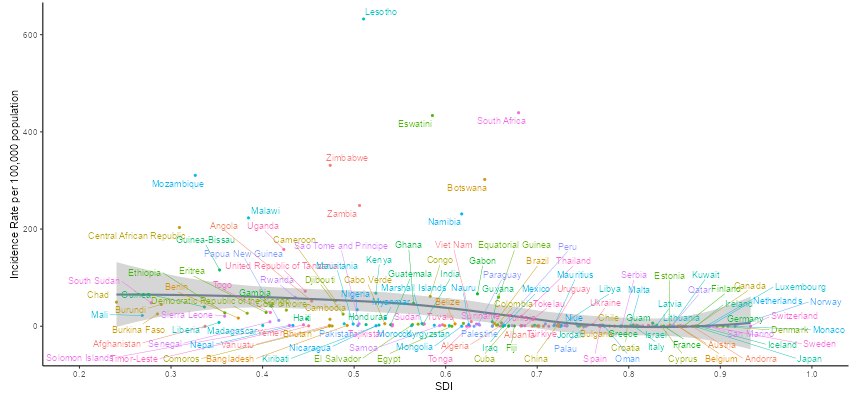

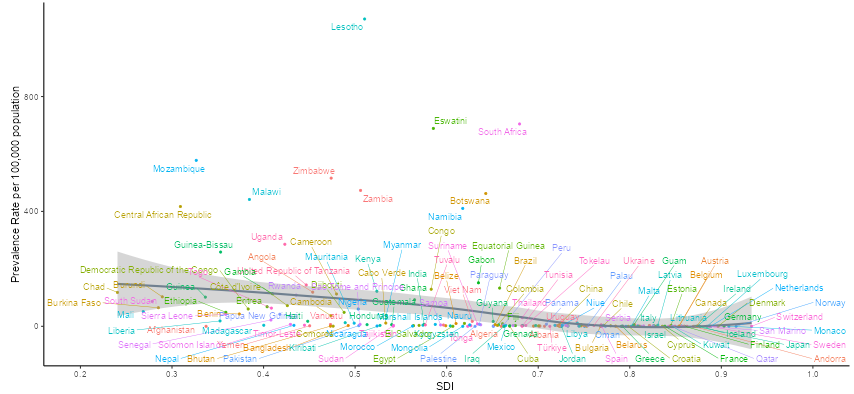


A

B


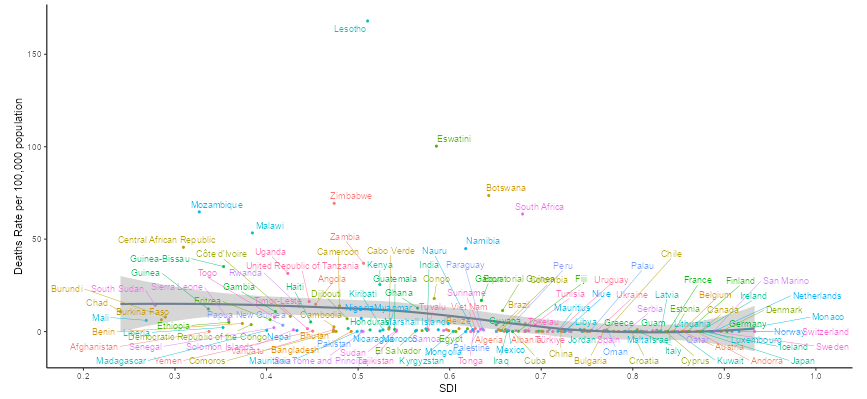

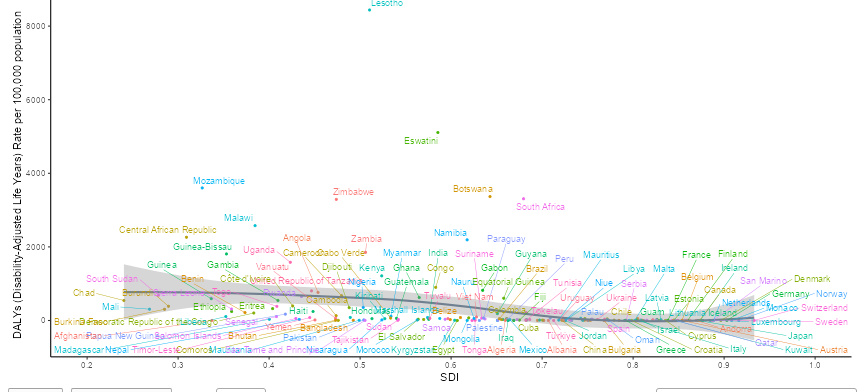


D

C

Fig. S9 The association between the SDI and the age-standardized incidence rate, mortality rate, and DALY rate of HIV-DS-TB across 204 countries and regions in 2021 year (A: Incidence rate of HIV-DS-TB. B: Prevalence rate of HIV-DS-TB. C: mortality rate of HIV-DS-TB. D: DALY rate of HIV-DS-TB. DALYs: disability-adjusted life years. HIV-DS-TB: HIV-infected drug-susceptible tuberculosis. SDI: sociodemographic index).


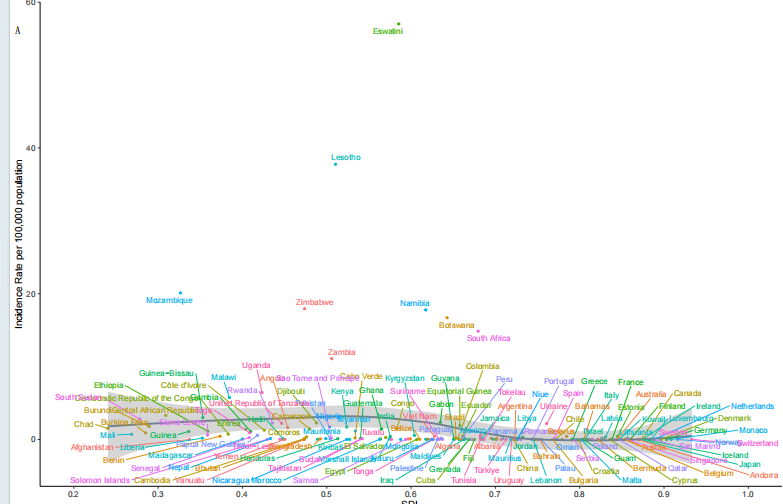

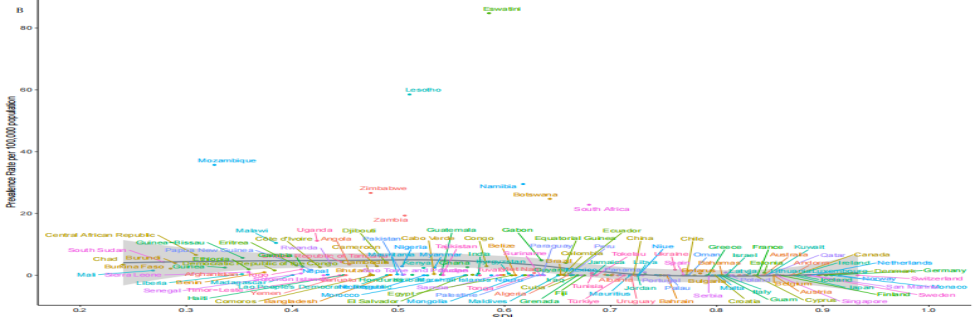


B

A


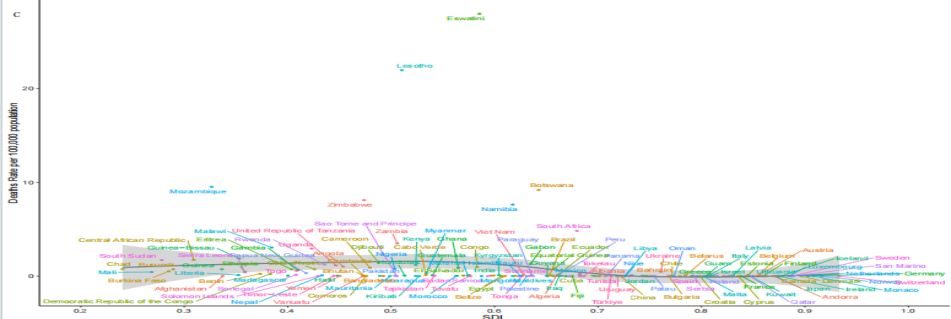

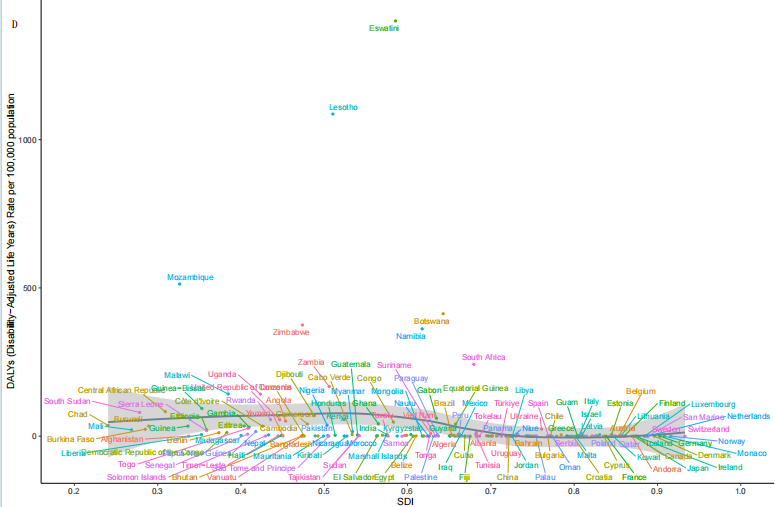


D

C

Fig. S10 The association between the SDI and the age-standardized incidence rate, death rate, and DALY rate of HIV-MDR-TB across 204 countries and regions in 2021 year (A: Incidence rate of HIV-MDR-TB. B: Prevalence rate of HIV-MDR-TB. C: Mortality rate of HIV-MDR-TB. D: DALY rate of HIV-MDR-TB. DALYs: disability-adjusted life years. HIV-MDR-TB: HIV-infected multidrug-resistant tuberculosis without extensive drug resistance. SDI: sociodemographic index).


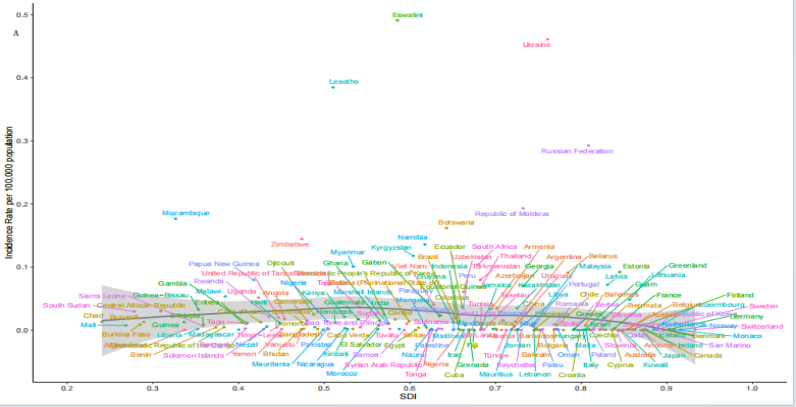

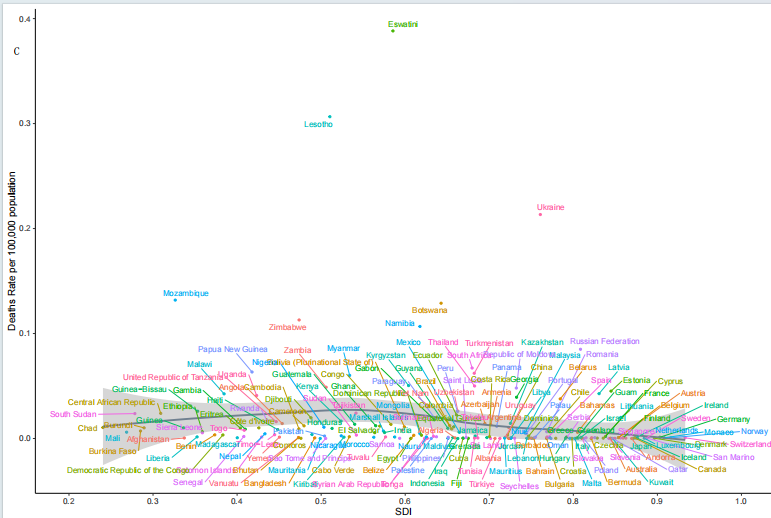


B

A


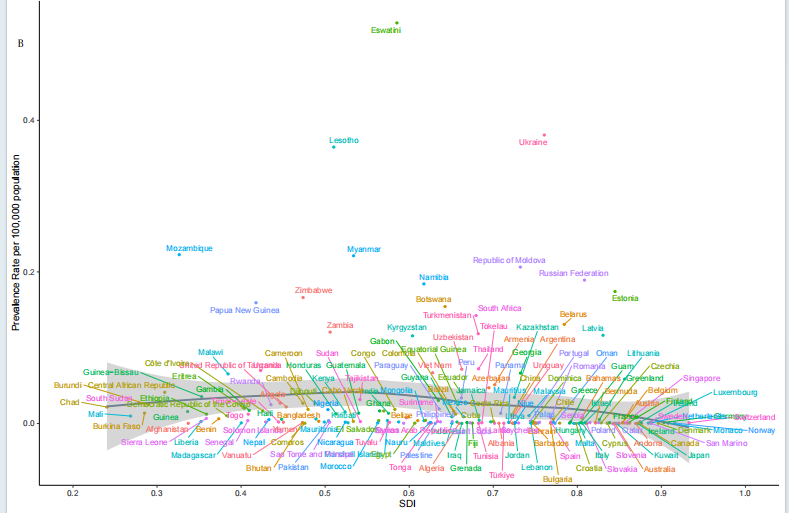

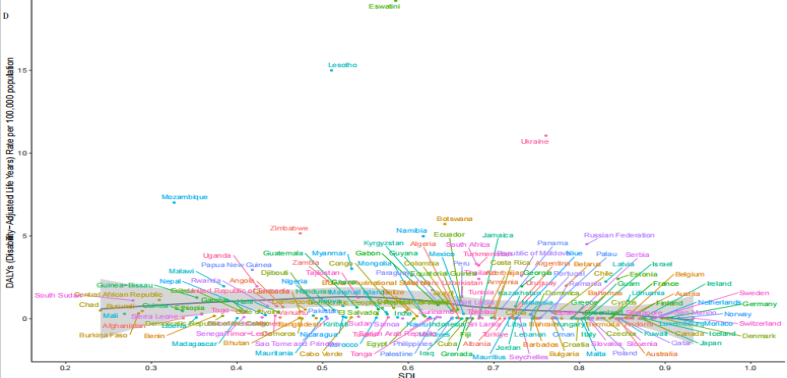


D

C

Fig.S11 The association between the SDI and the age-standardized incidence rate, death rate, and DALY rate of HIV-XDR-TB across 204 countries and regions in 2021 year (A: Incidence rate of HIV-XDR-TB. B: Prevalence rate of HIV-XDR-TB. C: Mortality rate of HIV-XDR-TB. D: DALY rate of HIV-XDR-TB. DALYs: disability-adjusted life years. HIV-XDR-TB: HIV-infected extensively drug-resistant tuberculosis. SDI: sociodemographic index ).


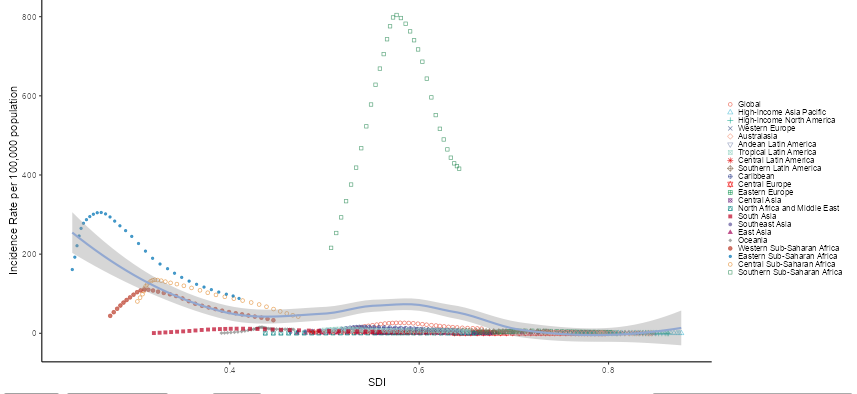

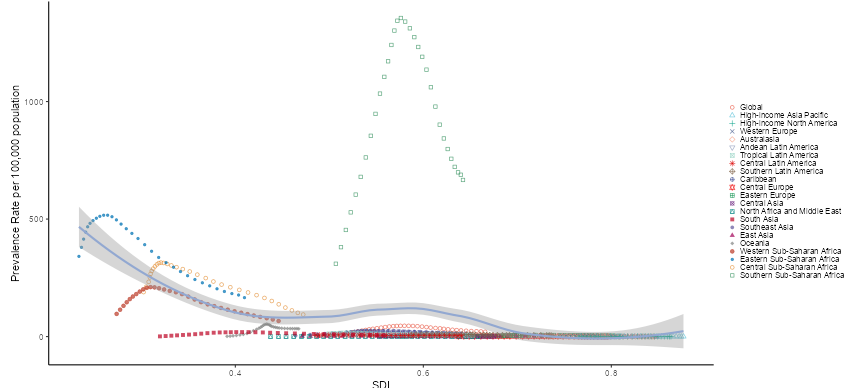

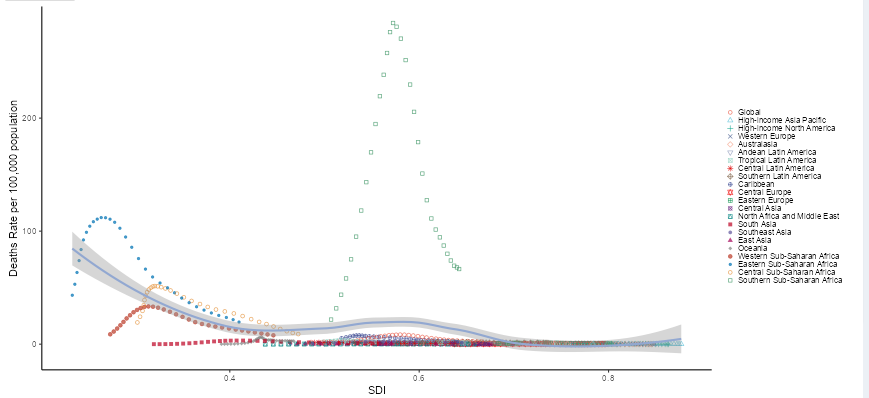

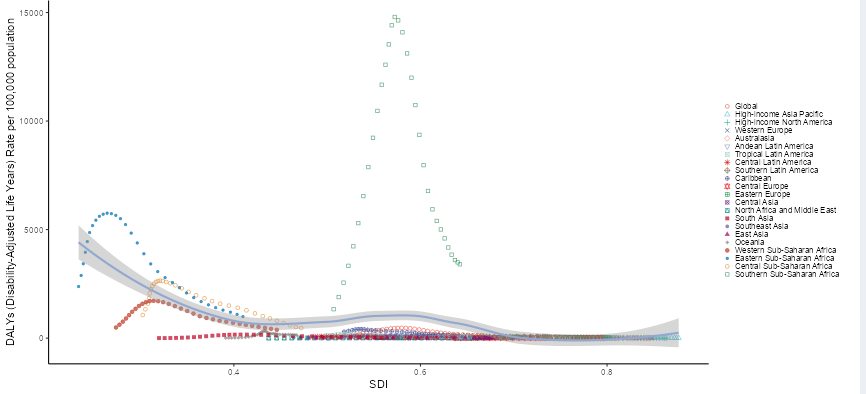


D

C

B

A

Fig. S12 The association between the age-standardized incidence rate, prevalence rate, mortality rate, and DALY rate of HIV-DS-TB with the SDI from 1990 to 2021 year (A: Incidence rate of HIV-DS-TB. B: Prevalence rate of HIV-DS-TB . C: Mortality rate of HIV-DS-TB . D: DALY rate of HIV-DS-TB. DALYs: disability-adjusted life years. HIV-DS-TB: HIV-infected drug-susceptible tuberculosis. SDI:sociodemographic index ).


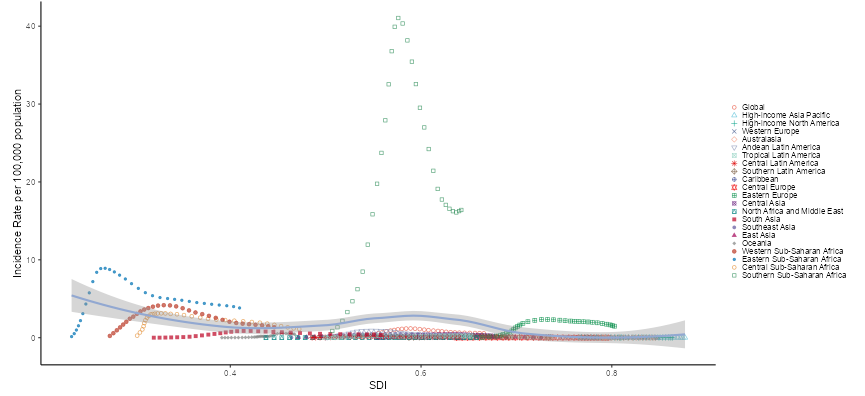

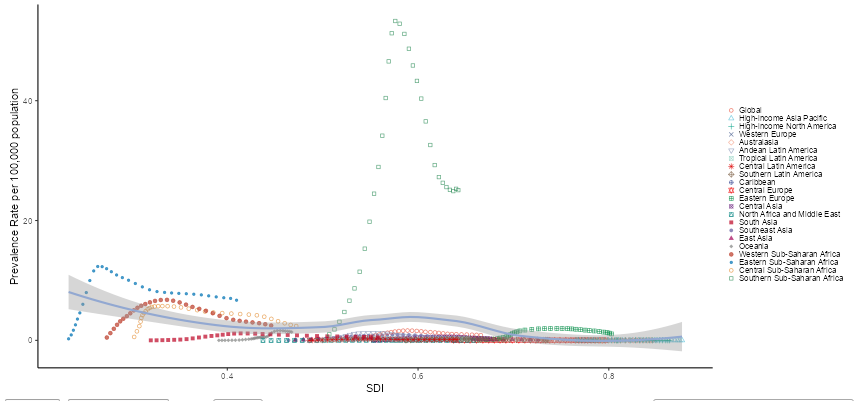


B

A

C

D


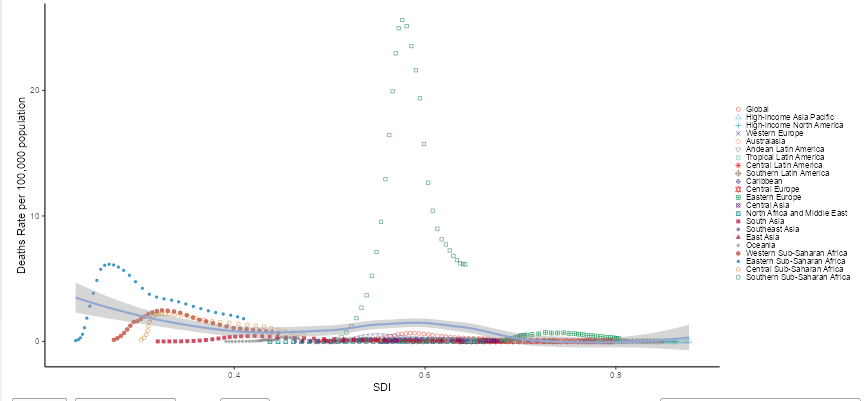

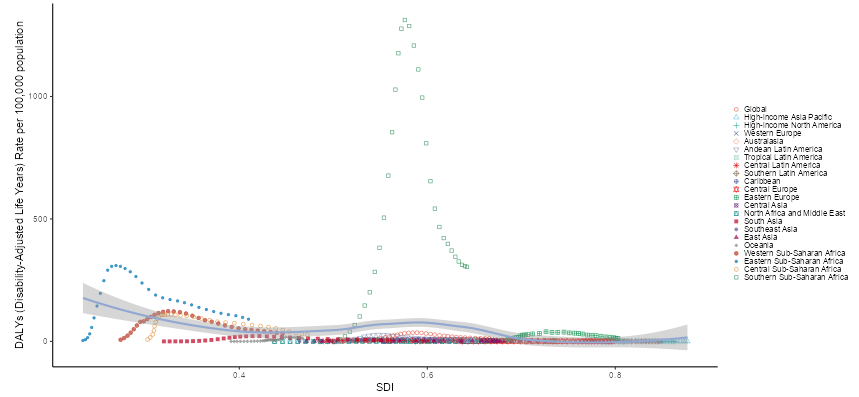


Fig. S13 The association between the age-standardized incidence rate, prevalence rate, mortality rate, and DALY rate of HIV-MDR-TB with the SDI from 1990 to 2021 year (A: Incidence rate of HIV-MDR-TB. B: Prevalence rate of HIV-MDR-TB . C: Mortality rate of HIV-MDR-TB . D: DALY rate of HIV-MDR-TB. DALYs: disability-adjusted life years. HIV-MDR-TB: HIV-infected multidrug-resistant tuberculosis without extensive drug resistance. SDI: Sociodemographic index).


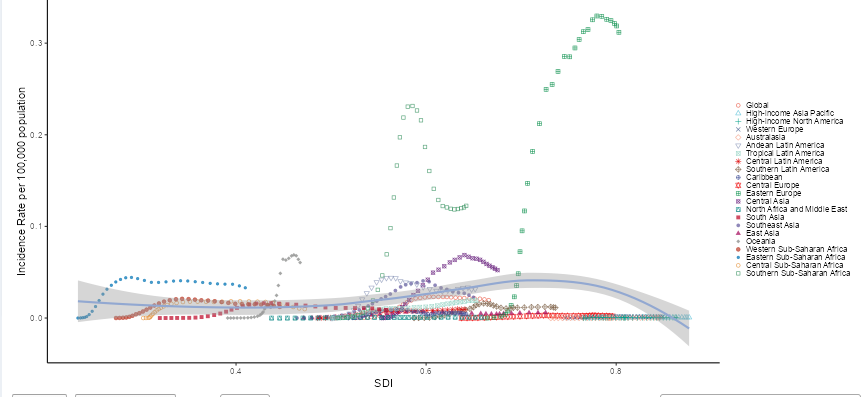

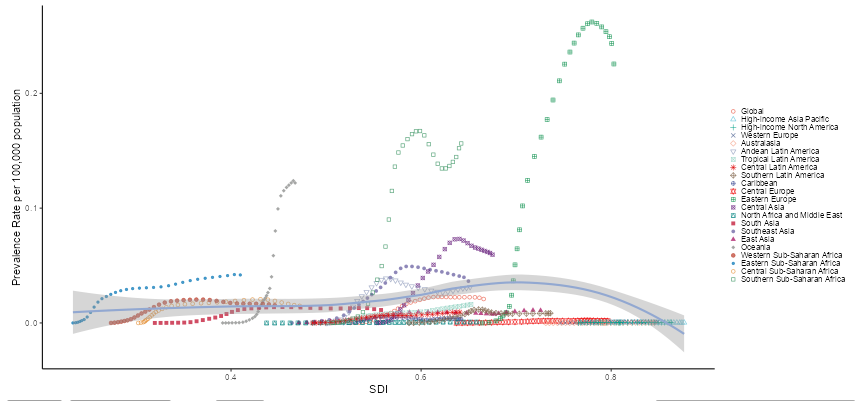


A

B


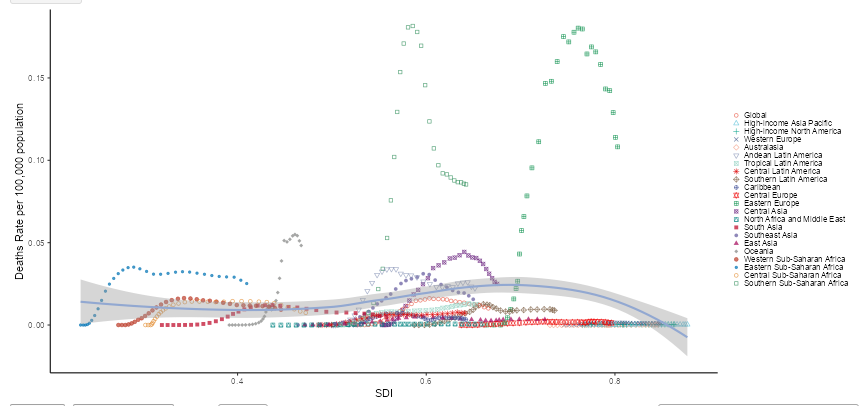

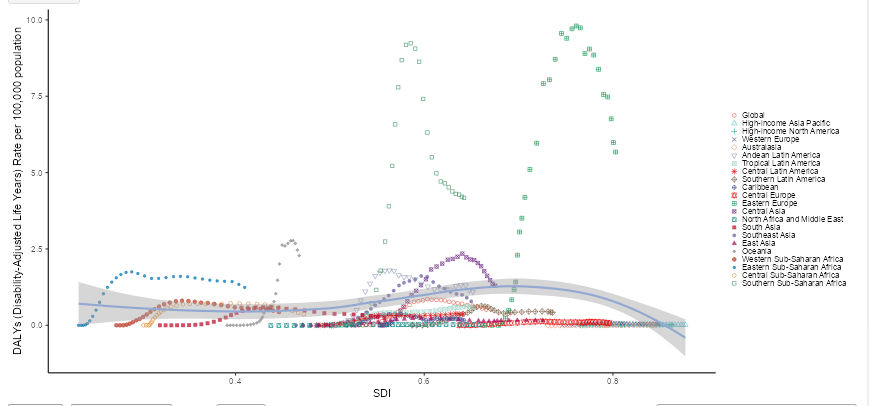


D

C

Fig. S14 The association between the age-standardized incidence rate, prevalence rate, mortality rate, and DALY rate of HIV-XDR-TB with the SDI from 1990 to 2021 year (A: Incidence rate of HIV-XDR-TB. B: Prevalence rate of HIV-XDR-TB . C: Mortality rate of HIV-XDR-TB. D: DALY rate of HIV-XDR-TB. DALYs: disability-adjusted life years. HIV-XDR-TB: HIV-infected extensively drug-resistant tuberculosis. SDI: sociodemographic index).


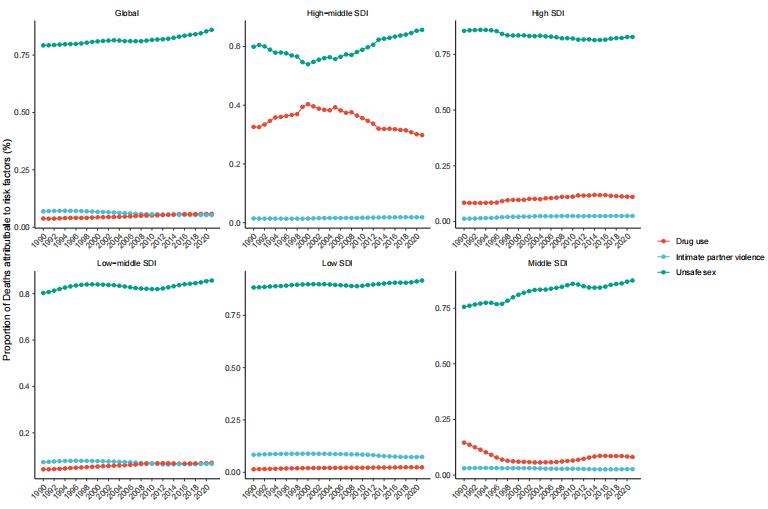

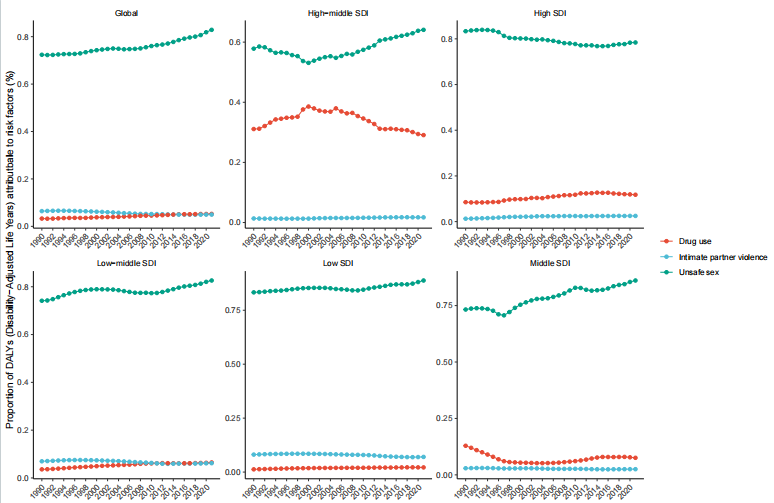

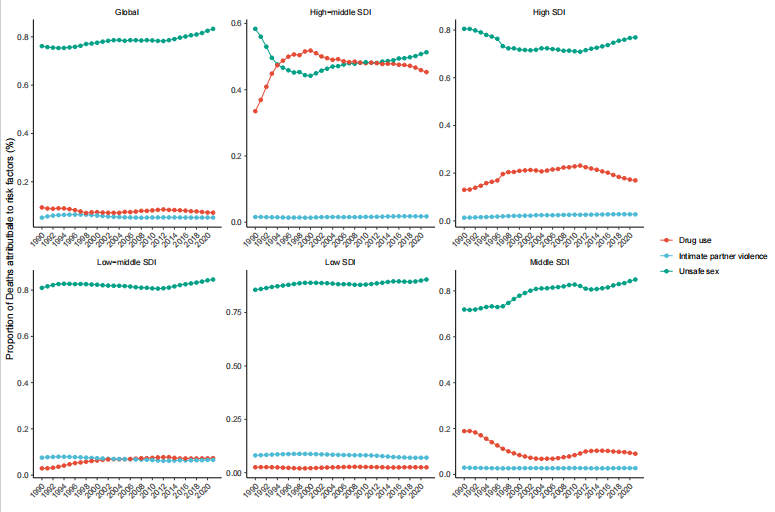


B

A

C


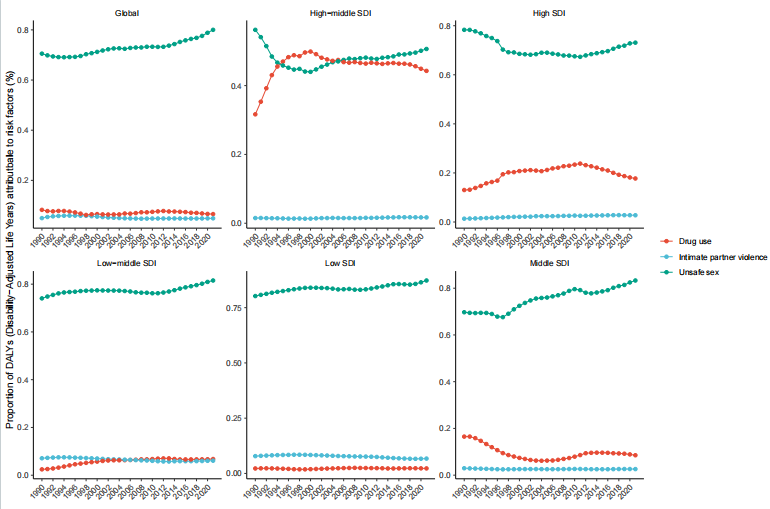

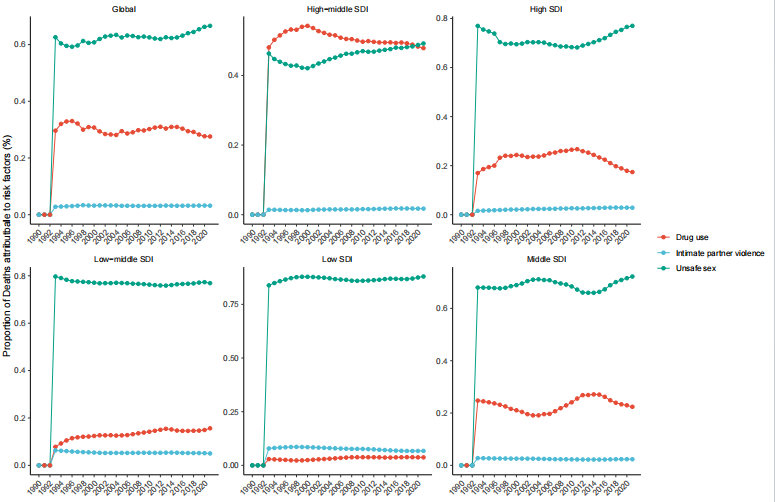

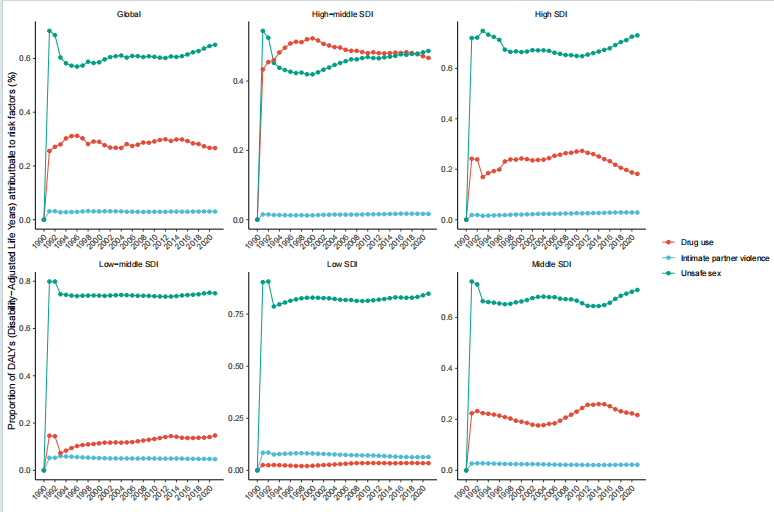


D

F

E

Fig. S15 The association between risk factors and the age-standardized mortality rate, age-standardized DALY rate of HIV-DS-TB, HIV-MDR-TB, HIV-XDR-TB from 1990 to 2021 (A: Mortality rate of HIV-DS-TB. B: DALY rate of HIV-DS-TB. C: Mortality rate of HIV-MDR-TB. D: DALY rate of HIV-MDR-DS-TB. E: Mortality rate of HIV-XDR-TB. F: DALY rate of HIV-XDR-TB. DALYs: disability-adjusted life years. HIV-DS-TB: HIV-infected drug-susceptible tuberculosis. HIV-MDR-TB: HIV-infected multidrug-resistant tuberculosis without extensive drug resistance. HIV-XDR-TB: HIV-infected extensively drug-resistant tuberculosis).
